# Aqp5 Is a New Transcriptional Target of Dot1a and a Regulator of Aqp2

**DOI:** 10.1371/journal.pone.0053342

**Published:** 2013-01-10

**Authors:** Hongyu Wu, Lihe Chen, Xi Zhang, Qiaoling Zhou, Ju-Mei Li, Stefan Berger, Zea Borok, Beiyun Zhou, Zhou Xiao, Hongling Yin, Mingyao Liu, Ying Wang, Jianping Jin, Michael R. Blackburn, Yang Xia, Wenzheng Zhang

**Affiliations:** 1 Department of Internal Medicine, The University of Texas Health Science Center at Houston, Houston, Texas, United States of America; 2 Graduate School of Biomedical Sciences, The University of Texas Health Science Center at Houston, Houston, Texas, United States of America; 3 Department of Internal Medicine, Xiangya Hospital, Central South University, Changsha, Hunan, China; 4 Department of Biochemistry and Molecular Biology, The University of Texas Health Science Center at Houston, Houston, Texas, United States of America; 5 German Cancer Research Center, Division Molecular Biology of the Cell I, Heidelberg, Germany; 6 Will Rogers Institute Pulmonary Research Center, Division of Pulmonary and Critical Care Medicine, Department of Medicine, University of Southern California, Los Angeles, California, United States of America; 7 Institute of Biosciences and Technology and Department of Molecular and Cellular Medicine, Texas A&M University System Health Science Center, Houston, Texas, United States of America; National Cancer Institute, United States of America

## Abstract

*Dot1l* encodes histone H3 K79 methyltransferase Dot1a. Mice with *Dot1l* deficiency in renal Aqp2-expressing cells (*Dot1l^AC^*) develop polyuria by unknown mechanisms. Here, we report that Aqp5 links *Dot1l* deletion to polyuria through Aqp2. cDNA array analysis revealed and real-time RT-qPCR validated *Aqp5* as the most upregulated gene in *Dot1l^AC^* vs. control mice. Aqp5 protein is barely detectable in controls, but robustly expressed in the *Dot1l^AC^* kidneys, where it colocalizes with Aqp2. The upregulation of Aqp5 is coupled with reduced association of Dot1a and H3 dimethyl K79 with specific subregions in Aqp5 5′ flanking region in *Dot1l^AC^* vs. control mice. In vitro studies in IMCD3, MLE-15 and 293Tcells using multiple approaches including real-time RT-qPCR, luciferase reporter assay, cell surface biotinylation assay, colocalization, and co-immunoprecipitation uncovered that Dot1a represses Aqp5. Human AQP5 interacts with AQP2 and impairs its cell surface localization. The AQP5/AQP2 complex partially resides in the ER/Golgi. Consistently, AQP5 is expressed in none of 15 normal controls, but in all of 17 kidney biopsies from patients with diabetic nephropathy. In the patients with diabetic nephropathy, AQP5 colocalizes with AQP2 in the perinuclear region and AQP5 expression is associated with impaired cellular H3 dimethyl K79. Taken together, these data for the first time identify Aqp5 as a Dot1a potential transcriptional target, and an Aqp2 binding partner and regulator, and suggest that the upregulated Aqp5 may contribute to polyuria, possibly by impairing Aqp2 membrane localization, in *Dot1l^AC^* mice and in patients with diabetic nephropathy.

## Introduction

In addition to glucosuria, polyuria is the earliest clinical renal symptom in untreated or poorly controlled diabetes [1] and is not considered as a simple result of an osmotic diuresis due to the large solute load of urinary glucose [2], [3]. However, the molecular mechanism(s) by which polyuria develops beyond glucosuria is not fully understood.

Aquaporins (AQPs) are members of the water channel family. Aqp1- 4 are important for maintenance of normal urinary concentration and implicated in the renal water disorders [4]–[7]. Reduced expression and/or apical localization of Aqp2 under pathological conditions (i.e. nephrosis, hypokalemia, and *Aqp2* mutations) results in polyuria. In contrast, nephrotic syndrome and congestive heart failure due to abnormal secretion of vasopressin increase apical Aqp2 levels, leading to excessive water reabsorption and hyponatremia (reviewed in [8]).

Aqp5 is expressed in eyes, salivary glands, lung and sweat glands [9]–[11]. A selective defect in lacrimal gland Aqp5 trafficking is responsible for Sjögren's syndrome characterized by dry eye and mouth [12]. While Aqp5 and Aqp2 are the closest homologs and share 66% sequence identity, Aqp5 is undetectable in normal mouse kidney by Northern analysis and immunoblotting (IB) [13].

Disruptor of telomeric silencing (*Dot1*) was first discovered in yeast to affect telomeric silencing [14]. *Dot1* and its mammalian homologs (*Dot1l*) encode a methyltransferase specific for histone H3 K79 [15]–[17]. *Dot1l* is critical in embryogenesis [18], hematopoiesis [19], [20], cardiac function [21], and leukemogenesis [20], [22], [23]. Dot1l transcripts are abundant in mouse kidney and contain five alternative splicing variants (Dot1a-e) [17]. Dot1a binds Af9 and represses several aldosterone-upregulated genes including *αENaC* and *preproendothelin-1* [24]–[26]. Under basal conditions, Dot1a-Af9 binds specific subregions of *αENaC* promoter, promotes H3 di-methyl K79 (H3m2K79), and inhibits transcription [24], [27]. Aldosterone reduces Dot1a and Af9 and induces Sgk1 that impairs Dot1a interaction with Af9 by phosphorylating Af9 [28]. Despite these observations, the role of *Dot1l* in renal water homeostasis has not been described.

Recently, we have reported generation of a *Dot1l* conditional knockout line using the LoxP-Cre system (*Dot1l^f/f^*), which inactivate most of *Dot1l* function including the methyltransferase activity upon Cre-mediated recombination [23]. This line was used to generate connecting tube/collecting duct (CNT/CD)-specific *Dot1l*-deficient (*Dot1l^f/f^ Aqp2:Cre* or *Dot1l^AC^*) mice by crossing them with *Aqp2:Cre* mice [29], which drive Cre recombinase expression under the control of regulatory elements of the mouse *Aqp2* gene. Generation and characterization of *Dot1l^AC^* have been detailed in our recent manuscript [30]. Compared to *Dot1l^f/f^* controls, *Dot1l^AC^* mice have polyuria without severe impairment in maintaining normal electrolyte and acid-base balance [30]. In this report, we provide strong in vivo and in vitro evidence for the first time demonstrating that Dot1a downregulates Aqp5 and Aqp5 interacts with Aqp2 and impairs Aqp2 membrane localization. We also observed upregulated AQP5 and decreased H3m2K79 in kidney biopsies from patients with diabetic nephropathy (DN). The polyuria phenotype in *Dot1l^AC^* mice and in patients with DN may be partially attributable to upregulated Aqp5.

## Results

### *Dot1l* ^AC^ mice displayed polyuria without impaired Aqp2 expression

Generation of *Dot1l^AC^* mice and description of their polyuria phenotype on a normal pellet Na^+^ diet are detailed in our related manuscript [30]. Briefly, we used a *Dot1l* conditional knockout line (*Dot1l^f/f^*) [23] and an *Aqp2Cre* line [29] to inactivate *Dot1l* and thus abolish histone H3 K79 methylation in Aqp2-expressing cells, which are located in the CNT/CD [30].

**Figure 1:**
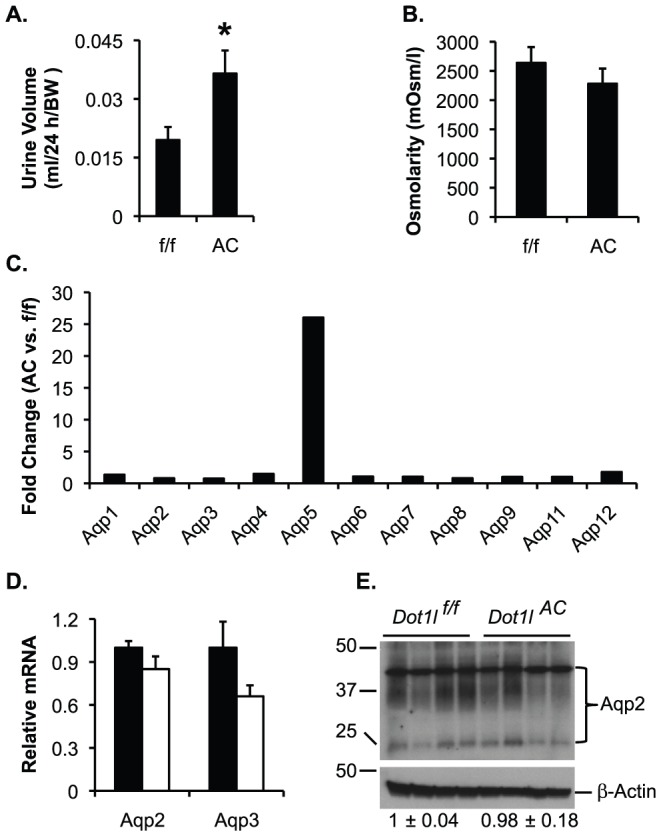
*Dot1l* ^AC^ mice displayed polyuria without impaired Aqp2 expression. (**A–B**). *Dot1l^f/f^* and *Dot1l^AC^* mice deprived of water for 24 h (n = 14 mice/group) were subjected to 24-h urine analyses as indicated. For additional measurements, see Figure S1. *P<0.05 vs. *Dot1l^f/f^*. (**C**) *Dot1l^AC^* vs. *Dot1l^f/f^* mice (n = 4 mice/group, see Materials and Methods) have only subtle changes in the expression of all known Aqps as indicated except for Aqp5. Shown are the fold changes revealed by cDNA array anlalysis. (**D**) Real-time RT-qPCR for expression of Aqp2 and AQP3 in kidney of mice fed the normal Na^+^ pellet diet, with β-actin as internal control. n = 6 mice/group. (**D**) IB for Aqp2 expression, with β-actin as internal control. n = 4 mice/group.

To further confirm the polyuria phenotype, we performed additional metabolic analysis. *Dot1l^AC^* vs. *Dot1l^f/f^* littermates after 24-h water deprivation (n = 14 mice/group) showed significantly increased normalized (to body weight) and slightly decreased urine osmolarity (Figure 1A–B). Excretion of Na^+^ and K^+^ was 155±16% and 146±17% of *Dot1l^f/f^* mice, respectively, in *Dot1l^AC^* after the 24-h water deprivation. There were subtle, but not significant differences in all other urinary parameters ([Na^+^], [K^+^], [creatinine], [Na^+^]/[creatinine], [K^+^]/[creatinine]) tested between the two groups (Figure S1). The absolute urine volume was also significantly increased by 73%, 63% and 465% in *Dot1l^AC^* vs. *Dot1l^f/f^* mice in fed state, after 24-hour water deprivation, and after Streptozotocin (STZ)-induced diabetes, respectively (Figure S2).

### Microarray analysis identified Aqp5 as the most upregulated gene in *Dot1l^AC^* mice

To assess the effect of *Dot1l* inactivation on global gene expression and to identify the molecular defects leading to polyuria, we performed gene expression microarray analysis of *Dot1l^AC^* vs. *Dot1l^f/f^* mice (n = 4 mice/group), using the dual-color Agilent 4X44K Whole Mouse Genome Array system. With a minimal two-fold difference between the two genotypes as an arbitrary cut-off, we found 1359 up- and 627 down-regulated genes, respectively, in *Dot1l^AC^* vs. *Dot1l^f/f^* mice (Table S1 and S2). However, all of the known Aqps except Aqp5 had no or only subtle changes in their mRNA levels (Figure 1C). Real-time RT-qPCR validated that the two genotypes were indistinguishable in the expression of Aqp2 and Aqp3, two important water channels in the Aqp2^+^ principal cells, where *Dot1l* deletion occurs (Figure 1D). Immunoblotting analysis (IB) confirmed comparable Aqp2 protein levels between the two groups (Figure 1E).

**Figure 2:**
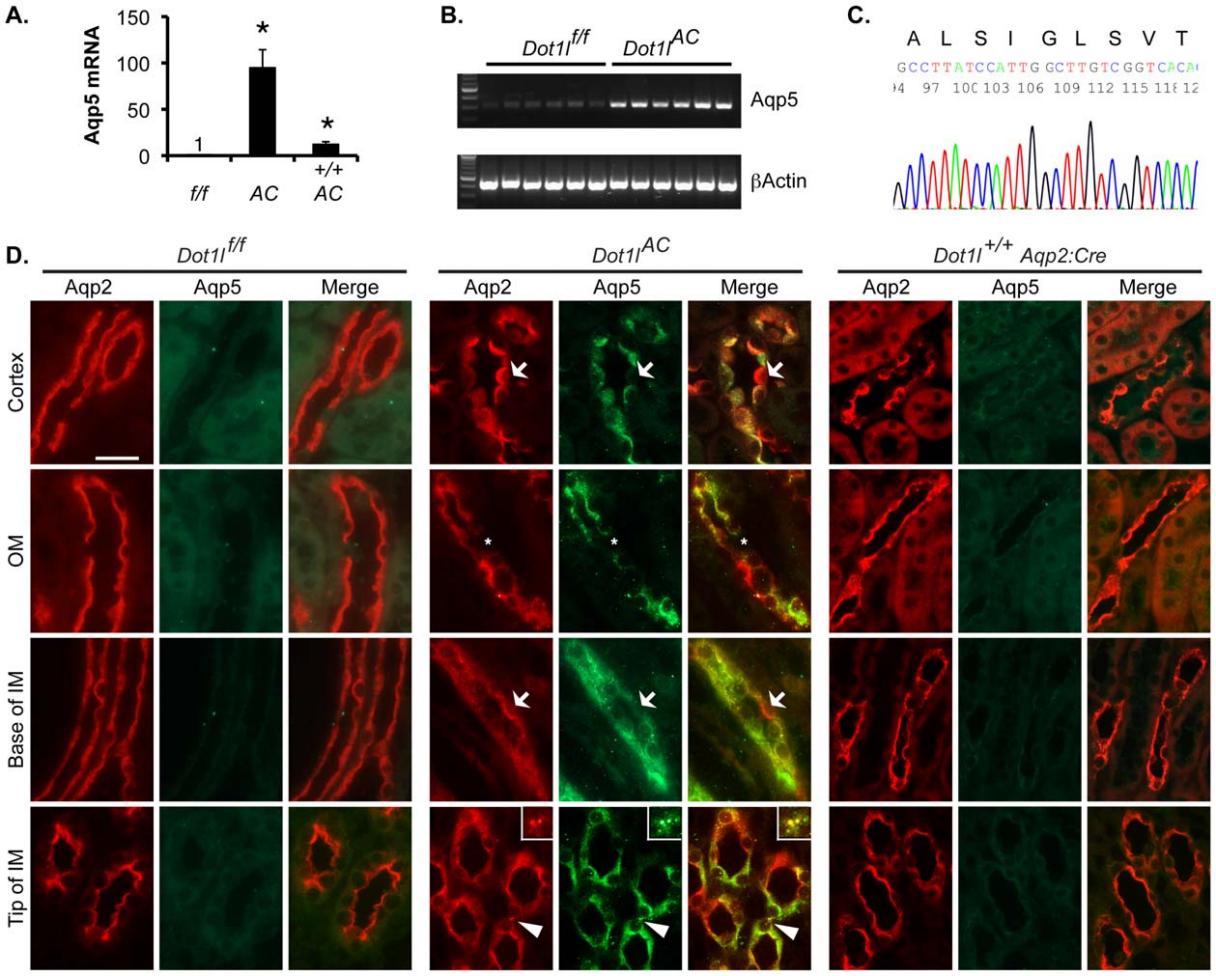
Aqp5 is significantly upregulated and coexpressed with Aqp2 in the kidney of *Dot1l^AC^* mice on the normal Na^+^ pellet diet. (**A**) Real-time RT-qPCR for expression of Aqp5 in kidney of mice fed the normal Na^+^ pellet diet, with β-actin as internal control. n = 6 mice/group. *+/+AC*: *Dot1l^+/+^Aqp2:Cre* (**B**) As in **A**, agarose gel analysis of the final RT-qPCR products of Aqp5 and β-actin. (**C**) Sequencing of a regular RT-PCR product from a *Dot1l^f/f^* mouse kidney. A part of the tracing file showing Aqp5 sequence encoding aa 47–55 (GenBank#: EDL04123.1) is given. (**D**) Representative IF images showing Aqp5 (green) expression in Aqp2^+^ (red) cells in mice as indicated. OM and IM: outer and inner medulla. *: An IC without Aqp5 expression, possibly due to lack of Aqp2Cre-mediated *Dot1l* ablation. Arrow: PC with strong Aqp2 and weak Aqp5, highlighting the lack of cross reactivity of the two antibodies. Aqp5^+^Aqp2^−^ cells are most likely the intercalated cells derived from the Aqp2-expressing progenitor cells or mature PC [30]. Arrowhead: Colocalization of Aqp5 with Aqp2, which is amplified in the inserts. Scale bar: 50 µM. For more images with lower magnification, see Figure S3.

*Aqp5* is the closest homolog of and adjacent to *Aqp2* in the genome. *Dot1l^AC^* and *Dot1l^+/+^ Aqp2Cre* mice have two copies of the *Aqp2Cre* transgene, which carries the adjacent *Aqp5* [29]. If Aqp5 expression were solely copy-number-dependent, Aqp5 would be 2-fold higher in these animals than in *Dot1l^f/f^* mice. Unexpectedly, microarray analyses revealed Aqp5 as the most upregulated gene, with 26-fold higher mRNA level in *Dot1l^AC^* than in *Dot1l^f/f^* mice (Figure 1C). Real-time RT-qPCR unearthed an even bigger difference (105-fold). Aqp5 mRNA level was also significantly increased in *Dot1l^+/+^ Aqp2Cre* vs. *Dot1l^f/f^* mice. However, such increase was much less prominent, compared to *Dot1l^AC^* mice (Figure 2A). Subsequent agarose gel analysis revealed a single band from each of these RT-qPCR reactions (Figure 2B). Sequencing analyses of a regular RT-PCR product from *Dot1l^f/f^* mice confirmed the identity of Aqp5 (Figure 2C). This very low level of Aqp5 mRNA may explain why Aqp5 was undetectable by Northern and IB [13] and by immunofluorescence (IF) in normal mouse kidney (Figure 2D).

### Aqp5 is co-expressed with Aqp2 in *Dot1l^AC^* mice

As shown in Figure 2D and Figure S3 and S4, IF with goat anti-Aqp2 (as the marker of PCs in which *Dot1l* deletion occurs) and rabbit anti-Aqp5 antibodies revealed that *Dot1l^f/f^* mice had robust Aqp2 and undetectable Aqp5 throughout the whole kidney. Very faint Aqp5 staining can be seen in *Dot1l^+/+^ Aqp2Cre* mice. Nevertheless, Aqp5 was readily detected and coexpressed with Aqp2 in most, but not all of the PC in *Dot1l^AC^* mice. The lack of Aqp5 in some of PC may result from absence of Cre expression [30], leaving *Dot1l* intact. Substantial Aqp5 was also visible in some of Aqp2^−^ cells, which are most likely the intercalated cells derived from the canonical Aqp2-expressing cells as detailed in our related manuscript [30]. The affinity-purified anti-Aqp5 antibody was produced using a synthetic peptide corresponding to residues of 251–265 of rat Aqp5. This sequence is identical to mouse Aqp5 and has no significant homology to mouse Aqp2. This may explain why the Aqp5 antibody lacks the cross reactivity with mouse Aqp2 (Figures 2D, S3, S4). The anti-Aqp5 antibody has been used to detect Aqp5 in different species including mouse [31] and duckling [32].

### Aqp5 is expressed in developing *Dot1l^AC^* kidneys

To identify when Aqp5 is expressed during kidney development, we examined Aqp2 and Aqp5 co-expression in the kidneys from 3-, 11-, and 20-day old pubs. Like in adult *Dot1l^f/f^* mice, Aqp5 remained undetectable in all of the developing *Dot1l^f/f^* kidneys (Figure S4). In *Dot1l^AC^* mice, Aqp5 was barely discernable at day 3, but became prominent at day 11 and day 20 in some Aqp2^+^ and Aqp2^−^ connecting tube/collecting duct cells, as observed in the adult stage (Figure S4).

### AQP5 is pathologically expressed and colocalizes with AQP2 at the perinuclear region in patients with DN

**Figure 3:**
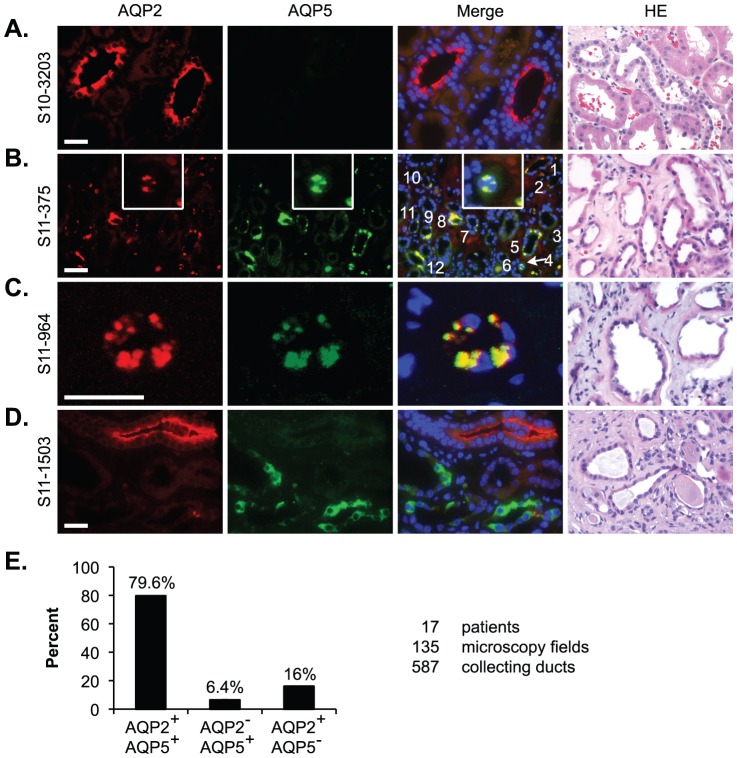
AQP5 is pathologically expressed and colocalizes with AQP2 at the perinuclear region in patients with DN. (**A**–**D**) Representative IF images of kidney biopsies from a patient with MCD showing no detectable AQP5 (**A**), from two patients with DN showing AQP5 colocalization with AQP2 at perinuclear region (**B–C**), and from a patient with DN showing diffuse AQP5 without AQP2 in the same cells (**D**). There are 12 AQP2^+^AQP5^+^ tubules in **B**. Three detached cells are shown in the insert. Hematoxylin/eosin staining verified no significant pathological changes in the tubules of the MCD samples, but various tubular abnormalities in all of the DN samples (see text for details). (**E**) Summary of the relative abundance of the three types of tubules from 17 patients with DN. Scale bar: 50 µM.

While polyuria in DN is clearly due to glucosuria, we hypothesize that abnormal co-expression of AQP5 with AQP2 occurs in patients with DN, which may make the polyuria phenotype even more prominent. Accordingly, we investigated AQP5 and AQP2 expression by IF in kidney biopsies from 17 patients with DN and 15 with minimal change disease (MCD). Since these MCD samples had no significant pathological changes in the tubules, as revealed by Hematoxylin-Eosin staining (Figure 3A), they were considered as “normal” controls. All of the MCDs had undetectable AQP5, with AQP2 primarily seen at the apical side (Figure 3A). Unlike MCDs, all DN samples showed various tubular abnormalities including dilated lumen, epithelial thinning, nuclear irregularity, eosinophilic cytoplasm, severe tubular basement membrane thickening, interstitial fibrosis, and mononuclear cell infiltrate (Figure 3, B–D). We focused on the tubules containing at least one AQP5^+^ or AQP2^+^ cell. The tubules containing only AQP2^+^- AQP5^+^- or double positive cells were categorized as AQP2^+^AQP5^−^, AQP2^−^AQP5^+^, and AQP2^+^AQP5^+^, respectively. The AQP2^−^AQP5^−^ tubules were excluded from analyses because of the uncertainty of their identities. In the AQP2^+^AQP5^+^ tubules, these two proteins were typically colocalized as large discrete foci near the perinuclear region (Figure 3, B & C). In AQP2^−^AQP5^+^ tubules, however, AQP5 distribution was broader and more homogenous (Figure 3D). The detection of AQP2^+^AQP5^−^ and AQP2^−^AQP5^+^ tubules/cells demonstrated the specificity of each antibody used.

To evaluate the relative abundance of the three types of tubules, we counted their numbers in each field containing at least one AQP5^+^ or AQP2^+^ cell. Among 135 images from the 17 DN samples, 587 tubules were counted, yielding 79.6% AQP2^+^AQP5^+^, 16% AQP2^+^AQP5^−^, and 6.4% AQP2^−^AQP5^+^. A single image containing 12 AQP2^+^AQP5^+^ tubules is shown in Figure 3B. Our data for the first time strongly suggest that AQP5 is pathologically expressed in renal PC and may inhibit AQP2 membrane localization by “trapping” it near the perinuclear region in DN patients (Figure 3B and 3C).

### AQP5 expression is inversely correlated with H3m2K79 in DN patients

To test the hypothesis that the pathological expression of AQP5 is associated with loss of H3m2K79 in DN patients, similar double IF was conducted to examine coexpression of AQP5 and H3m2K79. We chose H3m2K79 as an indicator of DOT1L function because all available antibodies against DOT1L failed to detect the target protein specifically by IF ([33] and data not shown) and *Dot1l* is solely responsible for H3m2K79 in mouse kidney [30]. Moreover, H3m2K79 was used to assess inactivation of *Dot1l* function and thus disrupted *Dot1l* expression in the *Dot1l*-deficient embryos [18], in the heart of the cardiac-specific *Dot1l* knockout mice [21] or in the peripheral blood nucleated cells of *Dot1l^f/f^ Vav-Cre* mice [34].

**Figure 4:**
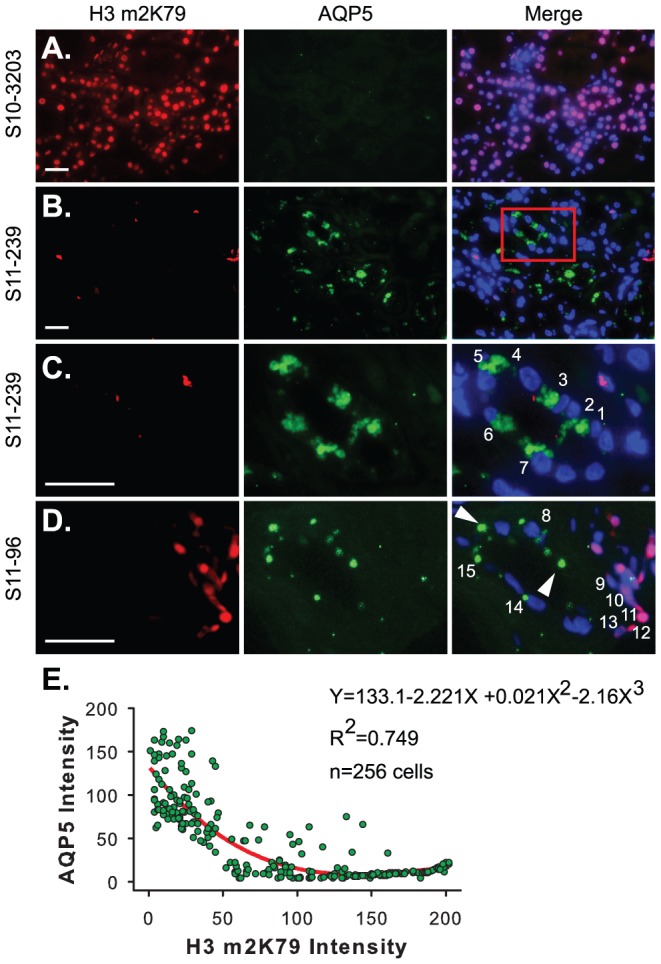
AQP5 expression is inversely correlated with H3m2K79 in DN. (**A**–**D**) Representative IF images of kidney biopsies from a patient with MCD showing strong H3m2K79 and undetectable AQP5 (**A**) and from two patients with DN showing significantly reduced H3m2K79 in most of the cells with some of them robustly expressing AQP5 (**B–D**). The boxed area in **B** was amplified and shown in **C**. The presence of large discrete foci was exclusively used to score AQP5 positive staining. If an AQP5^+^ cell has a clear DAPI-stained nucleus as shown in **C** and **D**, they are numbered. (**E**) The pixel intensities of AQP5 and H3m2K79 measured simultaneously, using ImageJ64. Arrowheads in **D** indicate ignored AQP5^+^ spots due to absence of a nucleus showing the status of H3m2K79. Scale bar: 50 µM.

All MCDs had strong H3m2K79 staining and apparently no AQP5 throughout the biopsies. However, all of the DN samples significantly reduced H3m2K79 labeling throughout the whole biopsies in some cases or focally in others. Most of the AQP5^+^ cells displayed weak or no H3m2K79 labeling at all in their nuclei (Figure 4, A–D). Within the tubules containing AQP5^+^ H3m2K79^−^ cells, some cells apparently were negative for both antibodies (AQP5^−^ H3m2K79^−^). This suggests that loss of H3m2K79 is not always associated with “de-silencing” AQP5.

To more accurately assess the inverse correlation between AQP5 and H3m2K79, we measured their pixel intensities in 256 cells from all of the DN samples (see Materials and Methods). The data were best fit with a curve Y = 133.1−2.221X+0.021X^2^−2.16X^3^, which yielded a determination coefficient (R^2^) = 0.749 (Figure 4E). Therefore, our data strongly suggest an inverse correlation between the two parameters in DN patients, that MCDs express high H3m2K79 without detectable AQP5 labeling, and that the undetectable H3m2K79 is not due to a general defect in protein synthesis and/or stability since robust Aqp5 expression can occur in the same cells in the patients with DN.

### *Dot1l^AC^* vs. *Dot1l^f/f^* mice displayed impaired Dot1a binding and reduced H3m2K79 at the *Aqp5* 5′ regulatory region

**Figure 5:**
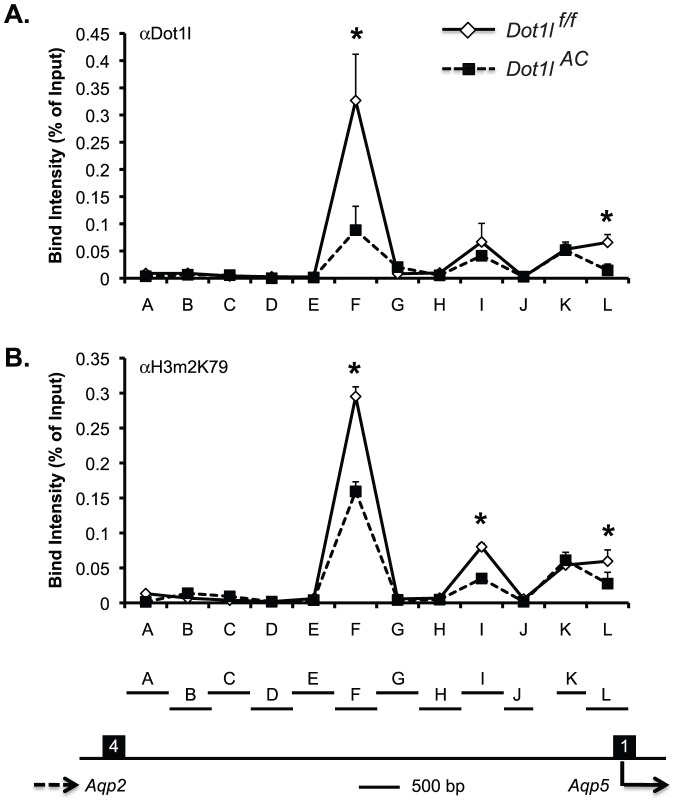
ChIP demonstrates impaired binding of Dot1l and H3m2K79 at specific subregions of Aqp5 5′ regulatory region. Chromatin from *Dot1l^f/f^* and *Dot1l^AC^* mice on the normal Na^+^ pellet diet (n = 6 mice/group) was immunoprecipitated by the rabbit antibodies specific for Dot1l (**A**) and H3m2K79 (**B**), followed by real-time qPCR with primers amplifying subregions A-L covering almost the whole region between the end of Aqp2 exon 4 and the translation start site ATG of Aqp5 as shown at the bottom. There is a 525-bp gap between J and K, which cannot be reliably amplified and was excluded from further analysis. Relative ChIP efficiency was defined as the immunoprecipitated amount of materials present as compared to that of the initial input sample. *: P<0.05 vs. *Dot1l^f/f^* within the same subregion.

To determine if *Dot1l* regulates *Aqp5* transcription through modulation of H3m2K79 at the *Aqp5* 5′ flanking region, chromatin immunoprecipitation (ChIP) assay coupled with real-time qPCR was pursued. We focused on the 6.5 kb region that spans from the very end of the last exon (exon 4) of *Aqp2* to the translation start site ATG of *Aqp5*. The whole region was divided into 12 subregions named A-L (Figure 5, A–B). ChIP with anti-Dot1l or anti-H3m2K79 detected substantial signals in F, I, K and L, with the strongest binding in F in *Dot1l^f/f^* mice. Such signals were significantly attenuated, but still detectable in *Dot1l^AC^* mice, possibly due to the fact that deletion of *Dot1l* occurs only in Aqp2-expressing cells, rather than in all cells in the kidney. All other subregions showed no or weak ChIP signals in both genotypes. These observations collectively reinforce the specificity of Dot1l and H3m2K79 antibodies that have been used by others and us [24], [27], [28], [35], [36]. Our data also suggest that the association of Dot1a and H3m2K79 with some, but not all of the subregions is not simply due to non-specific DNA binding activity as reported for hDOTL1(1–416) in vitro [37]. Furthermore, ChIP with normal rabbit IgG yielded barely detectable background signals (data not shown). These data suggest that Dot1l downregulates Aqp5 mRNA expression, possibly by modulating H3m2K79 at the *Aqp5* promoter.

### Dot1a decreases Aqp5 expression in M1 and MLE-15 cells

**Figure 6:**
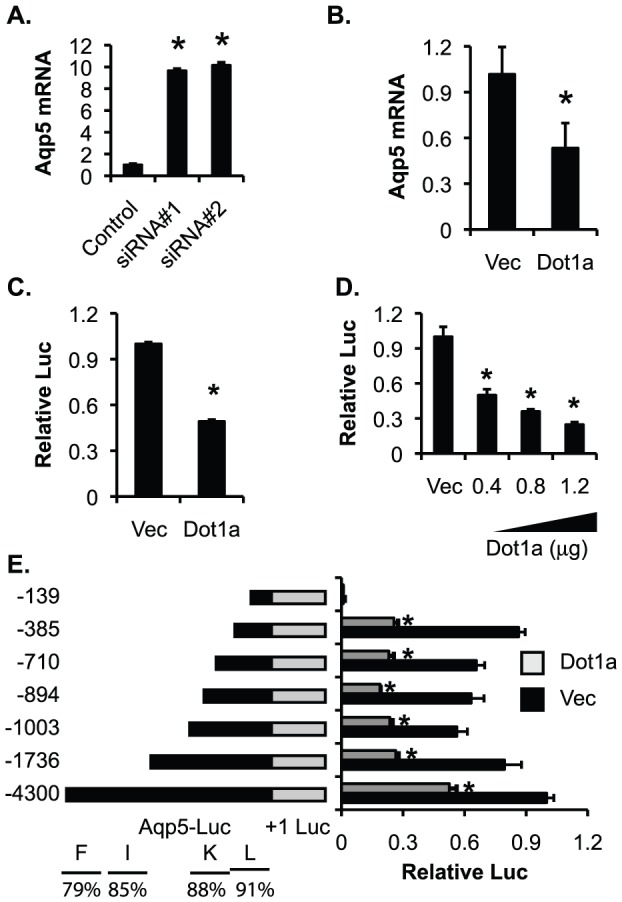
Dot1a represses Aqp5 mRNA expression in mouse cortical collecting duct M1 and mouse lung epithelial MLE-15 cells. (**A–B**) Real-time RT-qPCR showing that siRNA-mediated depletion of Dot1la in M1 cells increases endogenous Aqp5 expression (**A**) and that overexpression of Dot1a decreases endogenous Aqp5 expression in MLE-15 cells (**B**). (**C**) Luciferase assays showing that overexpression of Dot1a reduces expression of a luciferase reporter driven by a 4.3-kb (−4300 in **E**) promoter of rat Aqp5 in MLE-15 cells. (**D**) As in **C**, except that various amounts of Dot1a plasmid were used for transfection. (**E**) Luciferase assay showing that Dot1a represses Aqp5 promoter-luciferase constructs as indicated. The relative positions of the Dot1l- and H3m2K79-binding subregions of mouse Aqp5 in ChIP assays were shown at the bottom. The percentages of identities between rat and mouse were also indicated. Note: Dot1a-mediated repression was eliminated in the −139 construct.

To further evaluate whether Dot1a inhibits Aqp5 promoter activity in cultured cells, mouse cortical collecting duct M1 cells were transfected with two Dot1a-specific siRNAs: siRNA#1 and siRNA#2. Real-time RT-qPCR revealed that cells transfected with these two siRNAs increased Aqp5 mRNA to 964% and 1014% of control (Figure 6A). Since M1 cells have a very low basal level of Aqp5 expression, overexpression of Dot1a apparently further reduced Aqp5 mRNA abundance. This was envisioned by non-specific rather than specific amplification, which made the real-time RT-qPCR measurements unreliable. Accordingly, we chose mouse lung epithelial MLE-15 cells that robustly express Aqp5 to more accurately investigate the effect of Dot1a overexpression. Dot1a-overexpressing vs. control cells showed significantly decreased expression of endogenous Aqp5 and activity of a luciferase reporter driven by a 4.3-kb upstream fragment of rat *Aqp5* [38]. Luciferase activity was progressively decreased with increasing amounts of Dot1a constructs (Figure 6, B–D). Sequential deletion analysis revealed that the fragment beginning at −385 bp was apparently capable of and sufficient for Dot1a-mediated downregulation (Figure 6E). However, the smallest construct starting at −139 displayed just background luciferase activity with or without Dot1a overexpression. Therefore, Dot1a attenuates Aqp5 expression, possibly by direct or indirect binding to the *Aqp5* promoter and modulating H3m2K79 associated with the *Aqp5* promoter in M1 and MLE-15 cells and in mouse kidney.

### AQP5 coimmunoprecipitates with AQP2

**Figure 7:**
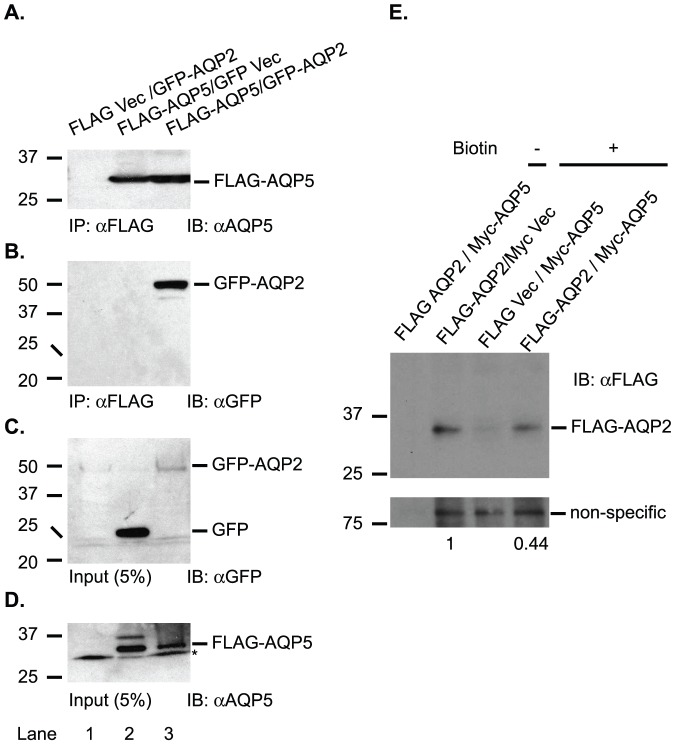
AQP5 coimmunoprecipitates with AQP2 and impairs its cell surface localization in IMCD3 cells. (**A–D**) Co-IP showing human AQP5 interacts specifically with human AQP2. AQP5 and AQP2 were expressed as FLAG- or GFP- fusions in 293T cells, and analyzed by IP-IB with antibodies as indicated. (**E**) Representative IBs of biotinylation assay showing that AQP5 impairs cell surface localization of AQP2. FLAG-AQP2 and Myc-AQP5 were expressed separately or in combination in IMCD3 cells, biotinylated, and analyzed by IB with antibodies as indicated. *: unknown protein.

Colocalization of Aqp5 with Aqp2 in *Dot1l^AC^* kidney and in patients with DN suggests interactions between these proteins. To solidify this finding, we conducted co-immunopreciptation assays with green fluorescence protein (GFP)-AQP2 and FLAG-AQP5 expressed in 293T cells. FLAG-AQP5 was immunoprecipitated from its transfectants, but not from FLAG Vec-transfectants, confirming specificity of the FLAG antibody used for immunoprecipitation (Figure 7A). Co-immunoprecipitation of GFP-AQP2 and FLAG-AQP5 occurred when they were co-expressed. Replacing one of them with the corresponding vector abolished the coimmunoprecipitation (Figure 7, A–B). In all cases, GFP-AQP2 and FLAG-AQP5 fusions were expressed at comparable levels among transfections (Figure 7, C–D).

### AQP5 impairs AQP2 cell surface expression

Colocalization of AQP5 with AQP2 at the perinuclear region in patients with DN suggests that AQP5 may reduce AQP2 cell surface abundance. To test this hypothesis, we performed cell surface biotinylation assays using FLAG-AQP2 and Myc-AQP5 expressed in IMCD3 cells. Compared with Myc vector, Myc-AQP5 significantly decreased plasma membrane-associated FLAG-AQP2 to about 44% (Figure 7E). When biotin labeling was omitted, plasma membrane-associated FLAG-AQP2 became undetectable, confirming the specificity of biotinylation (Figure 7E).

### AQP5 and AQP2 colocalize and partially reside in the ER/Golgi in IMCD3 cells

**Figure 8:**
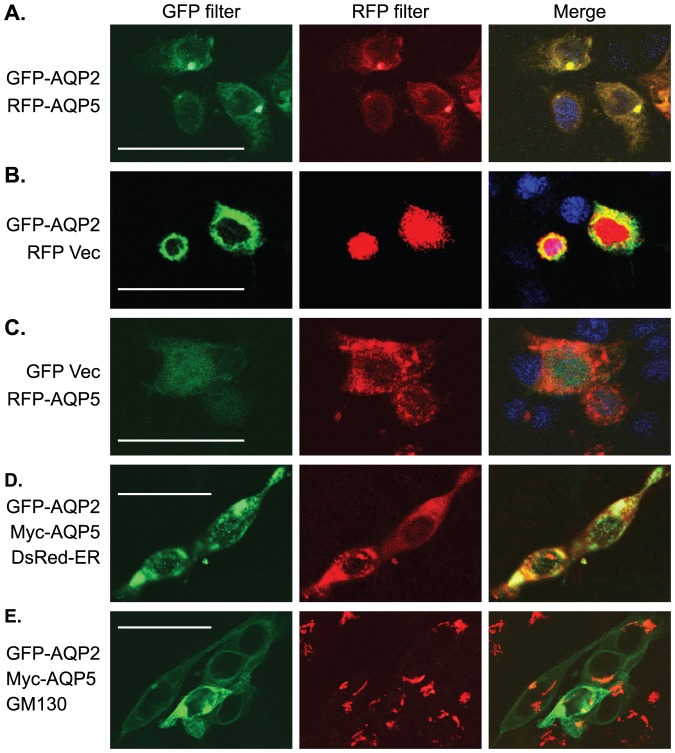
AQP5 and AQP2 colocalize and partially reside in the ER/Golgi compartments in IMCD3 cells. (**A**–**C**) Representative confocal microscopy images showing colocalization of RFP-AQP5 and GFP-AQP2 (**A**), lack of colocalization when GFP-AQP2 (**B**) and RFP-AQP5 (**C**) was coexpressed with RFP and GFP alone, respectively. (**D**) Representative confocal microscopy images showing AQP5-AQP2 complex partially locates in the ER in IMCD3 cells. IMCD3 cells were co-transfected with the plasmids as indicated and examined by confocal microscopy. DsRed-ER was used as an endoplasmic reticulum (ER) marker [39] and shown in red. GFP-AQP2 is shown in green. Myc-AQP5 was not directly detected, but can be inferred by its interaction and colocalization with AQP2 as large discrete foci seen in **A**. AQP2 alone seldom forms large discrete foci. In addition, more than 95% of transfected IMCD3 cells were always doubly transfected. Therefore, the presence of the large discrete foci is indicative of AQP2-AQP5 complex. (**E**) As in D except that DsRed-ER was omitted and cells were stained with an antibody specific for GM130, a cis-Golgi marker. Scale bar: 40 µM.

Like in patients with DN (Figure 3, B & C), colocalization of AQP2 and AQP5 as large discrete foci in the perinuclear region was also observed when they were co-expressed as GFP and red fluorescence protein (RFP) fusions in IMCD3 cells and examined by confocal microscopy. The colocalization was apparently not a result of overexpression because neither GFP-AQP2 nor RFP-AQP5 colocalized with overexpressed RFP or GFP alone, respectively (Figure 8, A–C). Furthermore, over 95% of transfected cells were doubly transfected. GFP-AQP2 seldom formed large discrete foci in the absence of RFP-AQP5. Based on these two observations, we wanted to determine if AQP2/AQP5 complexes are localized in the ER/Golgi.

IMCD3 cells were co-transfected with GFP-AQP2, Myc-AQP5 and DsRed-ER as endoplasmic reticulum marker [39]. Alternatively, the cells were transfected without DsRed-ER and stained with an antibody specific for the cis-Golgi marker GM130. Confocal microscopy revealed that GFP-AQP2/Myc-AQP5 complex evidenced by large discrete foci partially colocalized with the markers, suggesting that some of the complexes were targeted to and/or retained in ER and cis-Golgi compartments (Figure 8, D & E).

## Discussion

In this report, we uncover a new mechanism by which *Dot1l* regulates water homeostasis, and identify Aqp5 as a potential novel target of Dot1a, a binding partner of Aqp2, a negative trafficking regulator of Aqp2, and thus the potential missing component linking disrupted *Dot1l* to polyuria.

We provide evidence showing that *Aqp5* is a new Dot1a-regulated gene. Genetic inactivation, and siRNA-mediated depletion of Dot1l led to endogenous *Aqp5* upregulation. The reverse correlation of AQP5 expression with H3m2K79 in DN further suggests the importance of H3m2K79 in this regulation. In contrast, Dot1a overexpression inhibited expression of endogenous Aqp5 and all but the smallest *Aqp5* promoter-luciferase constructs. These results suggest that exogenously expressed constructs and the endogenous *Aqp5* responded similarly to changes in Dot1a abundance. Unlike the −389 construct, the −139 construct apparently lacks Dot1a-mediated repression and basal promoter activity, indicating the existence of a critical “Dot1a-responsible” cis-element and elements essential for the minimal promoter activity between −389 and −139 region.

TNFα and lipopolysaccharide inhibit *Aqp5* expression in salivary and parotid gland cells, respectively [40], [41]. TNFα-mediated repression involves suppression of histone H4 acetylation [40]. Lipopolysaccharide enhances NF-κB, but not AP-1 binding at the *Aqp5* promoter [41]. Interestingly, the AP-1 and NF-κB sites reside in subregions K and L, which were shown to bind Dot1l and H3m2K79 in ChIP assays (Figure 5). Therefore, it would be interesting to know how the binding of these transcription factors is coordinately regulated in the *Aqp5* promoter, and to investigate if the 4 subregions (F, I, K and L) are critical to an insulator element between *Aqp2* and *Aqp5*. Dot1a may also indirectly inhibit Aqp5 expression by upregulating another factor that represses Aqp5. In either case, upregulation of Aqp5 may contribute to the polyuria phenotype in *Dot1l^AC^* mice and in DN patients.

Aqp5 promotes outflow of water in the lacrimal and salivary glands. Since Aqp5-mediated water transport in secretory epithelia occurs in the secretory direction in response to an osmotic gradient, water transport via Aqp5 in kidney would also move down an osmotic gradient. Thus, like Aqp2, Aqp5 would be expected to mediate water uptake. Obviously, the polyuria phenotype does not support this hypothesis. Identification of Aqp5 as a negative regulator of Aqp2 apical localization offers a clue for an opposite role.

Apical positioning of Aqp2 involves multiple complex pathways that can be constitutive or cAMP- and cGMP-dependent. Protein-protein interactions play an important role in these pathways. Several Aqp2-binding proteins have been identified [42]–[49]. The physiological and pathophysiological significance of these interactions is largely unknown.

In contrast, the importance of the interaction between WT AQP2 and some AQP2 mutants is clearly demonstrated in autosomal dominant nephrogenic diabetes insipidus (NDI). All dominant mutations identified so far occur within the C-terminus of AQP2. The AQP2 mutants form heterotetramers with WT AQP2 and thus prevent WT AQP2 from reaching the plasma membrane. The WT AQP2 is retained either in the Golgi, late endosomes, lysosomes, or the basolateral plasma membrane [50]. In several aspects, AQP5 seems to behave like the AQP2 mutants. First, the high sequence homology with 66% amino acid identity offers a potential molecular basis for AQP2-AQP5 interaction. Such interaction may interfere with their homotetramer formation and facilitate hybrid arrangement. Secondly, the C-termini of AQP2 and AQP5 are important for trafficking, but not for tetramer formation [51]. They are not conserved between the two proteins, mimicking the difference between the WT and mutant AQP2 forms.

Our finding that AQP5 is readily detectable and colocalizes with AQP2 at the perinuclear region suggests that the apical localization of AQP2 may be impaired and contributory to the deterioration of glucosuria-induced polyuria in patients with DN. Earlier studies show that compensatory Aqp2 protein abundance increases in rats with streptozotocin-induced diabetic mellitus [52]–[54], which apparently differs from the present results. All of these animals studies were conducted in a relatively short period ranging from day 5 to day 21 following STZ treatment [52]–[54]. While diabetic mellitus was clearly induced, no pathological data were shown in any of these studies to demonstrate tubular abnormalities as seen in the patients with DN, raising the possibility that renal pathological changes in these diabetic rats, particular in terms of tubular aberration, are not comparable with those in the patients with DN. It is likely that the increased Aqp2 expression is a result of compensatory mechanism in the early stages of the diseases progression. Such a compensatory mechanism may be lost in the advanced stage of DN development.

As an aldosterone downregulated gene [24], [27], [28], *Dot1l* may also links excessive aldosterone to polyuria via Aqp5 and Aqp2. Long-term aldosterone infusion decreases Aqp2 apical localization and causes polyuria in several animal models [36], [55], [56]. It is worthy to determine if Dot1a is really downregulated and Aqp5 upregulated in these experimental conditions.

Although our data strongly support the formation and localization of AQP2/AQP5 complex in the ER/Golgi as the potential underlying mechanism by which Aqp5 plays an inhibitory role in Aqp2 membrane localization and thus in the development of polyuria, conclusive demonstration requires blocking Aqp5 in *Dot1l^AC^* mice. Genetic inactivation using *Aqp5^−/−^* mice, pharmacological blocking with Aqp5-specific inhibitors, and siRNA-mediated in vivo depletion of Aqp5 offer three different strategies. To our knowledge, there are currently no Aqp5-specific inhibitors. Since extensive filtration and uptake of siRNA take place in the proximal tubule cells, but not in the distal tubule cells [57], where *Dot1l*-deletion-dependent Aqp5 expression occurs, siRNA-mediated in vivo knockdown of Aqp5 may not be effective.

## Materials and Methods

### Ethics Statement

All animal studies were performed in accordance with NIH Guides for the Care and Use of Laboratory Animals and were approved by the University of the University of Texas Health Science Center at Houston Animal Welfare Committee. For kidney biopsy studies, approval of the protocol (11-0144) was obtained from the University of Texas Health Science Center at Houston Institutional Review Board. No participants were specifically recruited for this project to collect the kidney biopsy samples. Kidney archival biopsy residual specimens from patients with the diagnosis of DN and patients with MCD were exclusively used. The Institutional Review Board determined that this project is qualified for exempt status according to 45 CFR 46.101(b), CATEGORY #4: Research, involving the collection or study of existing data, documents, records, pathological specimens, or diagnostic specimens, if these sources are publicly available or if the information is recorded by the investigator in such a manner that subjects cannot be identified directly or through identifiers linked to the subjects. Accordingly, the written or verbal informed consent of the “participants” to participate in this study was waived. The Department of Pathology and Laboratory Medicine, University of Texas Medical School at Houston provided the PI with these archival kidney biopsy samples as unidentified materials. MCDs without obvious tubular abnormalities were selected and used to serve as “normal” controls.

### Reagents

Primary antibodies used were rabbit antibodies for H3 di-methyl K79 (ab3594, abcam), goat anti-Aqp2 (sc-9882, Santa Cruz), rabbit anti-Aqp5 (A4979, Sigma), rabbit anti-Dot1l (A300-953A, Bethyl), mouse anti-GM130 (610822, BD Sciences), and chicken anti-Aqp2 LC54 (from Dr. James B. Wade, Univ of Maryland). DsRed-ER and GFP-Dot1a have been described [17], [39]. Kits for cell surface protein isolation and chromatin immunopreciptation were purchased from Pierce and Millipore, respectively. All of the Aqp5 promoter-luciferase constructs and MLE-15 cells have been detailed in our previous work [38]. Human AQP2 and AQP5 were amplified with EST clones BC042496 and BC032946, respectively, cloned into various vectors at EcoRI/XhoI to generate FLAG-, Myc-, GFP-, and RFP-tagged fusions. Sequence authenticity of each construct was verified by sequencing.

### STZ treatment

Two-month-old male Dot1l^f/f^ and Dot1l^AC^ mice received 5 consecutive daily intraperitoneal injections of STZ-Na-Citrate solution (50 mg/kg), according to the Low-Dose Streptozotocin Induction Protocol (Mouse) established by Animal Models of Diabetic Complications Consortium (http://www.diacomp.org/shared/protocols.aspx?model=9). Blood glucose was monitored with a glucometer, using a one-drop blood sample collected through the tip of the tail. Once the blood glucose level reaches >200 mg/dl, mice were considered as diabetic and maintained for additional three months. Detailed characterization of diabetic *Dot1l^f/f^* and *Dot1l^AC^* mice will be reported elsewhere.

### Metabolic balance studies

All experimental mice were 2–5 months old, and sex- and age-matched. After acclimation for 3–7 days to Tecniplast metabolic cages (Exton) with free access to water and normal Na^+^ (0.4%) diet (fed state), mice were subsequently subject to water deprivation for 24 h. Twenty-four-hour urine samples were collected and analyzed as we reported before [36]. In brief, twenty-four-hour urine in fed state was collected daily for at least three consequtive days. For each mouse, the urine data from multiple days were pooled to calculate the final average to represent that mouse and counted as 1 (n = 1). To minimize circadian effects, urine collection were conducted around 5:00 p.m. each day. For water deripvation studies, water deprivation and urine collection were conducted samultaneiously for 24 hours. For STZ-induced diabetic mice, twenty-four-hour urine collection was conducted in three consequtive days at the end of the experiment (three months after blood glucose reached >200 mg/dl). To ensure complete and accurate urine collection, bladder voiding was conducted before the collection started and urine discarded. At the end of collection time point, bladder voiding was repeated and the urine collected. To minimize vaporization and thus urine volume loss, a fixed amount of corn oil was added to each of the containers so that the collected urine samples were covered by the oil during the collection period.

### Urine measurements

Urinary [Na^+^], [K^+^], and [creatinine] were measured with an analyzer (Roche Cobas Integra 400 plus) in the Clinical Pathology Laboratory, Department of Veterinary Medicine and Surgery, University of Texas MD Anderson Cancer Center as described [36]. Urine osmolarity was determined by vapor pressure (Wescor Vapro Vapor Pressure Osmometer 5520, Scimetrics, Houston, TX, USA) [36].

### Microarray analysis

Microarray experiments were carried out using the dual-color Agilent 4X44K “Whole Mouse Genome Array” system (Agilent) and the manufacturer's protocol followed. Detailed protocol can be found at Agilent website: www.Agilent.com. Briefly, total kidney RNA of *Dot1l^f/f^* and *Dot1l^AC^* mice (n = 4 mice/genotype) was isolated using Trizol (Invitrogen). Equal amounts of total RNA from each mouse of the same genotype were mixed to represent the corresponding genotype. 200 ng of the mixed RNAs was used and labeled with either Cy3- or Cy5-CTP. After 17 hr hybridization at 65°C, the arrays were washed and scanned with Agilent's dual-laser based scanner. Then, Feature Extraction software GE2-v5_95 was used to link a feature to a design file and determine the relative fluorescence intensity between the two samples. Genelists were created using pValue information from the internal replicates within the microarray. Data were deposited to Gene Expression Omnibus, with access number GSE40090.

### Immunofluorescence and confocal staining

Mouse kidney tissues were fixed in 4% paraformaldehyde overnight at 4°C and embedded in paraffin. After boiling in antigen-retrieval buffer, paraffin sections were blocked with 5% BSA/0.5% Triton X-100 in PBS. Each primary antibody was diluted in 5% BSA/0.5% Triton X-100 in PBS and incubated overnight at 4°C. Following four 5-minute washes in PBS, the sections were incubated with Dylight 594-AffiniPure goat anti-chicken IgG (Jackson ImmunoResearch LABORATORIES, Inc.), Alexa Fluor 488–conjugated goat anti-rabbit IgG or Alexa 594-conjugated goat anti-mouse IgG (Invitrogen), depending on the species from which the primary antibody was generated. Nuclei were visualized using DAPI (1∶1000). The sections were mounted in VECTASHIELD HardSet Mounting Medium (H-1400, VECTOR LABROTORIES), and examined under an epifluorescence microscope (Olympus IX71) and confocal microscope (510 Meta, Zeiss LSM) as previously described [26], [58].

For in vitro experiments, transfected IMCD3 cells with the plasmids as indicated in figure legends were fixed with 4% fresh prepared paraformaldehyde for 20 min, washed with PBS 3×5 min, and incubated in permealization buffer (0.1% Triton ×100, 5% Glycine in PBS) for 15 min. After 3×5 min washing with PBS, cells were blocked with Serum-free Protein Block (Dako) for 1 hr. Incubation of the primary and secondary antibodies, DAPI staining, mounting and microscopic examination were conducted as described above.

### Quantification of pixel intensity of AQP5 and H3m2K79 immunofluorescence staining in patients with DN

Kidney biopsies were immunostained under identical conditions. All digital images were acquired using the same settings including the same exposure time and amplification. Tubules with at least one AQP5^+^ cell were included for this analysis. All other AQP5^−^ tubules were excluded from the analyses. Among AQP5^+^ tubules, only AQP5^−^H3m2K79^+^, AQP5^+^H3m2K79^−^, and AQP5^+^H3m2K79^+^ cells were considered. AQP5^−^ H3m2K79^−^ cells were excluded as we already pointed out that loss of H3m2K79 might not always be associated with AQP5 expression. Spots of positive AQP5 staining without a clear DAPI-stained nucleus were excluded due to uncertainty of their H3m2K79 status. Under these restricted criteria, the pixel intensities over background of both AQP5 and H3m2K79 staining in the selected cells were quantified using ImageJ64 software.

### Cell surface biotinylation assay

This assay was done using the cell surface protein isolation kit (Pierce), following the manufacturer's instructions. Briefly, transiently transfected IMCD3 cells were biotinylated with Sulfo-NHS-SS-Biotin in cold PBS at 4°C for 30 min and the reaction stopped by addition of Quenching solution. Cells were washed with cold TBS and lysed with Lysis buffer containing proteinase inhibitor cocktail at 4°C for 30 min. Cell lysates were centrifuged for 2 min at 10,000 g. Supernatants were incubated with NeutrAvitin Agarose for 60 min at room temperature. Beads were washed four times with Wash buffer. Bound proteins were eluted by heating for 5 min at 95°C in 2× SDS-PAGE loading buffer (2% SDS, 0.0625 M Tris pH 6.8, 20% glycerol, 0.01% bromophenol blue, 5% β-mercaptoethanol). Samples were analyzed by immunoblotting with antibodies as indicated in the figure legends.

### Immunoblotting, luciferase assay, real-time RT-qPCR, and ChIP assay

These assays were conducted according to our published protocols [24], [25], [28], [36], For ChIP, a modified protocol described in our recent publication [33] was utilized. The sequences of the primers were listed in Table S3 and S4.

### Statistical analysis

Quantitative data are presented as mean±SEM. Student's t-test was used with the statistical significance set at P<0.05.

## Supporting Information

Figure S1 **Additional urine metabolic analyses of** ***Dot1l^AC^*** **and control mice after water deprivation for 24 h.** *Dot1l^f/f^* (f/f) and *Dot1l^AC^* (AC) mice were fed the normal Na^+^ diet (0.4% Na^+^) in metabolic cages, deprived of water for 24 h, and analyzed for the parameters as indicated. n = 14 mice/group. *P<0.05 vs. *Dot1l^f/f^*.
(DOC)

Figure S2 **Absolute urine volumes.** *Dot1l^f/f^* (f/f) and *Dot1l^AC^* (AC) mice with free access to water and regular diet (A), after 24-hr water deprivation (B), and after blood glucose reaching 200 mg/dl induced by STZ injection (C) were analyzed for the absolute urine volume. In each case, n = 4–14 mice/genotype. *P<0.05 vs. *Dot1l^f/f^*.
(DOC)

Figure S3 **Additional IF images showing that Aqp5 was significantly upregulated in the kidney of** ***Dot1l^AC^*** **mice on the normal Na^+^ pellet diet.** (**A–B**) Representative IF images showing Aqp5 (green) expression in Aqp2^+^ (red) cells in mice as indicated. Note: Some cells displayed Aqp5^+^ Aqp2^−^ phenotype. These cells are most likely the intercalated cells derived from the Aqp2-expressing progenitor cells or mature PC [30]. OM and IM: outer and inner medulla. Detection of Aqp5^+^ Aqp2^−^ and Aqp5^−^ Aqp2^+^ cells demonstrates the specificity of the two antibodies. Scale bar: 50 µM.
(DOC)

Figure S4 **Aqp5 is expressed in the developing** ***Dot1l^AC^*** **kidneys.** Representative IF images showing detectable Aqp5 in some Aqp2^+^ and Aqp2^−^ connecting tube/collecting duct cells of *Dot1l^AC^* mice at day 11 and day 20, but not at day 3. Aqp5 is not detectable in *Dot1l^f/f^* mice at all stages as indicated. Scale bar: 100 µM.
(DOC)

Table S1 Upregulated Genes in *Dot1l^AC^* vs. *Dot1l^f/f^* mice. Total kidney RNA of *Dot1l^f/f^* and *Dot1l^AC^* mice (n = 4 mice/genotype) was subjected to microarray analyses. There were 1359 genes represented by 1423 unique probes. These genes were upregulated with at least ≥2-fold higher mRNA levels in *Dot1l^AC^* vs. *Dot1l^f/f^* mice.
(DOC)

Table S2 Downregulated genes in *Dot1l^AC^* vs. *Dot1l^f/f^* mice. As in Table S1, total kidney RNA of *Dot1l^f/f^* and *Dot1l^AC^* mice (n = 4 mice/genotype) was subjected to microarray analyses. There 627 genes represented by 680 unique probes. These genes were downregulated with at least ≥2-fold lower mRNA levels in *Dot1l^AC^* vs. *Dot1l^f/f^* mice.
(DOC)

Table S3 Primers for real-time RT-qPCR and Dot1a-specific siRNA. Listed are sequences of the primers used for real-time RT-qPCR and Dot1a-specific siRNA. F: Forward. R: Reverse.
(DOC)

Table S4 Primers for ChIP in Aqp5 5′ flanking region. Listed are sequences of the primers in Aqp5 5′ flanking region. These primers were used in chromatin immunoprecipitation coupled with real-time qPCR analyses. F: Forward. R: Reverse.
(DOC)
