# Supplementary material for: Aqp5 Is a New Transcriptional Target of Dot1a and a Regulator of Aqp2
Source: PLoS One. 2013 Jan 10;8(1):e53342. doi: 10.1371/journal.pone.0053342 (PMC3542343; doi:10.1371/journal.pone.0053342)
Supplement: Figure S1 — Additional urine metabolic analyses of Dot1lAC and control mice after water deprivation for 24 h. Dot1lf/f (f/f) and Dot1lAC (AC) mice were fed the normal Na+ diet (0.4% Na+) in metabolic cages, deprived of water for 24 h, and analyzed for the parameters as indicated. n = 14 mice/group. *P<0.05 vs. Dot1lf/f. (DOC) [file pone.0053342.s001.doc]

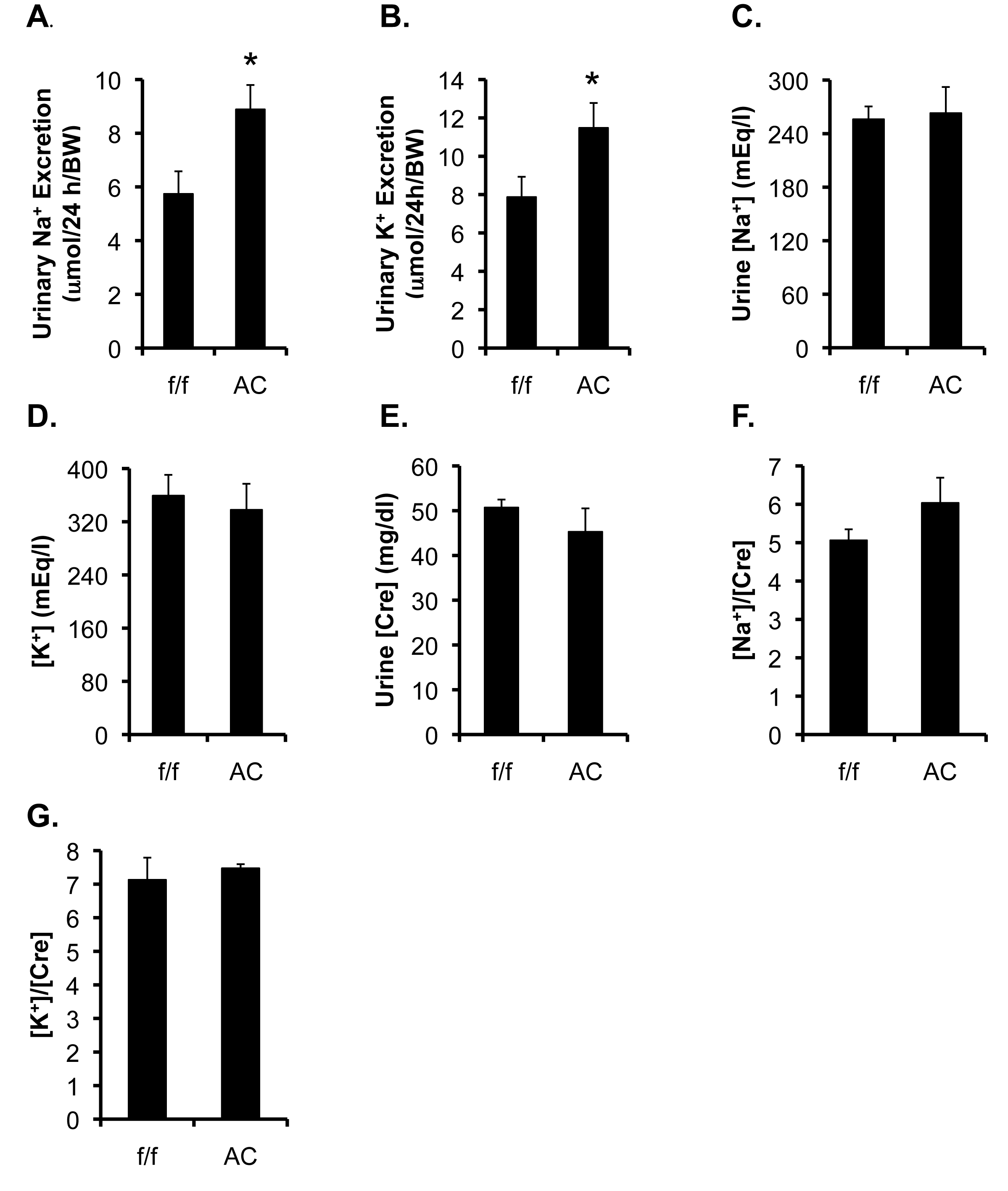


**Figure S1. Additional urine metabolic analyses of *Dot1l^AC^* and control mice after water deprivation for 24 h.** *Dot1l^f/f^* (f/f) and *Dot1l^AC^* (AC) mice were fed the normal Na^+^ diet (0.4% Na^+^) in metabolic cages, deprived of water for 24 h, and analyzed for the parameters as indicated. n= 14 mice/group. *P < 0.05 vs. *Dot1l^f/f^*.
