# Supplementary material for: Aqp5 Is a New Transcriptional Target of Dot1a and a Regulator of Aqp2
Source: PLoS One. 2013 Jan 10;8(1):e53342. doi: 10.1371/journal.pone.0053342 (PMC3542343; doi:10.1371/journal.pone.0053342)
Supplement: Figure S2 — Absolute urine volumes. Dot1lf/f (f/f) and Dot1lAC (AC) mice with free access to water and regular diet (A), after 24-hr water deprivation (B), and after blood glucose reaching 200 mg/dl induced by STZ injection (C) were analyzed for the absolute urine volume. In each case, n = 4–14 mice/genotype. *P<0.05 vs. Dot1lf/f. (DOC) [file pone.0053342.s002.doc]

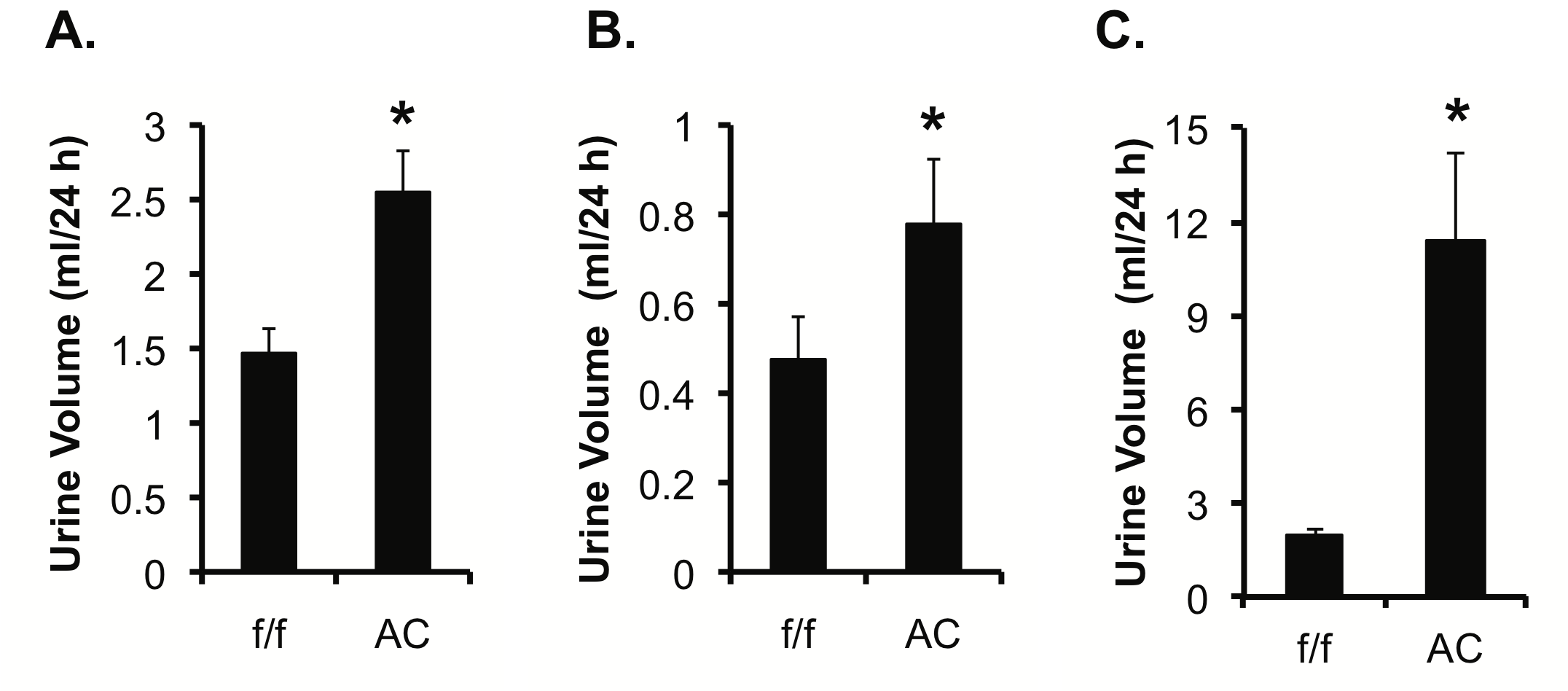


**Figure S2. Absolute urine volumes.** *Dot1l^f/f^* (f/f) and *Dot1l^AC^* (AC) mice with free access to water and regular diet (A), after 24-hr water deprivation (B), and after blood glucose reaching 200 mg/dl induced by STZ injection (C) were analyzed for the absolute urine volume. In each case, n= 4-14 mice/genotype. *P < 0.05 vs. *Dot1l^f/f^*.
