# Supplementary material for: Aqp5 Is a New Transcriptional Target of Dot1a and a Regulator of Aqp2
Source: PLoS One. 2013 Jan 10;8(1):e53342. doi: 10.1371/journal.pone.0053342 (PMC3542343; doi:10.1371/journal.pone.0053342)
Supplement: Figure S3 — Additional IF images showing that Aqp5 was significantly upregulated in the kidney of Dot1lAC mice on the normal Na+ pellet diet. (A–B) Representative IF images showing Aqp5 (green) expression in Aqp2+ (red) cells in mice as indicated. Note: Some cells displayed Aqp5+ Aqp2− phenotype. These cells are most likely the intercalated cells derived from the Aqp2-expressing progenitor cells or mature PC [30]. OM and IM: outer and inner medulla. Detection of Aqp5+ Aqp2− and Aqp5− Aqp2+ cells demonstrates the specificity of the two antibodies. Scale bar: 50 µM. (DOC) [file pone.0053342.s003.doc]

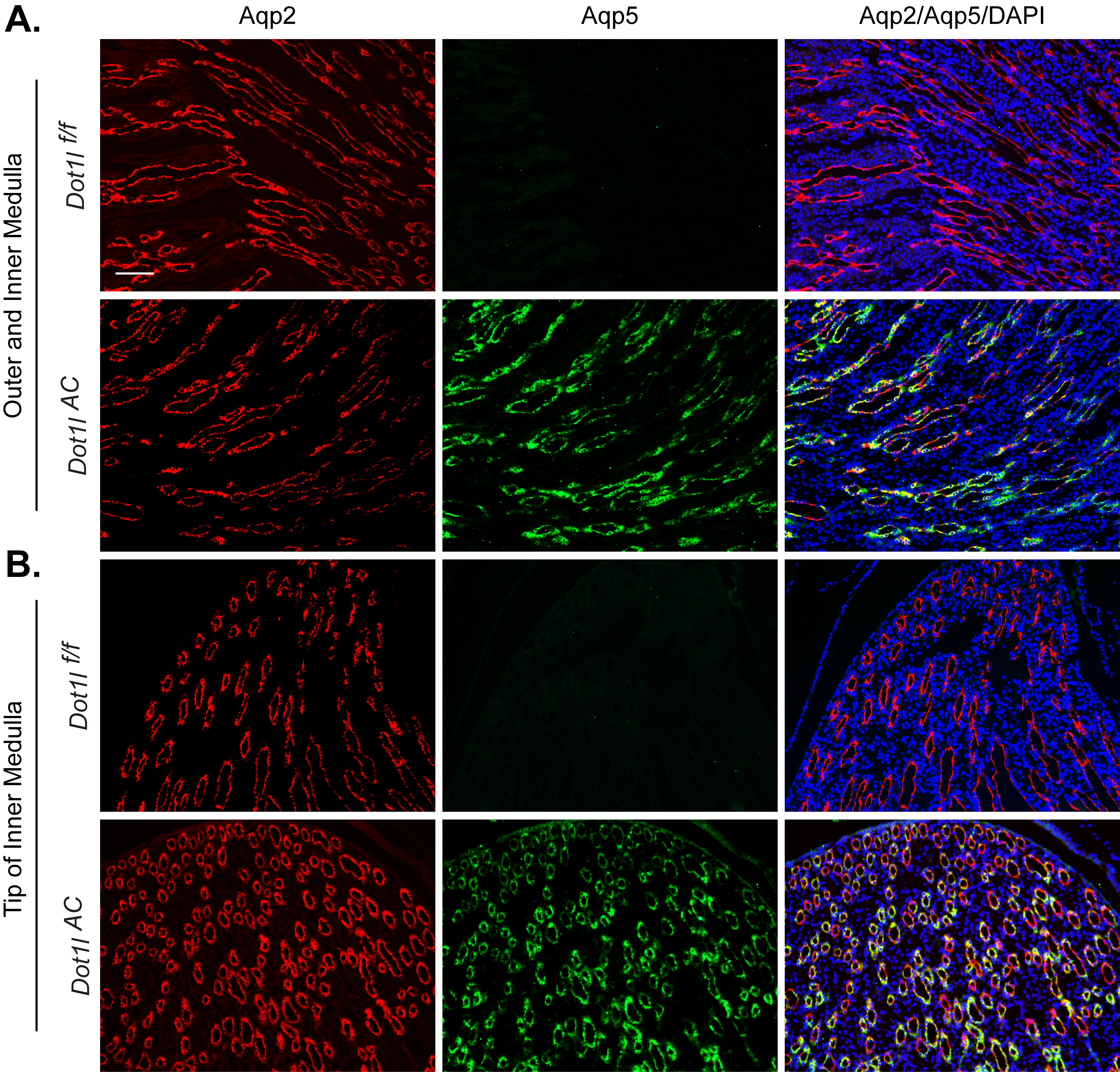


**Figure S3. Additional IF images showing that** **Aqp5 was significantly upregulated in the kidney of *Dot1l^AC^* mice on the normal Na^+^ pellet diet. (A-B)** Representative IF images showing Aqp5 (green) expression in Aqp2^+^ (red) cells in mice as indicated. Note: Some cells displayed Aqp5^+^ Aqp2^-^ phenotype. These cells are most likely the intercalated cells derived from the Aqp2-expressing progenitor cells or mature PC [30]. OM and IM: outer and inner medulla. Detection of Aqp5^+^ Aqp2^-^ and Aqp5^-^ Aqp2^+^ cells demonstrates the specificity of the two antibodies. Scale bar: 50 μM.
