# Supplementary material for: Aqp5 Is a New Transcriptional Target of Dot1a and a Regulator of Aqp2
Source: PLoS One. 2013 Jan 10;8(1):e53342. doi: 10.1371/journal.pone.0053342 (PMC3542343; doi:10.1371/journal.pone.0053342)
Supplement: Figure S4 — Aqp5 is expressed in the developing Dot1lAC kidneys. Representative IF images showing detectable Aqp5 in some Aqp2+ and Aqp2− connecting tube/collecting duct cells of Dot1lAC mice at day 11 and day 20, but not at day 3. Aqp5 is not detectable in Dot1lf/f mice at all stages as indicated. Scale bar: 100 µM. (DOC) [file pone.0053342.s004.doc]

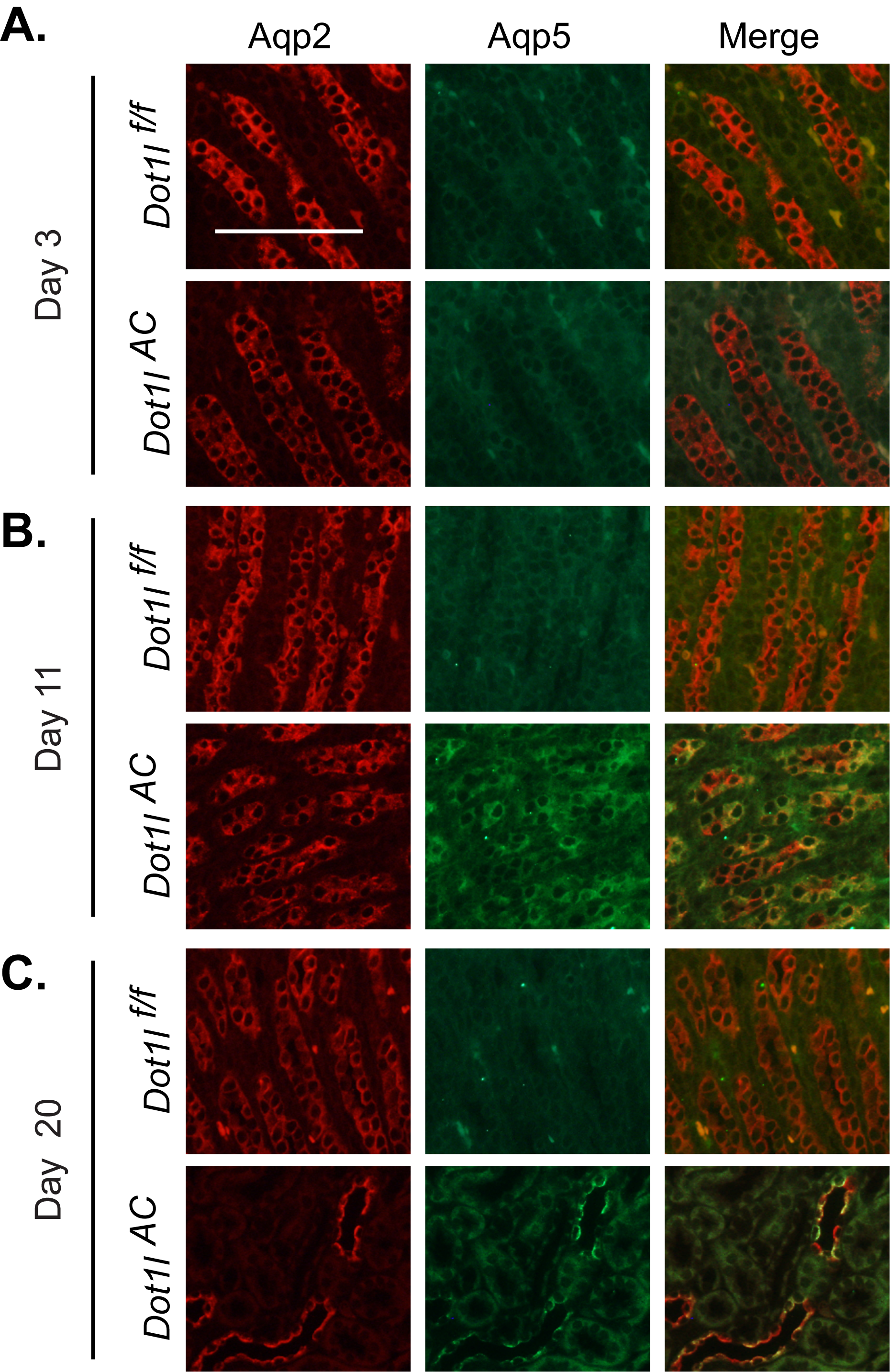


**Figure S4. Aqp5 is expressed in the developing *Dot1l^AC^* kidneys.** Representative IF images showing detectable Aqp5 in some Aqp2^+^ and Aqp2^-^ connecting tube/collecting duct cells of *Dot1l^AC^* mice at day 11 and day 20, but not at day 3. Aqp5 is not detectable in *Dot1l^f/f^* mice at all stages as indicated. Scale bar: 100 μM.
