# Supplementary material for: Aqp5 Is a New Transcriptional Target of Dot1a and a Regulator of Aqp2
Source: PLoS One. 2013 Jan 10;8(1):e53342. doi: 10.1371/journal.pone.0053342 (PMC3542343; doi:10.1371/journal.pone.0053342)
Supplement: Table S1 — Upregulated Genes in Dot1lAC vs. Dot1lf/f mice. Total kidney RNA of Dot1lf/f and Dot1lAC mice (n = 4 mice/genotype) was subjected to microarray analyses. There were 1359 genes represented by 1423 unique probes. These genes were upregulated with at least ≥2-fold higher mRNA levels in Dot1lAC vs. Dot1lf/f mice. (DOC) [file pone.0053342.s005.doc]

**Table S1. Upregulated Genes in *Dot1l^AC^* vs. *Dot1l^f/f^*mice.** Total kidney RNA of *Dot1l^f/f^* and *Dot1l^AC^* mice (n=4 mice/genotype) was subjected to microarray analyses. There were 1359 genes represented by 1423 unique probes. These genes were upregulated with at least ≥ 2-fold higher mRNA levels in *Dot1l^AC^* vs. *Dot1l^f/f^* mice.

| **Probe ID** | **Gene ID** | **Fold change**  **(*Dot1l^AC^* vs. *Dot1l^f/f^*)** |
| --- | --- | --- |
| A_51_P207601 | Aqp5 | 26.03 |
| A_51_P461779 | Ppp2r2c | 15.36 |
| A_52_P60194 | C4bp | 11.10 |
| A_51_P339934 | Nefl | 9.63 |
| A_51_P104258 | Olfr1154 | 8.49 |
| A_52_P172131 | B230343J05Rik | 8.01 |
| A_52_P1028855 | AK085732 | 7.91 |
| A_51_P278653 | Rprm | 7.84 |
| A_51_P235619 | AK041360 | 7.46 |
| A_51_P378880 | AK082259 | 7.44 |
| A_51_P346243 | Gp1ba | 7.41 |
| A_52_P559044 | Mapk8 | 7.26 |
| A_51_P370552 | 9230117E20Rik | 6.85 |
| A_52_P146865 | Pcdhb11 | 6.76 |
| A_51_P241387 | Barx2 | 6.75 |
| A_51_P351166 | Myod1 | 6.73 |
| A_51_P283323 | Olfr1247 | 6.68 |
| A_52_P875817 | AK030228 | 6.68 |
| A_51_P338295 | AK036163 | 6.67 |
| A_51_P419226 | S100a14 | 6.63 |
| A_51_P232416 | Rag1 | 6.60 |
| A_52_P510169 | Sh3gl2 | 6.59 |
| A_51_P361443 | 4930534B04Rik | 6.57 |
| A_52_P786757 | AK045569 | 6.54 |
| A_52_P1148772 | AK089526 | 6.53 |
| A_51_P379069 | Dkk1 | 6.52 |
| A_52_P5905 | 4933407L21Rik | 6.51 |
| A_52_P867819 | AK039920 | 6.42 |
| A_52_P1172407 | AK042025 | 6.42 |
| A_51_P294233 | Nanog | 6.39 |
| A_52_P655925 | Sfmbt2 | 6.37 |
| A_52_P842899 | D530037P16Rik | 6.10 |
| A_51_P385099 | Tnf | 6.04 |
| A_51_P484698 | Aipl1 | 6.03 |
| A_51_P312052 | AK053398 | 5.98 |
| A_52_P376119 | AK050562 | 5.97 |
| A_52_P171166 | BC048679 | 5.96 |
| A_52_P588594 | Zfp386 | 5.94 |
| A_51_P127346 | Hsfy2 | 5.86 |
| A_51_P504735 | Olfr1248 | 5.83 |
| A_52_P562082 | NAP044381-1 | 5.79 |
| A_52_P562676 | Sult4a1 | 5.76 |
| A_51_P234503 | Hyal5 | 5.75 |
| A_52_P337246 | Isl1 | 5.71 |
| A_51_P110329 | Vsig1 | 5.68 |
| A_52_P1075681 | AK033103 | 5.68 |
| A_52_P1028477 | AK080127 | 5.62 |
| A_52_P722518 | 4930519D14Rik | 5.59 |
| A_51_P518228 | 1700018L24Rik | 5.56 |
| A_52_P321857 | ENSMUST00000099847 | 5.56 |
| A_52_P515857 | Reln | 5.52 |
| A_51_P198664 | Mrgpra4 | 5.46 |
| A_52_P707673 | AK138093 | 5.45 |
| A_51_P308697 | Olfr164 | 5.41 |
| A_52_P1043586 | AK032987 | 5.41 |
| A_52_P1172018 | AK038131 | 5.40 |
| A_52_P980581 | AK089955 | 5.39 |
| A_52_P1036629 | AK076723 | 5.38 |
| A_51_P473024 | Nrp2 | 5.37 |
| A_51_P215729 | 9530053A07Rik | 5.36 |
| A_52_P771700 | AK079798 | 5.34 |
| A_51_P444920 | Olfr22-ps1 | 5.34 |
| A_51_P510900 | Serpini2 | 5.33 |
| A_52_P98348 | TC1475345 | 5.30 |
| A_52_P738593 | 4930522O17Rik | 5.27 |
| A_52_P810830 | LOC433426 | 5.26 |
| A_52_P26737 | Coch | 5.24 |
| A_51_P112796 | Il1f5 | 5.21 |
| A_52_P441294 | Chl1 | 5.19 |
| A_51_P169987 | AK080653 | 5.18 |
| A_51_P519460 | 4930524B15Rik | 5.17 |
| A_51_P464621 | A_51_P464621 | 5.14 |
| A_51_P234371 | Ror2 | 5.14 |
| A_52_P271495 | A430108G06Rik | 5.13 |
| A_52_P365316 | Hecw1 | 5.07 |
| A_52_P996686 | AK089848 | 5.00 |
| A_52_P553109 | AK042731 | 5.00 |
| A_51_P391955 | 2310032F03Rik | 4.99 |
| A_51_P121086 | V1ra2 | 4.99 |
| A_52_P891765 | BB148868 | 4.97 |
| A_51_P406974 | Olfr518 | 4.96 |
| A_52_P883166 | AK031406 | 4.95 |
| A_52_P92037 | Uox | 4.95 |
| A_52_P656628 | Crx | 4.93 |
| A_51_P190979 | Olfr1166 | 4.93 |
| A_51_P246119 | Cenpf | 4.92 |
| A_52_P875588 | AK047428 | 4.90 |
| A_51_P201672 | ENSMUST00000066614 | 4.90 |
| A_52_P423462 | NAP022882-001 | 4.88 |
| A_52_P301115 | D230046B21Rik | 4.81 |
| A_52_P557170 | Olfr996 | 4.79 |
| A_51_P350976 | Krtap6-2 | 4.77 |
| A_52_P361907 | Olfr1286 | 4.76 |
| A_51_P420541 | 5830457O10Rik | 4.74 |
| A_52_P987826 | AK034409 | 4.74 |
| A_51_P232128 | Pdcl2 | 4.72 |
| A_51_P351523 | Jph1 | 4.71 |
| A_52_P473266 | ENSMUST00000090634 | 4.68 |
| A_52_P747875 | AK089203 | 4.65 |
| A_52_P537314 | A930004K21Rik | 4.64 |
| A_51_P472153 | Rnase1 | 4.64 |
| A_51_P373629 | Chrnb4 | 4.63 |
| A_51_P259765 | 4930564B18Rik | 4.63 |
| A_52_P508827 | Lhx8 | 4.62 |
| A_52_P374075 | Csmd2 | 4.62 |
| A_51_P387388 | Olfr960 | 4.58 |
| A_52_P614860 | 6430701C03Rik | 4.58 |
| A_52_P609868 | Timd4 | 4.56 |
| A_51_P380056 | 4930432F04Rik | 4.55 |
| A_52_P78183 | Rnf43 | 4.54 |
| A_52_P121342 | Nrxn1 | 4.54 |
| A_52_P585181 | AK032998 | 4.53 |
| A_51_P518156 | 4930553D19Rik | 4.52 |
| A_52_P275700 | Nr2f1 | 4.52 |
| A_51_P443819 | 2610034M16Rik | 4.51 |
| A_51_P200582 | AK051391 | 4.50 |
| A_52_P80702 | 4930503B20Rik | 4.49 |
| A_51_P454782 | Serpinb7 | 4.47 |
| A_52_P535101 | TC1457252 | 4.45 |
| A_52_P72162 | Fsip2 | 4.44 |
| A_52_P212838 | D930043O14Rik | 4.43 |
| A_51_P251387 | ENSMUST00000006776 | 4.39 |
| A_51_P457237 | Xlr4b | 4.38 |
| A_51_P289002 | AK082671 | 4.36 |
| A_52_P55627 | Mrgpra2 | 4.34 |
| A_51_P295511 | Aanat | 4.33 |
| A_51_P493940 | 1700023D19Rik | 4.32 |
| A_52_P459521 | Itgb4 | 4.30 |
| A_51_P137836 | AK089449 | 4.30 |
| A_52_P11061 | Tomm70a | 4.28 |
| A_51_P400480 | AK080995 | 4.28 |
| A_52_P233927 | Fpgt | 4.27 |
| A_51_P144813 | Cdkn2a | 4.27 |
| A_51_P318510 | Ptprn | 4.25 |
| A_52_P1059369 | 1700037N05Rik | 4.22 |
| A_52_P592177 | Disc1 | 4.19 |
| A_51_P421158 | AK041880 | 4.18 |
| A_52_P339989 | Mafa | 4.17 |
| A_52_P227995 | Lyzl6 | 4.17 |
| A_51_P519008 | Mkx | 4.17 |
| A_51_P122821 | NAP057123-1 | 4.16 |
| A_52_P237997 | Negr1 | 4.15 |
| A_52_P212408 | C030002J06Rik | 4.15 |
| A_51_P134913 | Gpr15 | 4.15 |
| A_52_P49378 | Kif1a | 4.15 |
| A_52_P49378 | Kif1a | 4.15 |
| A_52_P279425 | Cd96 | 4.15 |
| A_52_P516962 | Olfr1519 | 4.11 |
| A_51_P133060 | ENSMUST00000032763 | 4.11 |
| A_52_P148196 | 8030475D13Rik | 4.10 |
| A_51_P233338 | Olfr46 | 4.09 |
| A_52_P303491 | Grid2 | 4.09 |
| A_51_P509109 | Olfr806 | 4.09 |
| A_51_P501260 | Hist1h1d | 4.08 |
| A_51_P138303 | LOC545136 | 4.08 |
| A_51_P366306 | Zcchc12 | 4.08 |
| A_52_P220743 | Dhdh | 4.08 |
| A_51_P117411 | 5730427N09Rik | 4.08 |
| A_52_P184395 | Actl6b | 4.08 |
| A_52_P225959 | Cngb3 | 4.07 |
| A_51_P515158 | ENSMUST00000062557 | 4.07 |
| A_52_P452199 | NAP050063-1 | 4.07 |
| A_52_P787975 | AK076568 | 4.06 |
| A_52_P1035635 | AK076371 | 4.06 |
| A_52_P330224 | 9530023I19Rik | 4.05 |
| A_51_P497280 | Dbndd1 | 4.05 |
| A_51_P313444 | Fkbp6 | 4.05 |
| A_52_P136026 | Olfr1199 | 4.04 |
| A_51_P331862 | Svs3 | 4.04 |
| A_52_P1051779 | AK038867 | 4.04 |
| A_52_P319115 | Kctd4 | 4.04 |
| A_51_P446395 | Csnb | 4.03 |
| A_52_P440034 | 4933428D01Rik | 4.03 |
| A_51_P185415 | Il5 | 4.02 |
| A_52_P1124491 | AK087372 | 4.01 |
| A_52_P684896 | Ptprd | 4.01 |
| A_51_P270874 | 4930469G21Rik | 4.00 |
| A_52_P811431 | AK079697 | 4.00 |
| A_52_P658070 | D030074E01Rik | 4.00 |
| A_51_P370372 | Olfr876 | 4.00 |
| A_51_P393454 | Ntrk2 | 3.98 |
| A_51_P330736 | AK049252 | 3.98 |
| A_52_P623036 | AK050427 | 3.97 |
| A_52_P1068401 | AK053316 | 3.97 |
| A_52_P618932 | Lcn9 | 3.97 |
| A_51_P285462 | Olfr1394 | 3.96 |
| A_52_P216427 | Titf1 | 3.96 |
| A_51_P191669 | Chgb | 3.96 |
| A_52_P316749 | AK088911 | 3.95 |
| A_51_P325664 | Olfr799 | 3.95 |
| A_51_P288719 | BC006965 | 3.94 |
| A_51_P431985 | Tyrp1 | 3.93 |
| A_51_P495131 | Prlpo | 3.93 |
| A_51_P307589 | Tdrd9 | 3.92 |
| A_51_P345995 | Krtap16-5 | 3.92 |
| A_51_P267913 | Btn2a2 | 3.91 |
| A_52_P127280 | Anubl1 | 3.90 |
| A_52_P1027988 | B130011D17Rik | 3.90 |
| A_51_P243103 | A630006J10Rik | 3.90 |
| A_52_P140287 | Spn | 3.89 |
| A_51_P112026 | D5Ertd798e | 3.88 |
| A_52_P277097 | Lrba | 3.88 |
| A_52_P415836 | 2310002F09Rik | 3.87 |
| A_51_P485014 | Hoxb1 | 3.87 |
| A_52_P25170 | E230016K23Rik | 3.87 |
| A_52_P1075940 | AK045080 | 3.86 |
| A_51_P219173 | Kcnmb1 | 3.86 |
| A_52_P670577 | NAP113739-1 | 3.86 |
| A_51_P389597 | Ins2 | 3.85 |
| A_52_P941235 | C78532 | 3.84 |
| A_52_P572554 | ENSMUST00000095369 | 3.83 |
| A_52_P659544 | Dnahc3 | 3.83 |
| A_52_P319954 | Syt15 | 3.83 |
| A_51_P433789 | E030025L21Rik | 3.83 |
| A_51_P416074 | Ryr1 | 3.83 |
| A_52_P127325 | AI182371 | 3.83 |
| A_52_P843185 | 2610020H08Rik | 3.83 |
| A_52_P359431 | 5730410E15Rik | 3.82 |
| A_51_P233939 | AK045650 | 3.82 |
| A_52_P478193 | Kdr | 3.82 |
| A_52_P811047 | AK043364 | 3.81 |
| A_51_P251964 | E330034G19Rik | 3.80 |
| A_52_P374053 | 6330417G02Rik | 3.79 |
| A_51_P150845 | Tcerg1l | 3.79 |
| A_51_P401573 | Defb2 | 3.78 |
| A_51_P345538 | 5730522E02Rik | 3.74 |
| A_52_P475630 | AK083637 | 3.74 |
| A_52_P469440 | 5730405A10Rik | 3.74 |
| A_52_P907897 | AK027952 | 3.73 |
| A_51_P163942 | Nos2 | 3.72 |
| A_52_P1044334 | AK048816 | 3.72 |
| A_52_P95096 | 1700025L06Rik | 3.70 |
| A_51_P241577 | Spats1 | 3.69 |
| A_51_P159641 | Olfr203 | 3.69 |
| A_52_P378287 | 4930431B11Rik | 3.69 |
| A_51_P416669 | Krt2-1 | 3.69 |
| A_52_P318281 | Pit1 | 3.67 |
| A_51_P279575 | Cdc25c | 3.67 |
| A_51_P441843 | BC025462 | 3.65 |
| A_52_P27564 | Wdr44 | 3.65 |
| A_51_P284503 | 1110032D16Rik | 3.65 |
| A_52_P818815 | AK029633 | 3.65 |
| A_52_P607212 | ENSMUST00000067916 | 3.65 |
| A_52_P128044 | Tmc3 | 3.65 |
| A_51_P486223 | Th | 3.64 |
| A_52_P679869 | Pcdh11x | 3.64 |
| A_51_P365037 | 6330412F12Rik | 3.64 |
| A_51_P211351 | Olfr677 | 3.64 |
| A_52_P524366 | LOC546994 | 3.63 |
| A_51_P267189 | Zfy2 | 3.63 |
| A_52_P714949 | AK041339 | 3.63 |
| A_51_P193975 | Kcng3 | 3.62 |
| A_51_P272407 | Mylk | 3.62 |
| A_51_P167668 | Myh3 | 3.62 |
| A_51_P352773 | Defcr-rs7 | 3.62 |
| A_51_P459288 | Smad9 | 3.62 |
| A_52_P842938 | AK029658 | 3.62 |
| A_51_P234440 | Catsper4 | 3.62 |
| A_52_P305876 | Hbb-bh1 | 3.61 |
| A_52_P96159 | Dsg2 | 3.61 |
| A_52_P32750 | Il7r | 3.61 |
| A_51_P353494 | Fetub | 3.61 |
| A_52_P996457 | AK087394 | 3.60 |
| A_52_P769867 | 1700029E06Rik | 3.59 |
| A_52_P249826 | Hhip | 3.58 |
| A_52_P546699 | Olfr1500 | 3.58 |
| A_52_P1092024 | AK049077 | 3.58 |
| A_52_P137550 | Shcbp1 | 3.58 |
| A_52_P527697 | Olfr934 | 3.58 |
| A_51_P221753 | Klk1b9 | 3.57 |
| A_52_P519620 | Olfr97 | 3.57 |
| A_51_P289017 | A230005M16Rik | 3.57 |
| A_52_P540342 | Sgip1 | 3.57 |
| A_52_P81120 | AK046345 | 3.57 |
| A_52_P739547 | AK084707 | 3.57 |
| A_52_P268911 | Zfpn1a2 | 3.56 |
| A_51_P453376 | Ros1 | 3.56 |
| A_51_P222773 | Foxa2 | 3.55 |
| A_52_P572476 | Dhrs9 | 3.55 |
| A_52_P253757 | Nalp1 | 3.55 |
| A_51_P354994 | Nrxn3 | 3.55 |
| A_51_P172889 | Shank1 | 3.55 |
| A_52_P142888 | Clec5a | 3.55 |
| A_52_P583989 | 1700017G21Rik | 3.54 |
| A_52_P890318 | 1700030L20Rik | 3.54 |
| A_51_P470981 | Fut10 | 3.54 |
| A_52_P1084348 | AK032303 | 3.52 |
| A_51_P465758 | 1200009O22Rik | 3.52 |
| A_51_P145948 | Neto1 | 3.52 |
| A_51_P361895 | AK039133 | 3.51 |
| A_52_P199922 | TC1438641 | 3.50 |
| A_52_P132792 | 2810442I21Rik | 3.50 |
| A_52_P600474 | Kif17 | 3.49 |
| A_51_P478581 | 1700030F18Rik | 3.49 |
| A_52_P1195522 | 4930465K12Rik | 3.49 |
| A_51_P298455 | C630035N08Rik | 3.48 |
| A_52_P481442 | 4732495E13Rik | 3.48 |
| A_52_P110534 | Ttk | 3.48 |
| A_51_P245503 | Ugt2b1 | 3.48 |
| A_52_P191086 | Kcnj6 | 3.47 |
| A_52_P18267 | Brca1 | 3.47 |
| A_51_P142350 | Acn9 | 3.47 |
| A_52_P518507 | Me3 | 3.47 |
| A_52_P329398 | Atp12a | 3.46 |
| A_52_P92772 | Abcb1b | 3.46 |
| A_52_P53144 | Gcnt3 | 3.46 |
| A_51_P380699 | Acsl6 | 3.45 |
| A_51_P364913 | AK084437 | 3.44 |
| A_52_P109232 | Olfr221 | 3.44 |
| A_52_P204311 | Spry4 | 3.44 |
| A_51_P483773 | 9330159N22Rik | 3.44 |
| A_51_P461123 | Tlr5 | 3.44 |
| A_51_P427619 | Tbx20 | 3.44 |
| A_52_P1044139 | AK048079 | 3.44 |
| A_51_P487062 | Olfr1344 | 3.44 |
| A_52_P415155 | Wnt6 | 3.44 |
| A_51_P114222 | Il2 | 3.43 |
| A_52_P46986 | Cryzl1 | 3.43 |
| A_51_P229992 | AK080994 | 3.42 |
| A_51_P419656 | Klk1b27 | 3.42 |
| A_52_P659270 | Man2a2 | 3.41 |
| A_51_P265695 | Gapdhs | 3.41 |
| A_51_P450248 | Esx1 | 3.41 |
| A_52_P494069 | Zdhhc2 | 3.40 |
| A_52_P1067575 | 4632411P08Rik | 3.40 |
| A_52_P638521 | 2010015L04Rik | 3.39 |
| A_52_P63637 | Olfr1155 | 3.39 |
| A_51_P238803 | BC033606 | 3.39 |
| A_51_P359822 | Sftpd | 3.39 |
| A_51_P498750 | ENSMUST00000099765 | 3.39 |
| A_51_P498750 | ENSMUST00000099765 | 3.39 |
| A_52_P504624 | Utrn | 3.39 |
| A_52_P940140 | AK085791 | 3.38 |
| A_52_P483409 | Prss7 | 3.37 |
| A_51_P518987 | 4930412F09Rik | 3.37 |
| A_52_P483799 | E430029J22Rik | 3.37 |
| A_51_P373573 | Cdh22 | 3.37 |
| A_51_P407915 | A530026G17 | 3.36 |
| A_51_P232921 | AK006831 | 3.36 |
| A_51_P471608 | Olfr1336 | 3.36 |
| A_52_P571715 | Troap | 3.35 |
| A_51_P194672 | AK042810 | 3.35 |
| A_52_P971306 | AK028988 | 3.34 |
| A_51_P178083 | Resp18 | 3.33 |
| A_52_P883511 | AK037270 | 3.33 |
| A_52_P148222 | C130076O07Rik | 3.33 |
| A_52_P443776 | ENSMUST00000100992 | 3.33 |
| A_51_P195044 | Dppa3 | 3.33 |
| A_52_P461976 | Blvra | 3.32 |
| A_52_P9464 | TC1525814 | 3.32 |
| A_51_P389244 | V1rc17 | 3.32 |
| A_52_P16615 | 5830417A05Rik | 3.32 |
| A_51_P274907 | 4930404L01Rik | 3.31 |
| A_51_P116211 | G630030J09Rik | 3.31 |
| A_52_P602987 | Prlr | 3.31 |
| A_52_P554650 | Gzmd | 3.30 |
| A_51_P444302 | AK039273 | 3.30 |
| A_52_P514200 | Defcr6 | 3.29 |
| A_51_P355702 | Trpv3 | 3.29 |
| A_52_P923903 | AK084562 | 3.28 |
| A_51_P344447 | Adam1b | 3.28 |
| A_51_P259930 | Apoa5 | 3.27 |
| A_52_P25251 | E130309F12Rik | 3.27 |
| A_52_P416367 | Asah2 | 3.27 |
| A_52_P723285 | AK027926 | 3.27 |
| A_51_P487137 | BB297715 | 3.26 |
| A_52_P867354 | AK037708 | 3.26 |
| A_51_P351306 | ENSMUST00000076729 | 3.26 |
| A_52_P343916 | A730008I21Rik | 3.25 |
| A_52_P219216 | F730015K02Rik | 3.25 |
| A_52_P241834 | AK033069 | 3.24 |
| A_51_P318999 | Cdx1 | 3.24 |
| A_51_P268595 | C530025M11Rik | 3.24 |
| A_52_P187488 | BC050811 | 3.24 |
| A_52_P180359 | 8030453O22Rik | 3.24 |
| A_52_P494730 | RP23-14F5.7 | 3.24 |
| A_52_P64570 | 4930435H24Rik | 3.23 |
| A_52_P684563 | 4932435O22Rik | 3.23 |
| A_51_P496674 | AK038427 | 3.23 |
| A_52_P17602 | NAP052469-1 | 3.23 |
| A_52_P387837 | NAP062641-1 | 3.23 |
| A_52_P283862 | Slc2a3 (Glut3) | 3.22 |
| A_51_P121962 | Lphn3 | 3.22 |
| A_52_P1140675 | AK087578 | 3.21 |
| A_52_P1035697 | AK028073 | 3.21 |
| A_52_P493965 | Gucy1a3 | 3.21 |
| A_51_P210970 | Wnt3a | 3.21 |
| A_51_P332003 | Aste1 | 3.21 |
| A_51_P214896 | AK037799 | 3.20 |
| A_52_P267736 | C530024P05Rik | 3.20 |
| A_51_P311566 | AK043660 | 3.20 |
| A_52_P139182 | TC1495093 | 3.20 |
| A_52_P634329 | LOC640530 | 3.20 |
| A_52_P699236 | AK040746 | 3.19 |
| A_52_P255478 | 6530401N04Rik | 3.18 |
| A_52_P455619 | Gipr | 3.18 |
| A_52_P187058 | Nptx2 | 3.18 |
| A_52_P485654 | ENSMUST00000070378 | 3.18 |
| A_52_P456335 | Myg1 | 3.18 |
| A_52_P188549 | Brcc3 | 3.18 |
| A_51_P435447 | Ppfia2 | 3.17 |
| A_52_P980501 | LOC667898 | 3.17 |
| A_52_P236288 | 1810053B23Rik | 3.17 |
| A_52_P356536 | NAP055769-1 | 3.17 |
| A_51_P323987 | B530002L08 | 3.16 |
| A_51_P162474 | Scml2 | 3.16 |
| A_52_P71447 | Zfp114 | 3.16 |
| A_52_P1084507 | BB658382 | 3.16 |
| A_52_P504719 | AK033818 | 3.16 |
| A_52_P424550 | C230014O12Rik | 3.16 |
| A_51_P327983 | 4833427G06Rik | 3.15 |
| A_52_P319066 | Rkhd3 | 3.15 |
| A_51_P293015 | Il25 | 3.15 |
| A_51_P241210 | Lhfpl3 | 3.15 |
| A_51_P469308 | AK089343 | 3.14 |
| A_52_P672636 | Olfr974 | 3.14 |
| A_52_P15532 | A430057O09 | 3.14 |
| A_52_P548377 | 4933422H20Rik | 3.14 |
| A_51_P503729 | Elavl4 | 3.13 |
| A_51_P316360 | Oxgr1 | 3.13 |
| A_52_P25932 | TC1522631 | 3.13 |
| A_52_P536025 | Rasgrf1 | 3.13 |
| A_52_P107639 | Moap1 | 3.13 |
| A_52_P986621 | 2900082C11Rik | 3.13 |
| A_51_P246487 | NAP057252-1 | 3.12 |
| A_52_P1092070 | AK045025 | 3.12 |
| A_52_P642136 | 2610209C05Rik | 3.12 |
| A_52_P900164 | AK083952 | 3.12 |
| A_52_P362061 | Rdh1 | 3.12 |
| A_52_P344152 | 1500004A08Rik | 3.12 |
| A_51_P426096 | Mmp7 | 3.11 |
| A_52_P660552 | Il7 | 3.11 |
| A_52_P681722 | 9330185C12Rik | 3.11 |
| A_51_P185781 | LOC666589 | 3.11 |
| A_52_P1084612 | AK086368 | 3.11 |
| A_51_P113322 | Nalp4a | 3.11 |
| A_52_P563765 | Wtap | 3.11 |
| A_52_P512274 | Dnhd3 | 3.11 |
| A_52_P507853 | Ikbkg | 3.10 |
| A_52_P859493 | LOC624549 | 3.10 |
| A_52_P449993 | 6330512M04Rik | 3.10 |
| A_52_P236354 | Sumo1 | 3.10 |
| A_51_P452890 | Ssxb1 | 3.10 |
| A_52_P567791 | Klhl13 | 3.10 |
| A_51_P415931 | 4831428F09Rik | 3.10 |
| A_51_P423199 | 1110032L06Rik | 3.09 |
| A_52_P386507 | Samd7 | 3.09 |
| A_51_P237668 | Bex2 | 3.09 |
| A_51_P121485 | Olfr355 | 3.09 |
| A_52_P1195461 | Hace1 | 3.09 |
| A_51_P160935 | Cacng8 | 3.08 |
| A_52_P1045123 | TC1474992 | 3.08 |
| A_51_P345626 | Tnni3k | 3.08 |
| A_51_P135148 | Slpi | 3.08 |
| A_51_P453235 | BC010584 | 3.08 |
| A_52_P407636 | X99384 | 3.08 |
| A_51_P224660 | AK046608 | 3.08 |
| A_51_P130115 | Spink2 | 3.08 |
| A_52_P1174 | Gal3st4 | 3.07 |
| A_52_P467221 | Apbb1ip | 3.07 |
| A_51_P155783 | Psg29 | 3.07 |
| A_52_P671965 | NAP030023-1 | 3.07 |
| A_51_P121756 | AK016846 | 3.06 |
| A_51_P302087 | Capza3 | 3.06 |
| A_52_P922893 | 9530013L04Rik | 3.05 |
| A_51_P364210 | Ms4a3 | 3.05 |
| A_51_P251092 | Birc1c | 3.05 |
| A_51_P179293 | 2310002L13Rik | 3.05 |
| A_52_P278630 | Six6os1 | 3.05 |
| A_51_P358303 | Olfr1351 | 3.05 |
| A_52_P343402 | Slc5a4b | 3.04 |
| A_52_P365948 | 5033406O09Rik | 3.04 |
| A_52_P286912 | NAP071160-1 | 3.04 |
| A_52_P818694 | D730047E02Rik | 3.04 |
| A_51_P395676 | A230091H23Rik | 3.03 |
| A_51_P406807 | Muc15 | 3.03 |
| A_51_P300867 | Map3k9 | 3.02 |
| A_51_P369330 | Olfr894 | 3.02 |
| A_52_P172910 | Serpinb12 | 3.02 |
| A_52_P656254 | 5430401F13Rik | 3.02 |
| A_52_P924359 | TC1485171 | 3.02 |
| A_52_P492550 | Ttc7 | 3.02 |
| A_51_P211854 | Selp | 3.02 |
| A_51_P187242 | Hrh2 | 3.02 |
| A_51_P116239 | Ptpn5 | 3.02 |
| A_51_P153132 | Kcne2 | 3.02 |
| A_52_P434662 | Ttyh1 | 3.01 |
| A_51_P400686 | AK085204 | 3.01 |
| A_51_P126365 | Gzmn | 3.01 |
| A_52_P350893 | Ank1 | 3.01 |
| A_52_P491253 | Wars2 | 3.01 |
| A_51_P350426 | AK018213 | 3.01 |
| A_51_P237512 | 1110001A07Rik | 3.01 |
| A_52_P638867 | Xpo5 | 3.01 |
| A_52_P669155 | Itgam | 3.01 |
| A_52_P630838 | Sec22a | 3.01 |
| A_51_P365091 | AK085649 | 3.00 |
| A_52_P121333 | Nhlrc2 | 3.00 |
| A_52_P365194 | Lsamp | 3.00 |
| A_52_P47461 | Tas2r102 | 3.00 |
| A_52_P77305 | Setd7 | 3.00 |
| A_52_P1084681 | AK078982 | 3.00 |
| A_51_P185161 | Olfr830 | 2.99 |
| A_52_P1020153 | AK050110 | 2.99 |
| A_52_P270135 | Grik2 | 2.99 |
| A_52_P517209 | LOC666484 | 2.99 |
| A_51_P217039 | Gdf3 | 2.99 |
| A_52_P63739 | Rya3 | 2.99 |
| A_52_P51786 | Sec1 | 2.99 |
| A_52_P407171 | 2210421G13Rik | 2.98 |
| A_52_P428345 | Adra2c | 2.98 |
| A_52_P899611 | AK047096 | 2.98 |
| A_52_P201920 | Tpbg | 2.98 |
| A_52_P691985 | BC018486 | 2.98 |
| A_52_P211889 | 1110051B16Rik | 2.98 |
| A_52_P1132648 | AK080985 | 2.98 |
| A_52_P365412 | AK035685 | 2.98 |
| A_52_P169964 | Cops7b | 2.98 |
| A_52_P876139 | Inpp1 | 2.97 |
| A_51_P461265 | AK039770 | 2.97 |
| A_52_P1078018 | ENSMUST00000049335 | 2.97 |
| A_51_P195598 | Olfr259 | 2.97 |
| A_52_P545158 | Sppl3 | 2.96 |
| A_52_P258617 | Cd180 | 2.96 |
| A_51_P344925 | AK038629 | 2.96 |
| A_51_P281950 | Olfr42 | 2.96 |
| A_52_P64735 | AK031157 | 2.95 |
| A_51_P317286 | 9430097D07Rik | 2.95 |
| A_51_P218535 | Neb | 2.95 |
| A_52_P109916 | Ryr3 | 2.95 |
| A_51_P459477 | Col11a1 | 2.94 |
| A_52_P747362 | D230044P21Rik | 2.93 |
| A_51_P442142 | 1700126L10Rik | 2.93 |
| A_51_P205334 | Tnfrsf8 | 2.93 |
| A_51_P341487 | 1700028P14Rik | 2.93 |
| A_51_P266977 | Znrf4 | 2.93 |
| A_52_P149017 | 4933426K21Rik | 2.93 |
| A_51_P171075 | Csf2 | 2.93 |
| A_52_P119007 | 1700041M19Rik | 2.92 |
| A_52_P174721 | 9230107O10Rik | 2.92 |
| A_52_P83704 | Myb | 2.91 |
| A_51_P357299 | Olfr211 | 2.91 |
| A_52_P586959 | A430090L17Rik | 2.91 |
| A_52_P537272 | Khdrbs2 | 2.91 |
| A_51_P501494 | V1rc7 | 2.91 |
| A_51_P250131 | LOC676015 | 2.91 |
| A_52_P166382 | 2810011L19Rik | 2.91 |
| A_52_P84567 | TC1507911 | 2.91 |
| A_52_P456296 | 4930432O21Rik | 2.91 |
| A_51_P194740 | V1rd9 | 2.91 |
| A_51_P213676 | AK051657 | 2.90 |
| A_52_P215343 | Ints8 | 2.90 |
| A_51_P122033 | Olfr154 | 2.90 |
| A_52_P955603 | AK046728 | 2.90 |
| A_52_P631884 | TC1472363 | 2.90 |
| A_52_P227536 | 2900083I11Rik | 2.90 |
| A_52_P51805 | AK034651 | 2.90 |
| A_51_P371500 | Atp8b3 | 2.90 |
| A_52_P225772 | 6330549D23Rik | 2.89 |
| A_51_P385981 | AK033839 | 2.89 |
| A_52_P241214 | NAP071547-1 | 2.89 |
| A_52_P282905 | LOC382044 | 2.89 |
| A_52_P1100524 | AK084052 | 2.89 |
| A_51_P122481 | 1700010L04Rik | 2.88 |
| A_52_P418692 | 4930449I04Rik | 2.88 |
| A_52_P427607 | Pglyrp3 | 2.88 |
| A_52_P266686 | Ntsr2 | 2.88 |
| A_52_P629112 | TC1413629 | 2.87 |
| A_51_P315027 | AF529169 | 2.87 |
| A_51_P405638 | LOC544905 | 2.87 |
| A_52_P1132199 | AK051555 | 2.87 |
| A_52_P439509 | Xlr5d | 2.87 |
| A_52_P133333 | Olfr197 | 2.87 |
| A_51_P250975 | AK046982 | 2.87 |
| A_52_P15139 | NAP106831-1 | 2.87 |
| A_52_P220370 | Ankrd41 | 2.87 |
| A_52_P989547 | A_52_P989547 | 2.86 |
| A_52_P34381 | Trpc2 | 2.86 |
| A_52_P810893 | AK040469 | 2.86 |
| A_51_P456234 | 2900006F19Rik | 2.86 |
| A_52_P204414 | AK043041 | 2.86 |
| A_51_P225634 | Zdhhc25 | 2.86 |
| A_52_P32727 | AK041470 | 2.86 |
| A_52_P470586 | ENSMUST00000037336 | 2.86 |
| A_52_P971745 | AK083098 | 2.85 |
| A_52_P631240 | Frmd4a | 2.85 |
| A_51_P305003 | Ntrk1 | 2.85 |
| A_51_P285229 | AK040234 | 2.85 |
| A_51_P510156 | Lcn2 | 2.85 |
| A_52_P842744 | 9130414P19Rik | 2.85 |
| A_51_P249889 | AK084210 | 2.85 |
| A_52_P148539 | Herc4 | 2.84 |
| A_51_P375526 | Pax2 | 2.84 |
| A_52_P584045 | Olfr855 | 2.84 |
| A_52_P341115 | 4930404I05Rik | 2.84 |
| A_51_P311523 | 9430099M06Rik | 2.84 |
| A_52_P43282 | Crisp2 | 2.84 |
| A_52_P492189 | TC1491311 | 2.84 |
| A_51_P404463 | 1500015O10Rik | 2.83 |
| A_51_P388819 | Hpcal4 | 2.83 |
| A_52_P143261 | C530030A11Rik | 2.83 |
| A_52_P365491 | 0710005M24Rik | 2.83 |
| A_52_P467488 | Slc35d3 | 2.83 |
| A_51_P489800 | 2810408A11Rik | 2.83 |
| A_52_P165575 | Olfr360 | 2.83 |
| A_52_P82636 | Tigd3 | 2.83 |
| A_51_P273979 | Cenpa | 2.82 |
| A_51_P197805 | Tesp2 | 2.82 |
| A_51_P354587 | 4933440G23Rik | 2.82 |
| A_52_P486964 | NAP051367-1 | 2.82 |
| A_52_P1179609 | Kcnh3 | 2.82 |
| A_51_P453569 | Olfr943 | 2.81 |
| A_52_P1076033 | AK037635 | 2.81 |
| A_52_P408499 | Ubap1 | 2.81 |
| A_52_P362360 | Xist | 2.81 |
| A_52_P577329 | A230069A22Rik | 2.81 |
| A_52_P486175 | Dusp27 | 2.81 |
| A_52_P533585 | NAP071486-1 | 2.81 |
| A_52_P554496 | Olfr1138 | 2.81 |
| A_52_P811929 | AK081324 | 2.81 |
| A_52_P746780 | AK035362 | 2.80 |
| A_52_P325789 | Coro2b | 2.80 |
| A_51_P461067 | Igh-4 | 2.80 |
| A_51_P122707 | Ldoc1 | 2.79 |
| A_51_P296397 | Olfr514 | 2.79 |
| A_51_P100379 | 6.72045E+21 | 2.79 |
| A_51_P433194 | Bcas1 | 2.79 |
| A_51_P408082 | Apoa1 | 2.79 |
| A_51_P405342 | AK043935 | 2.78 |
| A_52_P995908 | AK049930 | 2.78 |
| A_52_P590845 | Tmem20 | 2.78 |
| A_52_P924051 | AK084429 | 2.78 |
| A_52_P276348 | Adh6a | 2.78 |
| A_51_P407480 | Myt1 | 2.78 |
| A_52_P851691 | AK048971 | 2.77 |
| A_51_P315515 | Slc35f4 | 2.77 |
| A_52_P884135 | AK085881 | 2.77 |
| A_51_P430033 | Ccdc13 | 2.77 |
| A_51_P208340 | Olfr113 | 2.77 |
| A_52_P402677 | Lipl3 | 2.77 |
| A_52_P580582 | Nppa | 2.77 |
| A_52_P916262 | AK076312 | 2.77 |
| A_52_P262118 | Prdm15 | 2.77 |
| A_52_P616007 | NAP102121-1 | 2.77 |
| A_52_P313020 | 4932438A13Rik | 2.77 |
| A_52_P667324 | Syt5 | 2.77 |
| A_52_P203773 | D5Ertd579e | 2.77 |
| A_51_P264534 | 2410124H12Rik | 2.76 |
| A_51_P426150 | V1re3 | 2.76 |
| A_51_P156196 | Olfr604 | 2.76 |
| A_51_P169476 | Mcpt1 | 2.76 |
| A_51_P172514 | Sh2d1a | 2.76 |
| A_52_P466615 | Prkg2 | 2.76 |
| A_51_P233532 | 4930519F16Rik | 2.75 |
| A_52_P341128 | Ptprc | 2.75 |
| A_52_P218188 | Abcc8 | 2.75 |
| A_51_P403564 | Lhx5 | 2.75 |
| A_52_P216226 | Masp1 | 2.75 |
| A_51_P307082 | Hey2 | 2.75 |
| A_51_P369091 | Taf4b | 2.75 |
| A_52_P479334 | Olfr1370 | 2.75 |
| A_52_P504237 | Ceacam19 | 2.75 |
| A_52_P169696 | 9430076G02Rik | 2.75 |
| A_52_P436393 | Dmrt1 | 2.74 |
| A_52_P156314 | Esr2 | 2.74 |
| A_51_P131442 | Neud4 | 2.74 |
| A_52_P423222 | Nfe2l3 | 2.74 |
| A_52_P423222 | Nfe2l3 | 2.74 |
| A_51_P307166 | Il1rn | 2.74 |
| A_52_P524227 | Olfr149 | 2.74 |
| A_51_P162008 | Adamts12 | 2.74 |
| A_51_P106114 | A230070E04Rik | 2.73 |
| A_51_P398848 | Trim37 | 2.73 |
| A_51_P154867 | Pllp | 2.73 |
| A_51_P102311 | Tyr | 2.73 |
| A_52_P819979 | AK078658 | 2.73 |
| A_51_P238576 | Cyp4a14 | 2.73 |
| A_52_P366958 | A730020M07Rik | 2.73 |
| A_52_P159470 | Muc19 | 2.72 |
| A_51_P176448 | AK038757 | 2.72 |
| A_52_P963665 | AK049947 | 2.72 |
| A_52_P972159 | AK084173 | 2.72 |
| A_51_P428414 | ENSMUST00000014072 | 2.72 |
| A_52_P407946 | 5830410O09Rik | 2.72 |
| A_51_P279994 | ENSMUST00000090583 | 2.72 |
| A_52_P7513 | 4932411N23Rik | 2.72 |
| A_52_P740427 | LOC624219 | 2.72 |
| A_52_P344098 | Pde7b | 2.72 |
| A_52_P130423 | LOC238829 | 2.71 |
| A_51_P231820 | C130026I21Rik | 2.71 |
| A_52_P314723 | 2700059L22Rik | 2.71 |
| A_52_P326096 | Meox2 | 2.71 |
| A_52_P302051 | ENSMUST00000098505 | 2.71 |
| A_52_P763537 | AK083787 | 2.71 |
| A_52_P244349 | Dcx | 2.71 |
| A_51_P258473 | 4931429I11Rik | 2.71 |
| A_52_P947796 | AK050779 | 2.71 |
| A_52_P577223 | Atpbd4 | 2.71 |
| A_52_P212756 | Tor1aip2 | 2.71 |
| A_51_P490924 | Hapln4 | 2.71 |
| A_51_P384967 | M29244 | 2.70 |
| A_51_P300022 | AK038314 | 2.70 |
| A_51_P369200 | Tpx2 | 2.70 |
| A_52_P1101503 | A_52_P1101503 | 2.70 |
| A_52_P787372 | AK037050 | 2.70 |
| A_52_P412574 | 4930451I11Rik | 2.70 |
| A_52_P770829 | AK039039 | 2.70 |
| A_52_P495251 | NAP102981-1 | 2.70 |
| A_52_P117922 | LOC545547 | 2.70 |
| A_51_P149531 | Olfr126 | 2.70 |
| A_52_P676774 | AK040149 | 2.70 |
| A_51_P123892 | Lrrtm4 | 2.70 |
| A_52_P594854 | Olfr980 | 2.69 |
| A_52_P755660 | AK086459 | 2.69 |
| A_51_P438236 | 4921520P21Rik | 2.69 |
| A_52_P502771 | E130016E03Rik | 2.69 |
| A_52_P87843 | Aldh1a3 | 2.69 |
| A_52_P511634 | AI317237 | 2.69 |
| A_52_P97889 | B4galnt4 | 2.68 |
| A_51_P374737 | Ovch2 | 2.68 |
| A_51_P467629 | 4930504H06Rik | 2.68 |
| A_52_P413851 | AK053748 | 2.68 |
| A_52_P549500 | NAP035567-1 | 2.68 |
| A_52_P761882 | Lrrn1 | 2.68 |
| A_51_P433333 | Gprc5d | 2.68 |
| A_52_P753819 | 2810409C01Rik | 2.67 |
| A_52_P114703 | Sox11 | 2.67 |
| A_52_P437662 | Cspg5 | 2.67 |
| A_52_P184028 | ENSMUST00000098803 | 2.67 |
| A_52_P115763 | C330024D21Rik | 2.67 |
| A_51_P466558 | ENSMUST00000043793 | 2.67 |
| A_52_P1110812 | TC1453066 | 2.67 |
| A_51_P145322 | Pax1 | 2.67 |
| A_52_P779862 | LOC432436 | 2.66 |
| A_51_P319805 | Mrgprg | 2.66 |
| A_51_P346874 | 1700112E06Rik | 2.66 |
| A_52_P538363 | Pcdhb2 | 2.66 |
| A_51_P366435 | Wnt1 | 2.65 |
| A_51_P174840 | Olfr196 | 2.65 |
| A_52_P1019369 | 4833415N18Rik | 2.65 |
| A_51_P490618 | Olfr1164 | 2.65 |
| A_52_P440260 | Gpm6b | 2.65 |
| A_52_P418042 | Wfikkn2 | 2.65 |
| A_52_P71704 | Kcne1 | 2.65 |
| A_51_P445882 | AK018014 | 2.65 |
| A_51_P241653 | 4831440D22Rik | 2.65 |
| A_51_P386870 | Sprr2f | 2.64 |
| A_52_P313861 | Prdx4 | 2.64 |
| A_52_P646059 | Kif11 | 2.64 |
| A_51_P407746 | 4930512M02Rik | 2.64 |
| A_52_P255034 | 4930547N16Rik | 2.64 |
| A_51_P406613 | BE656555 | 2.64 |
| A_51_P337161 | Kcnj5 | 2.64 |
| A_51_P389278 | Lrrc9 | 2.64 |
| A_52_P108223 | D430004P15Rik | 2.64 |
| A_51_P515262 | Amot | 2.64 |
| A_51_P156849 | Paqr3 | 2.64 |
| A_52_P35202 | 9430041J12Rik | 2.63 |
| A_51_P215559 | BC021614 | 2.63 |
| A_52_P1140007 | AK040747 | 2.62 |
| A_52_P300533 | 2310043M15Rik | 2.62 |
| A_51_P384243 | Dusp13 | 2.62 |
| A_52_P311263 | Lonrf2 | 2.62 |
| A_52_P598862 | 2310081J21Rik | 2.62 |
| A_51_P437327 | Ascl1 | 2.62 |
| A_51_P173957 | Herc1 | 2.62 |
| A_51_P185499 | Prkar1b | 2.62 |
| A_51_P356579 | 1810057P16Rik | 2.62 |
| A_51_P103396 | Krt85 | 2.62 |
| A_51_P435764 | Pdcd11 | 2.62 |
| A_51_P261340 | Faim2 | 2.62 |
| A_51_P116465 | 1700092E16Rik | 2.62 |
| A_52_P649590 | 2310031A07Rik | 2.62 |
| A_51_P137789 | C130092O11Rik | 2.61 |
| A_52_P270175 | Mmp28 | 2.61 |
| A_51_P362554 | Olfr1307 | 2.61 |
| A_51_P177364 | Tas2r105 | 2.61 |
| A_51_P110640 | Klk1b11 | 2.61 |
| A_51_P263749 | A630050E13Rik | 2.61 |
| A_51_P377528 | Oprk1 | 2.61 |
| A_52_P622850 | Hes5 | 2.61 |
| A_52_P827601 | AK040039 | 2.61 |
| A_51_P445677 | Hhat | 2.61 |
| A_51_P303160 | Arg1 | 2.61 |
| A_51_P403393 | 1700065I17Rik | 2.60 |
| A_51_P207751 | Guca1a | 2.60 |
| A_51_P199249 | Olfr272 | 2.60 |
| A_51_P307840 | Cst8 | 2.60 |
| A_52_P1051455 | 4930473M17Rik | 2.60 |
| A_52_P374882 | Lep | 2.60 |
| A_52_P682352 | Tmem44 | 2.60 |
| A_52_P127130 | Msr1 | 2.60 |
| A_51_P508394 | Olfr1032 | 2.59 |
| A_52_P610965 | St18 | 2.59 |
| A_52_P363051 | Myh8 | 2.59 |
| A_51_P504053 | AK076973 | 2.59 |
| A_52_P309797 | NAP037914-1 | 2.59 |
| A_52_P206322 | U76382 | 2.59 |
| A_52_P793792 | 1500002O10Rik | 2.59 |
| A_51_P263853 | Tspan11 | 2.59 |
| A_52_P313992 | 1700007H22Rik | 2.59 |
| A_52_P384690 | Tex11 | 2.59 |
| A_52_P59206 | Cst6 | 2.59 |
| A_52_P852048 | AK084424 | 2.58 |
| A_51_P486320 | 1700042B14Rik | 2.58 |
| A_52_P677276 | Ttll3 | 2.58 |
| A_51_P338206 | Pde1c | 2.58 |
| A_51_P470214 | AI844789 | 2.58 |
| A_52_P795220 | AK087599 | 2.58 |
| A_51_P278070 | BE980334 | 2.58 |
| A_51_P222993 | 0710005I19Rik | 2.58 |
| A_52_P215263 | C130022M03Rik | 2.57 |
| A_51_P271395 | Olfr1206 | 2.57 |
| A_51_P281778 | 2210010C17Rik | 2.57 |
| A_52_P249514 | Ccl12 | 2.57 |
| A_52_P263076 | BC068157 | 2.57 |
| A_52_P14158 | AK053240 | 2.57 |
| A_52_P1003845 | Lcmt1 | 2.56 |
| A_52_P93393 | BC049816 | 2.56 |
| A_52_P746555 | 5033418A18Rik | 2.56 |
| A_52_P616949 | Pvrl4 | 2.56 |
| A_52_P534543 | Iqce | 2.56 |
| A_52_P94937 | 4930592I03Rik | 2.56 |
| A_51_P435545 | AK031438 | 2.56 |
| A_51_P354857 | Bnipl | 2.56 |
| A_51_P459215 | AK038504 | 2.56 |
| A_52_P1020684 | AK079544 | 2.55 |
| A_52_P504159 | LOC236892 | 2.55 |
| A_52_P639765 | Btbd16 | 2.55 |
| A_52_P834112 | 3021401N23Rik | 2.55 |
| A_51_P144024 | Trpa1 | 2.55 |
| A_52_P296233 | LOC277203 | 2.55 |
| A_51_P399905 | ENSMUST00000087544 | 2.55 |
| A_52_P1052029 | AK031632 | 2.55 |
| A_52_P412645 | Rxfp1 | 2.55 |
| A_51_P262453 | ENSMUST00000053727 | 2.54 |
| A_51_P216165 | Olfr560 | 2.54 |
| A_51_P237806 | Olfr1284 | 2.54 |
| A_52_P574618 | Atbf1 | 2.54 |
| A_52_P596372 | AK041466 | 2.54 |
| A_52_P559550 | NAP097318-001 | 2.54 |
| A_51_P170562 | Cpn2 | 2.54 |
| A_52_P579993 | 9330151L19Rik | 2.54 |
| A_52_P225912 | Olfr78 | 2.54 |
| A_52_P174569 | Hdhd1a | 2.54 |
| A_51_P231364 | D5Ertd135e | 2.53 |
| A_51_P233145 | Ssty2 | 2.53 |
| A_51_P324473 | 9130014G24Rik | 2.53 |
| A_52_P477573 | Crtac1 | 2.53 |
| A_52_P1172661 | A430050A11Rik | 2.53 |
| A_52_P460734 | Crhbp | 2.53 |
| A_51_P423518 | Amph | 2.53 |
| A_51_P270661 | Igh-VJ558 | 2.52 |
| A_51_P195534 | Ankrd22 | 2.52 |
| A_51_P342031 | Mkrn3 | 2.52 |
| A_52_P613608 | Myo3a | 2.52 |
| A_51_P388661 | Msx2 | 2.52 |
| A_52_P416086 | Wfdc3 | 2.52 |
| A_51_P422685 | Zmat4 | 2.52 |
| A_51_P229759 | Pxdn | 2.52 |
| A_51_P171180 | 2310033K02Rik | 2.52 |
| A_51_P403260 | Diap3 | 2.51 |
| A_52_P641991 | Stmn2 | 2.51 |
| A_52_P371388 | NAP026976-1 | 2.51 |
| A_52_P231729 | H2-Q1 | 2.51 |
| A_52_P891888 | AK040215 | 2.51 |
| A_51_P479029 | Trpv6 | 2.51 |
| A_51_P311459 | 4930485B16Rik | 2.51 |
| A_52_P296006 | E030003N13Rik | 2.51 |
| A_51_P248008 | Olfr1109 | 2.51 |
| A_52_P452268 | D330037H05Rik | 2.51 |
| A_51_P406253 | Gcg | 2.51 |
| A_52_P281186 | Mep1b | 2.50 |
| A_52_P202045 | Lrp1 | 2.50 |
| A_51_P353934 | Nkx2-4 | 2.50 |
| A_51_P132343 | AK053945 | 2.50 |
| A_51_P354616 | Tcrg | 2.50 |
| A_51_P137709 | Calml3 | 2.50 |
| A_51_P436813 | 5430405H02Rik | 2.50 |
| A_52_P286288 | Olfr474 | 2.50 |
| A_52_P820019 | AK076665 | 2.49 |
| A_51_P504613 | AK078580 | 2.49 |
| A_51_P455166 | Prlpa | 2.49 |
| A_51_P115934 | C630040K21Rik | 2.49 |
| A_51_P476454 | Olfr456 | 2.49 |
| A_51_P418374 | Gpr87 | 2.49 |
| A_52_P277220 | 1110033F14Rik | 2.49 |
| A_52_P570861 | Klhl5 | 2.49 |
| A_51_P386625 | Epx | 2.49 |
| A_52_P8241 | 4930518J20Rik | 2.49 |
| A_51_P243063 | 2810441K11Rik | 2.48 |
| A_52_P223269 | Zfp37 | 2.48 |
| A_51_P360518 | Olfr1009 | 2.48 |
| A_51_P150242 | Olfr1362 | 2.48 |
| A_52_P151864 | AF366264 | 2.48 |
| A_52_P244463 | D16Ertd472e | 2.47 |
| A_51_P164256 | ENSMUST00000033334 | 2.47 |
| A_51_P196997 | Soat2 | 2.47 |
| A_52_P248513 | Klra7 | 2.46 |
| A_52_P569846 | Stfna3l1 | 2.46 |
| A_51_P229664 | Tnfrsf7 | 2.46 |
| A_52_P62121 | Gpr37 | 2.46 |
| A_51_P399143 | Rbp2 | 2.46 |
| A_51_P494229 | Galnt4 | 2.46 |
| A_51_P519632 | Olfr661 | 2.46 |
| A_51_P153683 | BC004853 | 2.45 |
| A_52_P193161 | Fgf12 | 2.45 |
| A_52_P22617 | LOC229879 | 2.45 |
| A_52_P540159 | Tbrg3 | 2.45 |
| A_51_P138002 | Zfp26 | 2.44 |
| A_51_P178903 | 1700024D23Rik | 2.44 |
| A_51_P309095 | AK050362 | 2.44 |
| A_52_P67250 | Tmem102 | 2.44 |
| A_51_P421300 | ENSMUST00000026541 | 2.44 |
| A_52_P204843 | 4930435E12Rik | 2.44 |
| A_51_P422360 | Pthr2 | 2.44 |
| A_52_P448510 | Katnb1 | 2.43 |
| A_52_P416618 | Lmo7 | 2.43 |
| A_52_P346860 | Rttn | 2.43 |
| A_52_P285207 | D630013G24Rik | 2.43 |
| A_52_P363371 | Lrrc37a | 2.43 |
| A_52_P224641 | Rdhe2 | 2.43 |
| A_52_P31632 | B930018B01 | 2.43 |
| A_51_P224938 | Grm6 | 2.43 |
| A_52_P851431 | AK048105 | 2.43 |
| A_51_P351923 | A030009H04Rik | 2.42 |
| A_52_P82704 | Bcl11a | 2.42 |
| A_52_P209944 | C130071C03Rik | 2.42 |
| A_51_P315964 | Nr5a1 | 2.42 |
| A_52_P908046 | 4632404H22Rik | 2.42 |
| A_52_P268269 | BC010787 | 2.42 |
| A_51_P261388 | Mgat5b | 2.42 |
| A_52_P639108 | D030022P07Rik | 2.41 |
| A_51_P239750 | Inhba | 2.41 |
| A_52_P1179549 | 6820402A03Rik | 2.41 |
| A_52_P518613 | Adam23 | 2.41 |
| A_51_P471900 | Stk3 | 2.41 |
| A_52_P104444 | NAP062835-1 | 2.40 |
| A_52_P599728 | Mtap1a | 2.40 |
| A_51_P237327 | Olfr1509 | 2.40 |
| A_52_P682293 | C630013B14Rik | 2.40 |
| A_52_P304991 | Olfr153 | 2.40 |
| A_52_P569707 | AK079494 | 2.40 |
| A_52_P378181 | 4930527E24Rik | 2.40 |
| A_51_P370363 | 1700030L22Rik | 2.39 |
| A_51_P126258 | Pcdhga1 | 2.39 |
| A_52_P947482 | AK045058 | 2.39 |
| A_51_P212012 | 8430419K02Rik | 2.39 |
| A_51_P323358 | AK033234 | 2.39 |
| A_52_P8795 | Ccdc15 | 2.39 |
| A_51_P503736 | 4921530G04Rik | 2.39 |
| A_52_P226307 | AK085332 | 2.39 |
| A_52_P923921 | B230118I11Rik | 2.39 |
| A_51_P364592 | Zic2 | 2.39 |
| A_51_P237245 | AK054300 | 2.39 |
| A_51_P196937 | U64446 | 2.39 |
| A_51_P137236 | Olfm1 | 2.38 |
| A_52_P308593 | 4930432J09Rik | 2.38 |
| A_52_P723574 | AK083799 | 2.38 |
| A_52_P1012157 | AK051389 | 2.38 |
| A_52_P474961 | Fsip1 | 2.38 |
| A_52_P122649 | Dmrta1 | 2.38 |
| A_52_P269143 | D630004D15Rik | 2.38 |
| A_51_P441231 | NAP057196-1 | 2.38 |
| A_52_P647226 | Ing4 | 2.38 |
| A_51_P103133 | A_51_P103133 | 2.38 |
| A_52_P794661 | AK028499 | 2.38 |
| A_52_P1132525 | AK082545 | 2.37 |
| A_52_P607393 | AK090226 | 2.37 |
| A_51_P382647 | Olfr399 | 2.37 |
| A_51_P196148 | Atf7ip2 | 2.37 |
| A_52_P341733 | Prmt6 | 2.37 |
| A_51_P213260 | Gata5 | 2.37 |
| A_52_P558502 | Il16 | 2.37 |
| A_51_P133428 | Cacng6 | 2.37 |
| A_52_P240036 | Plch1 | 2.37 |
| A_52_P209818 | AK038377 | 2.37 |
| A_52_P43578 | D130017N08Rik | 2.37 |
| A_51_P376590 | BB161836 | 2.36 |
| A_52_P1163884 | AK039077 | 2.36 |
| A_51_P285856 | 4930503E14Rik | 2.36 |
| A_52_P174915 | Gja1 | 2.36 |
| A_52_P291924 | Ncam1 | 2.36 |
| A_51_P427964 | Lmod3 | 2.36 |
| A_52_P417148 | Apex2 | 2.36 |
| A_52_P143332 | 1700013D24Rik | 2.36 |
| A_52_P464096 | Eral1 | 2.36 |
| A_51_P154679 | Tbc1d24 | 2.36 |
| A_51_P438847 | Ctnna2 | 2.36 |
| A_52_P1034723 | Tceal7 | 2.35 |
| A_52_P129238 | Adck1 | 2.35 |
| A_52_P657537 | 4930552N02Rik | 2.35 |
| A_51_P241426 | Gfra4 | 2.35 |
| A_51_P346565 | Sdk2 | 2.35 |
| A_52_P1187851 | A230092J17Rik | 2.35 |
| A_51_P345449 | Olfr39 | 2.35 |
| A_51_P145220 | Nef3 | 2.34 |
| A_51_P151133 | 9930032O22Rik | 2.34 |
| A_52_P644465 | Ywhaz | 2.34 |
| A_51_P362566 | Trip11 | 2.34 |
| A_52_P875073 | A930019D19Rik | 2.34 |
| A_51_P264644 | 9530018H14Rik | 2.34 |
| A_51_P280893 | Pla2g1b | 2.34 |
| A_51_P274674 | Olfr1282 | 2.34 |
| A_52_P287692 | Stk32c | 2.34 |
| A_52_P530066 | Unc13c | 2.34 |
| A_51_P317542 | 6330417K15Rik | 2.34 |
| A_52_P593379 | Pax9 | 2.34 |
| A_52_P69109 | Slc10a1 | 2.34 |
| A_51_P206907 | Olfr1056 | 2.33 |
| A_52_P649210 | A630098A13Rik | 2.33 |
| A_51_P297498 | Sln | 2.33 |
| A_51_P103912 | Mc5r | 2.33 |
| A_52_P442432 | NAP058243-1 | 2.33 |
| A_52_P421713 | 9930012K11Rik | 2.33 |
| A_52_P298732 | AK085088 | 2.32 |
| A_52_P1132687 | AK089699 | 2.32 |
| A_51_P423157 | R3hdm1 | 2.32 |
| A_51_P251487 | 4921504I05Rik | 2.32 |
| A_51_P102538 | Otop1 | 2.32 |
| A_51_P491742 | Uhrf1 | 2.32 |
| A_51_P112833 | Pkd1l2 | 2.32 |
| A_52_P94941 | Gsdm1 | 2.32 |
| A_51_P202942 | Cox4i2 | 2.32 |
| A_52_P69246 | D32072 | 2.32 |
| A_52_P49080 | Otx2 | 2.31 |
| A_52_P370031 | AK043317 | 2.31 |
| A_52_P32112 | LOC667885 | 2.31 |
| A_52_P257638 | Myh1 | 2.31 |
| A_51_P287715 | 3100003L13Rik | 2.31 |
| A_51_P135920 | Nck2 | 2.31 |
| A_51_P487628 | Pramef12 | 2.31 |
| A_51_P487628 | Pramef12 | 2.31 |
| A_52_P900467 | AK079188 | 2.31 |
| A_51_P397876 | En2 | 2.31 |
| A_52_P907806 | AK042396 | 2.30 |
| A_51_P458638 | C630041L24Rik | 2.30 |
| A_51_P159492 | Ocrl | 2.30 |
| A_52_P38499 | A830025P08Rik | 2.30 |
| A_52_P628915 | Syt4 | 2.30 |
| A_51_P150145 | AK077046 | 2.30 |
| A_51_P486207 | AK032580 | 2.30 |
| A_52_P1044262 | AK051949 | 2.30 |
| A_52_P395342 | 4930412M03Rik | 2.29 |
| A_52_P157595 | Rhox3 | 2.29 |
| A_52_P529841 | TC1453715 | 2.29 |
| A_52_P440481 | A630033E08Rik | 2.29 |
| A_51_P244034 | Rexo4 | 2.29 |
| A_51_P160463 | AK038819 | 2.29 |
| A_51_P227564 | Ccdc99 | 2.29 |
| A_52_P331824 | Nfasc | 2.28 |
| A_51_P283359 | GA_x5J8B7W2BV0-3116-4045 | 2.28 |
| A_52_P747490 | AK052118 | 2.28 |
| A_52_P207303 | Bmp3 | 2.28 |
| A_51_P102319 | Dock11 | 2.28 |
| A_51_P439531 | 4930478A21Rik | 2.28 |
| A_51_P369657 | AK083533 | 2.28 |
| A_52_P545650 | Krt36 | 2.28 |
| A_51_P277994 | Oas2 | 2.28 |
| A_52_P672692 | Gpr45 | 2.28 |
| A_52_P612928 | Gabbr2 | 2.28 |
| A_52_P747196 | AK048779 | 2.28 |
| A_51_P221294 | D1Ertd471e | 2.28 |
| A_52_P123924 | B230317F23Rik | 2.28 |
| A_51_P516870 | Itm2a | 2.28 |
| A_52_P558931 | D930038D03Rik | 2.28 |
| A_51_P321011 | AK083865 | 2.28 |
| A_51_P383489 | Fignl1 | 2.27 |
| A_51_P393288 | Olfr447 | 2.27 |
| A_51_P485756 | Nts | 2.27 |
| A_51_P324450 | Pbp2 | 2.27 |
| A_52_P668812 | Sgol2 | 2.27 |
| A_52_P451024 | 4930511H11Rik | 2.27 |
| A_52_P932240 | AK082846 | 2.27 |
| A_51_P295896 | 4930452B06Rik | 2.26 |
| A_52_P275786 | Clm3 | 2.26 |
| A_52_P56818 | Foxp2 | 2.26 |
| A_51_P494751 | Mael | 2.26 |
| A_52_P41759 | Kif3c | 2.26 |
| A_51_P114941 | Cyp3a13 | 2.26 |
| A_52_P268467 | 4930449C09Rik | 2.26 |
| A_52_P65050 | Ect2 | 2.26 |
| A_52_P180283 | Phf20l1 | 2.26 |
| A_51_P235345 | 9830001H06Rik | 2.26 |
| A_52_P409457 | Ppcdc | 2.26 |
| A_52_P476775 | Mdfi | 2.26 |
| A_52_P470462 | Zfp422-rs1 | 2.25 |
| A_52_P222501 | NAP122972-1 | 2.25 |
| A_51_P352452 | LOC667665 | 2.25 |
| A_52_P441924 | Fpr-rs7 | 2.25 |
| A_52_P1016836 | LOC639426 | 2.25 |
| A_51_P145360 | Sdk1 | 2.25 |
| A_51_P307076 | 1190001M18Rik | 2.25 |
| A_51_P335112 | Upf2 | 2.25 |
| A_52_P518257 | A130014H13Rik | 2.25 |
| A_51_P329084 | Shprh | 2.25 |
| A_51_P494491 | Phox2b | 2.24 |
| A_52_P738960 | AK043345 | 2.24 |
| A_52_P144946 | 4930506M07Rik | 2.24 |
| A_51_P447894 | Tgm4 | 2.24 |
| A_51_P196113 | Mast1 | 2.24 |
| A_51_P136411 | Dgki | 2.24 |
| A_51_P407213 | Dmpk | 2.23 |
| A_52_P329451 | Mbp | 2.23 |
| A_52_P432647 | Icosl | 2.23 |
| A_52_P287811 | AK051168 | 2.23 |
| A_51_P328202 | Daam2 | 2.23 |
| A_51_P128075 | 1700008P20Rik | 2.23 |
| A_52_P531140 | Hemt1 | 2.23 |
| A_52_P55876 | Runx2 | 2.23 |
| A_52_P244639 | 6330500D04Rik | 2.23 |
| A_51_P472049 | ENSMUST00000080637 | 2.22 |
| A_52_P553069 | Prkg1 | 2.22 |
| A_51_P461368 | Sytl3 | 2.22 |
| A_52_P383399 | NAP071456-1 | 2.22 |
| A_51_P123385 | Olfr658 | 2.22 |
| A_51_P227679 | C030002O17Rik | 2.22 |
| A_51_P274409 | 4930518F03Rik | 2.22 |
| A_51_P403814 | C330016K18Rik | 2.22 |
| A_52_P680761 | Tdrd6 | 2.22 |
| A_52_P404302 | Olfml2a | 2.22 |
| A_51_P270005 | 4921528H16Rik | 2.22 |
| A_51_P120636 | E2f1 | 2.22 |
| A_51_P222651 | AK085052 | 2.22 |
| A_51_P310862 | Zfp455 | 2.22 |
| A_51_P313503 | Olfr577 | 2.21 |
| A_51_P337210 | Lrrc4 | 2.21 |
| A_52_P738493 | 4930528J11Rik | 2.21 |
| A_51_P420128 | 1700066J24Rik | 2.21 |
| A_51_P226499 | AK046172 | 2.21 |
| A_52_P956284 | AK085307 | 2.21 |
| A_52_P75127 | Tcp11 | 2.21 |
| A_52_P52182 | NAP023895-001 | 2.21 |
| A_52_P947707 | AK051216 | 2.21 |
| A_51_P443902 | Klk1b16 | 2.20 |
| A_52_P249283 | LOC385234 | 2.20 |
| A_51_P145041 | Lrp8 | 2.20 |
| A_51_P447540 | 4933401B01Rik | 2.20 |
| A_51_P389185 | Olfr5 | 2.20 |
| A_51_P383530 | A530065I17Rik | 2.20 |
| A_51_P130808 | Tpbpb | 2.20 |
| A_51_P341543 | Defb7 | 2.20 |
| A_52_P463295 | Olfr1141 | 2.20 |
| A_51_P500318 | Sox5 | 2.20 |
| A_51_P501998 | Asb18 | 2.20 |
| A_51_P290387 | Sval1 | 2.20 |
| A_51_P432403 | Fgfr2 | 2.20 |
| A_52_P239976 | Cacna1i | 2.20 |
| A_51_P151848 | Rfxdc1 | 2.20 |
| A_52_P198802 | 4930539E08Rik | 2.20 |
| A_52_P177142 | Otud1 | 2.20 |
| A_52_P92398 | Gm1805 | 2.20 |
| A_52_P35470 | AK042543 | 2.20 |
| A_52_P400149 | 6430562O15Rik | 2.20 |
| A_52_P651987 | Ypel1 | 2.20 |
| A_51_P159612 | Hebp2 | 2.20 |
| A_51_P163979 | Olfr49 | 2.19 |
| A_52_P360330 | BC045135 | 2.19 |
| A_52_P462657 | A430060F13Rik | 2.19 |
| A_52_P477668 | ENSMUST00000020322 | 2.19 |
| A_51_P179258 | BC056349 | 2.19 |
| A_52_P271806 | 9530026P05Rik | 2.19 |
| A_52_P518087 | A230107N01Rik | 2.19 |
| A_52_P732441 | LOC632687 | 2.19 |
| A_51_P189198 | Pard3 | 2.19 |
| A_52_P403538 | LOC638058 | 2.19 |
| A_51_P120027 | Adh6-ps1 | 2.19 |
| A_52_P353155 | Nalp9b | 2.18 |
| A_52_P293120 | Prrg3 | 2.18 |
| A_52_P351378 | Gm94 | 2.18 |
| A_51_P287635 | P2ry2 | 2.18 |
| A_52_P529650 | Drd2 | 2.18 |
| A_51_P233364 | NAP108602-1 | 2.18 |
| A_51_P421538 | C80638 | 2.18 |
| A_52_P52272 | 2210010B09Rik | 2.18 |
| A_52_P515907 | 4930503L19Rik | 2.18 |
| A_51_P446179 | Tmprss4 | 2.18 |
| A_51_P142196 | H19 | 2.18 |
| A_52_P625912 | Klrb1f | 2.18 |
| A_51_P390606 | NAP057053-1 | 2.18 |
| A_51_P342629 | 2200002J24Rik | 2.17 |
| A_52_P981179 | A_52_P981179 | 2.17 |
| A_51_P517645 | 2310014F07Rik | 2.17 |
| A_52_P488908 | Hes7 | 2.17 |
| A_52_P22286 | AK041357 | 2.17 |
| A_52_P148553 | Fignl1 | 2.17 |
| A_51_P125882 | Glp2r | 2.17 |
| A_52_P321244 | 4930544M13Rik | 2.17 |
| A_51_P205209 | Cd244 | 2.16 |
| A_52_P30451 | Ppp1r3c | 2.16 |
| A_51_P469252 | Zfp647 | 2.16 |
| A_52_P577084 | AK019597 | 2.16 |
| A_51_P345344 | 2310043J07Rik | 2.16 |
| A_52_P119814 | Slc13a5 | 2.16 |
| A_52_P601727 | AK052688 | 2.16 |
| A_52_P1197518 | TC1536826 | 2.16 |
| A_52_P491849 | Trp53 | 2.16 |
| A_51_P238009 | Loxhd1 | 2.16 |
| A_51_P115895 | Olfr800 | 2.15 |
| A_52_P764315 | A_52_P764315 | 2.15 |
| A_52_P907712 | AK051542 | 2.15 |
| A_51_P276486 | 1810049H19Rik | 2.15 |
| A_51_P104172 | Zfp128 | 2.15 |
| A_52_P273865 | Pftk1 | 2.15 |
| A_51_P279898 | 4930404N11Rik | 2.15 |
| A_51_P220806 | Gdf9 | 2.14 |
| A_52_P382149 | Cyp26a1 | 2.14 |
| A_51_P371764 | AK048252 | 2.14 |
| A_52_P578744 | 5430416O09Rik | 2.14 |
| A_52_P1123661 | AK041851 | 2.14 |
| A_52_P31093 | NAP093165-001 | 2.14 |
| A_52_P259521 | Rag2 | 2.13 |
| A_52_P596854 | LOC626942 | 2.13 |
| A_51_P115005 | Edn1 | 2.13 |
| A_52_P571801 | Cml1 | 2.13 |
| A_51_P100238 | Olfr323 | 2.13 |
| A_51_P512669 | 4930478L05Rik | 2.13 |
| A_51_P110914 | 1700010D01Rik | 2.13 |
| A_51_P345340 | 2310043J07Rik | 2.13 |
| A_52_P91274 | 1700018G05Rik | 2.13 |
| A_52_P1116362 | AK084420 | 2.13 |
| A_51_P356172 | AK052576 | 2.12 |
| A_52_P686701 | Nfam1 | 2.12 |
| A_52_P600555 | BC002112 | 2.12 |
| A_51_P285725 | T | 2.12 |
| A_51_P492893 | Foxp4 | 2.12 |
| A_51_P418457 | Defb13 | 2.12 |
| A_52_P321751 | Narg2 | 2.12 |
| A_51_P137184 | 1700029J11Rik | 2.12 |
| A_52_P644320 | Sema3e | 2.12 |
| A_51_P286878 | Ttll11 | 2.11 |
| A_52_P576230 | Tacc2 | 2.11 |
| A_52_P18208 | 6720487G11Rik | 2.11 |
| A_52_P1140316 | AK048885 | 2.11 |
| A_51_P273264 | 1500002C15Rik | 2.11 |
| A_52_P199084 | Cd55 | 2.11 |
| A_52_P648012 | TC1438297 | 2.11 |
| A_51_P470905 | Tmem52 | 2.11 |
| A_51_P428410 | ENSMUST00000053562 | 2.11 |
| A_52_P255959 | Gm288 | 2.10 |
| A_52_P553471 | Lip1 | 2.10 |
| A_52_P265937 | Tagln3 | 2.10 |
| A_52_P290944 | Mamdc4 | 2.10 |
| A_52_P254782 | Cntn5 | 2.10 |
| A_52_P1013543 | AI390992 | 2.10 |
| A_52_P630673 | Gpr62 | 2.10 |
| A_52_P662785 | Polr3g | 2.10 |
| A_51_P126684 | Lrrc14 | 2.10 |
| A_51_P435410 | Grid2ip | 2.10 |
| A_52_P650387 | Ccnjl | 2.10 |
| A_52_P283041 | Smarca1 | 2.10 |
| A_51_P355094 | NAP057266-1 | 2.10 |
| A_52_P40777 | Arhgap12 | 2.10 |
| A_52_P211418 | 6030408C04Rik | 2.09 |
| A_52_P392663 | AK078641 | 2.09 |
| A_52_P1188322 | AK051274 | 2.09 |
| A_51_P456114 | 9930013L23Rik | 2.09 |
| A_52_P509679 | 1810015F01Rik | 2.09 |
| A_52_P525348 | ENSMUST00000094405 | 2.09 |
| A_51_P477614 | Pitx3 | 2.09 |
| A_52_P779355 | AK053107 | 2.09 |
| A_52_P48412 | Uevld | 2.08 |
| A_52_P363013 | E230002P03Rik | 2.08 |
| A_52_P416899 | Asb15 | 2.08 |
| A_52_P544523 | Myl4 | 2.08 |
| A_51_P159986 | Olfr1423 | 2.08 |
| A_51_P230098 | Pbk | 2.08 |
| A_51_P232667 | A230063L24Rik | 2.08 |
| A_52_P470046 | Cyfip2 | 2.08 |
| A_51_P311372 | AK039234 | 2.08 |
| A_52_P63850 | BC052055 | 2.08 |
| A_51_P447471 | Gtdc1 | 2.08 |
| A_52_P1003865 | AK047056 | 2.08 |
| A_52_P1035662 | AK032159 | 2.08 |
| A_52_P474024 | Auts2 | 2.07 |
| A_52_P707475 | AK053952 | 2.07 |
| A_52_P502141 | Hectd2 | 2.07 |
| A_52_P498193 | Aldh1l2 | 2.07 |
| A_51_P386069 | Rab9b | 2.07 |
| A_51_P368237 | Dnahc1 | 2.07 |
| A_52_P280253 | LOC631256 | 2.07 |
| A_52_P112033 | 1700065D16Rik | 2.07 |
| A_51_P298688 | Fastkd1 | 2.07 |
| A_52_P787717 | AK082197 | 2.07 |
| A_51_P220976 | Ifng | 2.07 |
| A_51_P399945 | Hpvc2 | 2.06 |
| A_51_P498475 | Calr4 | 2.06 |
| A_52_P1124598 | AK081090 | 2.06 |
| A_52_P511005 | TC1487280 | 2.06 |
| A_51_P438278 | C130038G02Rik | 2.06 |
| A_51_P100218 | V1rf1 | 2.06 |
| A_51_P459741 | Gprasp1 | 2.06 |
| A_52_P376618 | Spata17 | 2.06 |
| A_52_P572456 | Isg20l2 | 2.06 |
| A_51_P129999 | 4933412E14Rik | 2.06 |
| A_52_P228621 | Tnfrsf1b | 2.06 |
| A_52_P210424 | Cacna1a | 2.06 |
| A_51_P429715 | Lbx1 | 2.05 |
| A_52_P161780 | Galnt13 | 2.05 |
| A_52_P480266 | 2610110G12Rik | 2.05 |
| A_52_P237371 | Col5a1 | 2.05 |
| A_52_P40504 | 2900052L18Rik | 2.05 |
| A_52_P1029636 | TC1468432 | 2.05 |
| A_52_P564724 | CB587035 | 2.04 |
| A_52_P387882 | AI595406 | 2.04 |
| A_52_P915781 | AK038901 | 2.04 |
| A_51_P373498 | Olfr572 | 2.04 |
| A_51_P394745 | Fryl | 2.04 |
| A_51_P120738 | P2ry14 | 2.04 |
| A_52_P24793 | Eif2c3 | 2.04 |
| A_52_P383089 | Cd4 | 2.04 |
| A_52_P29615 | Rab27a | 2.04 |
| A_52_P102260 | C330001K17Rik | 2.04 |
| A_51_P216108 | Rps6kl1 | 2.04 |
| A_51_P156309 | AK047917 | 2.04 |
| A_51_P248982 | AK085452 | 2.03 |
| A_51_P390857 | AK054376 | 2.03 |
| A_52_P408245 | Fmo1 | 2.03 |
| A_51_P134045 | Pcsk2 | 2.03 |
| A_52_P175046 | Ranbp17 | 2.03 |
| A_51_P105380 | 2010005H15Rik | 2.03 |
| A_51_P249274 | 1300017J02Rik | 2.03 |
| A_51_P183912 | Il27ra | 2.03 |
| A_51_P470304 | Nkx1-2 | 2.03 |
| A_52_P15377 | Wnt9b | 2.03 |
| A_51_P439311 | 1810041L15Rik | 2.03 |
| A_52_P474699 | 0610025J13Rik | 2.03 |
| A_52_P1085235 | A_52_P1085235 | 2.03 |
| A_51_P308227 | Mpp7 | 2.03 |
| A_52_P421220 | Dzip1 | 2.03 |
| A_51_P244020 | Rtbdn | 2.02 |
| A_52_P239556 | Acta2 | 2.02 |
| A_51_P418859 | BC050092 | 2.02 |
| A_52_P154725 | 2810021J22Rik | 2.02 |
| A_51_P490286 | BC016201 | 2.02 |
| A_52_P330524 | B930011P16Rik | 2.02 |
| A_51_P409068 | Smug1 | 2.02 |
| A_51_P148509 | Ttn | 2.02 |
| A_52_P284535 | Ftmt | 2.02 |
| A_52_P630806 | Tac1 | 2.02 |
| A_52_P253025 | Neo1 | 2.02 |
| A_51_P216456 | Tac1 | 2.02 |
| A_52_P1148119 | A730094K22Rik | 2.01 |
| A_51_P163953 | Nsg2 | 2.01 |
| A_51_P264336 | Abcc6 | 2.01 |
| A_51_P285779 | Asphd2 | 2.01 |
| A_52_P412362 | Olfr788 | 2.01 |
| A_51_P342622 | Ccdc28a | 2.01 |
| A_52_P365480 | Tnfsf13b | 2.01 |
| A_52_P169087 | AK008281 | 2.01 |
| A_51_P261405 | C430014M02Rik | 2.01 |
| A_52_P11529 | D130009I18Rik | 2.01 |
| A_52_P169212 | 1700072H12Rik | 2.01 |
| A_51_P500082 | LOC667373 | 2.01 |
| A_52_P1091593 | AK035003 | 2.00 |
| A_52_P715416 | AK053597 | 2.00 |
| A_52_P183239 | Scn8a | 2.00 |
| A_52_P384394 | Bmf | 2.00 |
| A_51_P124535 | Mest | 2.00 |
