# Supplementary material for: Aqp5 Is a New Transcriptional Target of Dot1a and a Regulator of Aqp2
Source: PLoS One. 2013 Jan 10;8(1):e53342. doi: 10.1371/journal.pone.0053342 (PMC3542343; doi:10.1371/journal.pone.0053342)
Supplement: Table S2 — Downregulated genes in Dot1lAC vs. Dot1lf/f mice. As in Table S1, total kidney RNA of Dot1lf/f and Dot1lAC mice (n = 4 mice/genotype) was subjected to microarray analyses. There 627 genes represented by 680 unique probes. These genes were downregulated with at least ≥2-fold lower mRNA levels in Dot1lAC vs. Dot1lf/f mice. (DOC) [file pone.0053342.s006.doc]

**Table S2. Downregulated genes in *Dot1l^AC^* vs. *Dot1l^f/f^*mice.** As in Table S1, total kidney RNA of *Dot1l^f/f^* and *Dot1l^AC^* mice (n=4 mice/genotype) was subjected to microarray analyses. There 627 genes represented by 680 unique probes. These genes were downregulated with at least ≥ 2-fold lower mRNA levels in *Dot1l^AC^* vs. *Dot1l^f/f^* mice.

| **Probe ID** | **Gene ID** | **Fold change**  **(*Dot1l^AC^* vs. *Dot1l^f/f^*)** |
| --- | --- | --- |
| A_52_P262219 | Fos | -239.39 |
| A_51_P173043 | Egr2 | -76.77 |
| A_51_P367866 | Egr1 | -73.44 |
| A_51_P239654 | Nr4a1 | -26.92 |
| A_51_P256827 | S100a8 | -17.68 |
| A_52_P254817 | Retnla | -15.58 |
| A_51_P363187 | Cxcl1 | -12.69 |
| A_51_P503494 | Arc | -11.45 |
| A_51_P183571 | Serpine1 | -9.38 |
| A_51_P402943 | S100a9 | -8.85 |
| A_52_P452689 | Atf3 | -8.58 |
| A_52_P494622 | Nr4a2 | -8.21 |
| A_51_P436652 | Ccl7 | -7.64 |
| A_51_P286737 | Ccl2 | -7.58 |
| A_51_P481325 | Ier2 | -7.00 |
| A_52_P925197 | Btg2 | -6.98 |
| A_51_P159201 | Junb | -6.90 |
| A_52_P464640 | Exph5 | -6.70 |
| A_51_P112966 | Ch25h | -6.37 |
| A_51_P252859 | Cyr61 | -5.94 |
| A_51_P212782 | Il1b | -5.84 |
| A_51_P375201 | Plk3 | -5.73 |
| A_52_P286166 | Penk1 | -5.57 |
| A_51_P325914 | Jun | -5.47 |
| A_52_P484956 | Nnat | -5.46 |
| A_51_P445574 | Nags | -5.11 |
| A_52_P569499 | Stat3 | -5.09 |
| A_51_P516012 | Ntrk2 | -4.96 |
| A_52_P533841 | Cyp4f16 | -4.95 |
| A_52_P88648 | Cyp24a1 | -4.72 |
| A_52_P30341 | D430040G12Rik | -4.49 |
| A_52_P80944 | Zfp36 | -4.43 |
| A_52_P401504 | Thbs4 | -4.31 |
| A_51_P507801 | F13a1 | -4.26 |
| A_52_P684087 | Atoh7 | -4.23 |
| A_52_P604629 | Axud1 | -4.10 |
| A_52_P448994 | AK086129 | -4.09 |
| A_52_P48569 | Slc38a4 | -4.08 |
| A_52_P571987 | Hspa4 | -4.07 |
| A_52_P425839 | Retnlg | -3.96 |
| A_52_P131423 | Mbd1 | -3.87 |
| A_51_P117952 | Hspa1a | -3.81 |
| A_51_P374571 | Igfbp6 | -3.81 |
| A_51_P358042 | AK035139 | -3.78 |
| A_51_P194149 | Ttr | -3.78 |
| A_51_P403705 | BC030462 | -3.77 |
| A_52_P52849 | Cpxm2 | -3.75 |
| A_52_P474528 | NAP066348-1 | -3.75 |
| A_51_P331752 | Ccl11 | -3.71 |
| A_51_P416858 | Myl1 | -3.70 |
| A_51_P308817 | V1rc12 | -3.70 |
| A_51_P428056 | LOC634065 | -3.64 |
| A_52_P332187 | Ankrd17 | -3.64 |
| A_51_P249286 | Rgs16 | -3.62 |
| A_52_P686392 | Igh-VJ558 | -3.60 |
| A_52_P715191 | AK037579 | -3.55 |
| A_52_P114829 | Crygc | -3.54 |
| A_52_P164570 | Hsd17b12 | -3.53 |
| A_51_P181565 | Hbegf | -3.53 |
| A_51_P493649 | Sult1e1 | -3.52 |
| A_52_P601264 | Itgav | -3.52 |
| A_51_P412579 | 2310079P10Rik | -3.51 |
| A_51_P403704 | 2610100L16Rik | -3.47 |
| A_52_P411814 | TC1428970 | -3.41 |
| A_51_P407323 | F5 | -3.41 |
| A_52_P620100 | Cct3 | -3.41 |
| A_52_P519191 | Dio3as | -3.35 |
| A_52_P1179988 | AK050242 | -3.33 |
| A_52_P714654 | 8030488J09Rik | -3.27 |
| A_52_P652859 | Lama2 | -3.27 |
| A_52_P432715 | AK078446 | -3.25 |
| A_52_P278549 | Myc | -3.25 |
| A_51_P436596 | Rph3a | -3.23 |
| A_51_P101975 | Fgf8 | -3.23 |
| A_52_P544435 | ENSMUST00000101328 | -3.21 |
| A_51_P153486 | Dnajb1 | -3.21 |
| A_51_P437089 | 4930449I24Rik | -3.21 |
| A_52_P608322 | Maff | -3.20 |
| A_52_P1147628 | AK040096 | -3.20 |
| A_51_P486239 | Clec3b | -3.19 |
| A_51_P254855 | Ptgs2 | -3.17 |
| A_52_P313206 | D630023F18Rik | -3.15 |
| A_52_P1156816 | LOC552906 | -3.15 |
| A_52_P172838 | Fath2 | -3.14 |
| A_52_P128134 | Foxd1 | -3.14 |
| A_52_P290926 | Dhrs7c | -3.12 |
| A_51_P305537 | St3gal2 | -3.11 |
| A_52_P496726 | Rasd1 | -3.10 |
| A_51_P507242 | Fosl2 | -3.09 |
| A_52_P61735 | Flnc | -3.08 |
| A_51_P124748 | Tgfb3 | -3.08 |
| A_51_P467699 | 4930556J24Rik | -3.07 |
| A_51_P482990 | Arid5a | -3.07 |
| A_51_P318192 | Gem | -3.06 |
| A_51_P117195 | C030010B13Rik | -3.04 |
| A_52_P517683 | Tagln | -3.01 |
| A_52_P532769 | ENSMUST00000084869 | -2.99 |
| A_52_P461151 | NAP070462-1 | -2.99 |
| A_51_P421780 | Myo18b | -2.99 |
| A_51_P413910 | Hmx3 | -2.97 |
| A_52_P14496 | AK132653 | -2.97 |
| A_51_P250049 | AK087715 | -2.95 |
| A_51_P387108 | 4930535E02Rik | -2.95 |
| A_52_P430211 | Arhgap17 | -2.94 |
| A_52_P384916 | AK136384 | -2.94 |
| A_51_P334155 | 4930579F01Rik | -2.93 |
| A_51_P104392 | Rpp25 | -2.93 |
| A_51_P182116 | Dscr1 | -2.92 |
| A_51_P246854 | Acta1 | -2.92 |
| A_51_P135618 | Dlk1 | -2.92 |
| A_51_P156113 | Igsf9 | -2.91 |
| A_52_P173453 | LOC673676 | -2.91 |
| A_52_P189228 | TC1530514 | -2.90 |
| A_52_P860515 | Dirc2 | -2.89 |
| A_52_P652289 | AK032921 | -2.88 |
| A_51_P117369 | Phf8 | -2.87 |
| A_51_P230142 | A930018P22Rik | -2.86 |
| A_51_P264084 | Rab36 | -2.86 |
| A_51_P145883 | Btk | -2.85 |
| A_51_P466448 | BC035295 | -2.84 |
| A_52_P93380 | Als2cr12 | -2.84 |
| A_51_P286488 | Ier3 | -2.83 |
| A_51_P120636 | E2f1 | -2.83 |
| A_51_P138378 | Fosb | -2.82 |
| A_51_P227275 | ENSMUST00000001667 | -2.82 |
| A_51_P513803 | Fbxo4 | -2.81 |
| A_52_P818642 | 5830469G19Rik | -2.80 |
| A_52_P389235 | 3321401G04Rik | -2.79 |
| A_51_P458451 | Adipoq | -2.79 |
| A_52_P565636 | AY172876 | -2.79 |
| A_51_P274488 | Pcdh8 | -2.79 |
| A_51_P325862 | Hrasls | -2.79 |
| A_52_P653565 | ENSMUST00000084870 | -2.79 |
| A_52_P213889 | Tmc7 | -2.78 |
| A_52_P409601 | Cd40 | -2.78 |
| A_52_P130899 | Myh9 | -2.78 |
| A_52_P851352 | AK045003 | -2.77 |
| A_52_P818828 | AK033846 | -2.77 |
| A_51_P199772 | Gm505 | -2.77 |
| A_51_P449995 | C6 | -2.77 |
| A_51_P503671 | Syt13 | -2.75 |
| A_51_P197038 | Aftph | -2.75 |
| A_52_P92347 | Rab5b | -2.74 |
| A_52_P77764 | Slc7a2 | -2.74 |
| A_51_P366811 | Apod | -2.73 |
| A_52_P539454 | AK087958 | -2.73 |
| A_52_P217211 | Sertad1 | -2.72 |
| A_52_P440836 | Egr3 | -2.71 |
| A_51_P404815 | Apol6 | -2.71 |
| A_52_P503308 | 2700097O09Rik | -2.71 |
| A_51_P116496 | BC024139 | -2.70 |
| A_52_P447323 | Pscd2 | -2.70 |
| A_52_P32733 | Nfkb1 | -2.70 |
| A_51_P509997 | Cox6a2 | -2.69 |
| A_51_P185229 | Bmp2 | -2.69 |
| A_51_P375558 | Myoc | -2.68 |
| A_51_P205008 | Lst1 | -2.68 |
| A_52_P540399 | Acd | -2.67 |
| A_52_P988677 | Acot9 | -2.67 |
| A_52_P203322 | Rapgef1 | -2.67 |
| A_52_P215207 | A630077J23Rik | -2.66 |
| A_51_P374752 | Rbp4 | -2.66 |
| A_52_P298593 | C920006C10Rik | -2.65 |
| A_52_P307628 | Foxp4 | -2.65 |
| A_52_P396522 | Uqcrq | -2.65 |
| A_52_P334329 | 1500041B16Rik | -2.64 |
| A_51_P142421 | Rspo1 | -2.64 |
| A_51_P514270 | Add2 | -2.64 |
| A_51_P267836 | Plg | -2.64 |
| A_52_P68261 | Pde6h | -2.64 |
| A_51_P495780 | S3-12 | -2.64 |
| A_52_P313291 | Dgkb | -2.63 |
| A_52_P391505 | Gtl2 | -2.63 |
| A_51_P185593 | Dmn | -2.63 |
| A_52_P49797 | TC1533348 | -2.63 |
| A_52_P458169 | Adm2 | -2.62 |
| A_52_P1052476 | Bub3 | -2.62 |
| A_52_P398941 | Pde4d | -2.62 |
| A_52_P487615 | BC052328 | -2.62 |
| A_52_P802796 | AK047643 | -2.61 |
| A_52_P457312 | Fat3 | -2.61 |
| A_52_P2791 | Mtm1 | -2.60 |
| A_51_P184508 | Speer5-ps1 | -2.60 |
| A_52_P174962 | Ube2e3 | -2.60 |
| A_52_P192085 | LOC240444 | -2.60 |
| A_51_P297925 | Zc3h12a | -2.59 |
| A_51_P312859 | AI840671 | -2.59 |
| A_52_P423810 | Mt1 | -2.59 |
| A_51_P482508 | AK033099 | -2.59 |
| A_51_P406466 | Ankrd35 | -2.58 |
| A_52_P1075998 | AK039892 | -2.58 |
| A_52_P329207 | Expi | -2.58 |
| A_52_P971150 | 2210008N01Rik | -2.57 |
| A_52_P851306 | AK044145 | -2.57 |
| A_52_P26794 | Basp1 | -2.57 |
| A_51_P322933 | AK084126 | -2.56 |
| A_52_P27576 | Stxbp3a | -2.56 |
| A_52_P362772 | AK048349 | -2.56 |
| A_52_P1028214 | Pitpnc1 | -2.56 |
| A_52_P1084106 | AK047888 | -2.56 |
| A_51_P217465 | C79267 | -2.55 |
| A_51_P266958 | Nr1i2 | -2.55 |
| A_51_P455694 | Gabpb2 | -2.55 |
| A_52_P380399 | Stab1 | -2.55 |
| A_52_P600492 | Kalrn | -2.54 |
| A_51_P390181 | A430061O12Rik | -2.54 |
| A_51_P383032 | Clec4d | -2.54 |
| A_51_P473179 | Atp7a | -2.54 |
| A_52_P9392 | NAP037484-1 | -2.54 |
| A_52_P932202 | AK089833 | -2.54 |
| A_52_P453374 | 1110067I12Rik | -2.53 |
| A_52_P534560 | Frmd6 | -2.53 |
| A_51_P137029 | Plce1 | -2.53 |
| A_51_P466298 | 4921509E07Rik | -2.52 |
| A_51_P312896 | Spag9 | -2.52 |
| A_52_P631901 | Gabrb3 | -2.52 |
| A_52_P585350 | Etf1 | -2.52 |
| A_52_P552870 | 1700001O22Rik | -2.52 |
| A_51_P468558 | Treml4 | -2.51 |
| A_52_P8913 | Mobkl2a | -2.51 |
| A_52_P105520 | AK083356 | -2.51 |
| A_51_P415207 | 3110082D06Rik | -2.51 |
| A_51_P318637 | B3galnt1 | -2.50 |
| A_52_P615051 | 1200016E24Rik | -2.50 |
| A_52_P1180315 | AK049088 | -2.50 |
| A_52_P22627 | Gm276 | -2.50 |
| A_51_P424959 | Bcl6b | -2.50 |
| A_52_P359088 | Slc25a25 | -2.50 |
| A_51_P112405 | Plaur | -2.49 |
| A_51_P241457 | Lilrb4 | -2.49 |
| A_51_P388628 | Bhlhb8 | -2.49 |
| A_51_P175284 | AK053143 | -2.49 |
| A_51_P264922 | LOC641050 | -2.49 |
| A_51_P328725 | Snf1lk | -2.49 |
| A_51_P515108 | C030009O12Rik | -2.48 |
| A_52_P614487 | Git2 | -2.48 |
| A_51_P240404 | A830010M20Rik | -2.48 |
| A_51_P263137 | A_51_P263137 | -2.47 |
| A_51_P109840 | Vtn | -2.47 |
| A_52_P604345 | AK049762 | -2.47 |
| A_52_P335064 | Mustn1 | -2.47 |
| A_52_P677423 | TC1452905 | -2.47 |
| A_51_P193925 | Mapk6 | -2.46 |
| A_51_P508602 | Tcp10c | -2.46 |
| A_52_P639461 | Car3 | -2.46 |
| A_51_P228574 | Tat | -2.46 |
| A_51_P285279 | Slc25a2 | -2.45 |
| A_51_P259603 | Adcyap1r1 | -2.45 |
| A_51_P376959 | Tmeff2 | -2.45 |
| A_51_P238665 | Otop3 | -2.45 |
| A_52_P57776 | Nrbp2 | -2.45 |
| A_52_P231762 | ENSMUST00000076384 | -2.44 |
| A_52_P1051947 | AK035671 | -2.44 |
| A_51_P235945 | Hp | -2.44 |
| A_51_P184041 | C330023D02Rik | -2.44 |
| A_51_P182708 | BC034204 | -2.43 |
| A_51_P514035 | Cma1 | -2.43 |
| A_51_P395050 | Wdtc1 | -2.42 |
| A_52_P867738 | AK042489 | -2.42 |
| A_52_P213932 | Adamts1 | -2.42 |
| A_51_P179543 | AI316844 | -2.42 |
| A_52_P562911 | M19902 | -2.42 |
| A_52_P628615 | Fyb | -2.41 |
| A_52_P12869 | Psmd4 | -2.41 |
| A_51_P118688 | Oprm1 | -2.41 |
| A_51_P490456 | Cidec | -2.41 |
| A_52_P891503 | AK043234 | -2.41 |
| A_51_P488739 | Gpr109a | -2.40 |
| A_51_P288459 | ENSMUST00000060684 | -2.40 |
| A_52_P714766 | AK032904 | -2.40 |
| A_51_P412809 | Dqx1 | -2.40 |
| A_51_P303147 | 1700113O17Rik | -2.40 |
| A_51_P376656 | Synpr | -2.40 |
| A_52_P763389 | B930095M22Rik | -2.39 |
| A_51_P108226 | 1100001G20Rik | -2.39 |
| A_51_P469595 | AK037470 | -2.39 |
| A_51_P387591 | Nfkbiz | -2.39 |
| A_52_P20391 | CB182661 | -2.39 |
| A_51_P312485 | Fpr1 | -2.38 |
| A_52_P223446 | Itsn2 | -2.38 |
| A_52_P592007 | Tfpi | -2.38 |
| A_51_P268193 | Slc7a10 | -2.38 |
| A_52_P625461 | Ipo11 | -2.38 |
| A_52_P269630 | AB022157 | -2.37 |
| A_51_P234663 | Cacnb4 | -2.37 |
| A_51_P346964 | Mrap | -2.37 |
| A_52_P618253 | Myo1h | -2.37 |
| A_52_P591740 | CX563319 | -2.37 |
| A_51_P439426 | Acaca | -2.36 |
| A_51_P286423 | Foxd3 | -2.36 |
| A_51_P192964 | 4933425B16Rik | -2.36 |
| A_52_P246698 | Drctnnb1a | -2.36 |
| A_52_P8391 | Cpn1 | -2.36 |
| A_52_P624572 | Bmpr1a | -2.36 |
| A_52_P362161 | Rab3b | -2.36 |
| A_51_P328951 | Sgip1 | -2.36 |
| A_52_P164172 | Mlstd2 | -2.36 |
| A_52_P346556 | AK038627 | -2.36 |
| A_51_P385701 | Abca9 | -2.36 |
| A_51_P378051 | Aoc3 | -2.35 |
| A_52_P1196772 | AK087259 | -2.34 |
| A_51_P164043 | U26471 | -2.34 |
| A_52_P320686 | TC1531727 | -2.34 |
| A_51_P363862 | AK039616 | -2.34 |
| A_51_P175784 | Neurog3 | -2.34 |
| A_52_P663828 | Myh14 | -2.34 |
| A_52_P532982 | Gdf15 | -2.34 |
| A_51_P177210 | Myl3 | -2.33 |
| A_52_P356419 | Kctd11 | -2.33 |
| A_52_P545472 | 4932417H02Rik | -2.33 |
| A_52_P795267 | AK033022 | -2.33 |
| A_51_P481463 | Dync1li1 | -2.33 |
| A_52_P347031 | B430203M17Rik | -2.32 |
| A_52_P218132 | Top1 | -2.32 |
| A_51_P108228 | Chrna7 | -2.32 |
| A_51_P448894 | 4930597L12Rik | -2.32 |
| A_52_P5579 | 4930455C13Rik | -2.32 |
| A_51_P481592 | Ckap2 | -2.32 |
| A_52_P446724 | TC1493944 | -2.31 |
| A_52_P495759 | Rarb | -2.31 |
| A_52_P957659 | Igk-V1 | -2.31 |
| A_51_P402144 | 2300002M23Rik | -2.31 |
| A_51_P275005 | Fut4-ps1 | -2.31 |
| A_51_P301627 | AK047604 | -2.31 |
| A_51_P169795 | Sbp | -2.30 |
| A_52_P368192 | N28178 | -2.30 |
| A_51_P222264 | BC026657 | -2.30 |
| A_52_P371143 | Mipol1 | -2.30 |
| A_52_P405145 | Flt1 | -2.30 |
| A_51_P515985 | U55685 | -2.30 |
| A_51_P103706 | Cyp2c29 | -2.30 |
| A_51_P199339 | D230004J03Rik | -2.30 |
| A_52_P548011 | D230021J17Rik | -2.30 |
| A_51_P290619 | AI324046 | -2.29 |
| A_52_P631728 | Ankrd44 | -2.29 |
| A_52_P323484 | Islr | -2.29 |
| A_51_P174961 | F10 | -2.29 |
| A_52_P68440 | TC1516668 | -2.29 |
| A_52_P78514 | AK054415 | -2.29 |
| A_52_P152847 | Inhbb | -2.29 |
| A_52_P525161 | Ntf3 | -2.29 |
| A_52_P778761 | 1700110N18Rik | -2.29 |
| A_51_P401263 | Eme1 | -2.29 |
| A_51_P354345 | Zpbp | -2.28 |
| A_52_P31641 | Tmem145 | -2.28 |
| A_51_P130497 | Cnbp2 | -2.28 |
| A_52_P145861 | Cd86 | -2.27 |
| A_52_P142143 | Jph2 | -2.27 |
| A_52_P851837 | AK083477 | -2.27 |
| A_52_P625218 | Sorcs3 | -2.27 |
| A_52_P584570 | NAP058430-1 | -2.27 |
| A_52_P593534 | Atg3 | -2.27 |
| A_52_P348250 | Kbtbd9 | -2.27 |
| A_52_P126782 | Ggtla1 | -2.27 |
| A_52_P674331 | 3222401L13Rik | -2.27 |
| A_51_P210992 | 4930506C21Rik | -2.26 |
| A_51_P296249 | Fkhl18 | -2.26 |
| A_52_P223414 | Slc13a1 | -2.26 |
| A_52_P189455 | Vps18 | -2.26 |
| A_51_P388329 | Aff3 | -2.26 |
| A_51_P213045 | Smoc2 | -2.26 |
| A_52_P298584 | Bicd1 | -2.26 |
| A_52_P34979 | Epc1 | -2.25 |
| A_51_P263246 | Dusp8 | -2.25 |
| A_52_P374545 | Gimap3 | -2.25 |
| A_51_P259631 | Stk22s1 | -2.25 |
| A_52_P657722 | Ahctf1 | -2.25 |
| A_52_P519960 | Siglec1 | -2.25 |
| A_52_P162637 | LOC669091 | -2.25 |
| A_51_P407657 | Igk-V38 | -2.25 |
| A_52_P293482 | Lgals12 | -2.25 |
| A_52_P313933 | 1700057H21Rik | -2.25 |
| A_51_P401775 | AK048206 | -2.25 |
| A_51_P336325 | Orm1 | -2.25 |
| A_51_P478722 | Tgtp | -2.24 |
| A_52_P612668 | AK085466 | -2.24 |
| A_52_P637210 | AU018823 | -2.24 |
| A_52_P268206 | Mcam | -2.24 |
| A_52_P811736 | Atp1b3 | -2.24 |
| A_52_P261722 | Rfx1 | -2.24 |
| A_52_P86750 | B230208H17Rik | -2.24 |
| A_52_P765764 | Igkv1-132 | -2.24 |
| A_52_P55972 | Retn | -2.24 |
| A_52_P460285 | NAP058254-1 | -2.24 |
| A_52_P58494 | Tusc5 | -2.23 |
| A_51_P262616 | 4930505D03Rik | -2.23 |
| A_51_P321449 | Tnp2 | -2.23 |
| A_51_P219396 | Prh1 | -2.23 |
| A_52_P616600 | Rusc2 | -2.23 |
| A_51_P400311 | 4930550C14Rik | -2.23 |
| A_51_P253691 | AK084357 | -2.23 |
| A_51_P199314 | Slc38a2 | -2.22 |
| A_52_P531606 | ENSMUST00000063645 | -2.22 |
| A_51_P458080 | Osbp2 | -2.22 |
| A_52_P191059 | Zfp612 | -2.22 |
| A_51_P150996 | 6720422M22Rik | -2.22 |
| A_52_P385824 | Sema5b | -2.22 |
| A_52_P640337 | Gng5 | -2.22 |
| A_51_P166706 | AK028934 | -2.22 |
| A_52_P243658 | Edil3 | -2.22 |
| A_51_P139978 | Cmar | -2.21 |
| A_52_P200286 | 2810003K23Rik | -2.21 |
| A_52_P589321 | LOC14433 | -2.21 |
| A_52_P1180839 | AK087344 | -2.21 |
| A_51_P327511 | Fgl2 | -2.21 |
| A_52_P104961 | ENSMUST00000030352 | -2.21 |
| A_52_P1045316 | Gm428 | -2.21 |
| A_52_P287772 | AK052728 | -2.21 |
| A_52_P127842 | TC1484949 | -2.21 |
| A_51_P451458 | Mamdc2 | -2.21 |
| A_51_P425642 | Grasp | -2.21 |
| A_52_P487040 | Rdh9 | -2.20 |
| A_51_P143712 | Tdh | -2.20 |
| A_52_P112219 | ENSMUST00000056774 | -2.20 |
| A_52_P424778 | Adra1a | -2.20 |
| A_51_P262489 | Sst | -2.20 |
| A_52_P1131944 | A130019P10Rik | -2.20 |
| A_51_P320261 | 4933406B15Rik | -2.20 |
| A_52_P644903 | Api5 | -2.20 |
| A_51_P286426 | AK019053 | -2.20 |
| A_51_P199041 | Adcy5 | -2.20 |
| A_52_P988322 | AK082037 | -2.20 |
| A_51_P113769 | AK037868 | -2.19 |
| A_52_P136138 | Fdft1 | -2.19 |
| A_51_P484027 | Cd300d | -2.19 |
| A_51_P418488 | Robo2 | -2.19 |
| A_51_P490397 | Pold3 | -2.19 |
| A_51_P415388 | AK080389 | -2.19 |
| A_52_P355934 | Myo1b | -2.19 |
| A_52_P335590 | 5730458M16Rik | -2.19 |
| A_51_P427603 | BC018242 | -2.19 |
| A_51_P430903 | AK029875 | -2.18 |
| A_52_P225856 | A130038J17Rik | -2.18 |
| A_51_P473498 | Gpr171 | -2.18 |
| A_52_P997209 | TC1448906 | -2.18 |
| A_51_P356916 | Nup98 | -2.18 |
| A_52_P481957 | Grem1 | -2.17 |
| A_51_P100991 | Gucy2c | -2.17 |
| A_52_P498396 | Neil2 | -2.17 |
| A_52_P354947 | E230008O15Rik | -2.17 |
| A_52_P593737 | Crkrs | -2.17 |
| A_51_P268748 | 2610203C20Rik | -2.17 |
| A_51_P234944 | Adamts4 | -2.16 |
| A_51_P188527 | AK053162 | -2.16 |
| A_51_P230269 | H2-Q10 | -2.16 |
| A_51_P208083 | S100b | -2.16 |
| A_51_P144531 | D630002G06Rik | -2.16 |
| A_52_P245119 | Slc16a1 | -2.16 |
| A_52_P293248 | Dock4 | -2.16 |
| A_51_P222280 | Ikbke | -2.16 |
| A_52_P780667 | Rps4x | -2.15 |
| A_51_P454529 | AK037256 | -2.15 |
| A_52_P103590 | Dpy19l1 | -2.15 |
| A_51_P354165 | Apcs | -2.15 |
| A_52_P939146 | AK034770 | -2.15 |
| A_51_P458973 | Pygm | -2.15 |
| A_51_P104430 | Magee1 | -2.15 |
| A_51_P335321 | Gnas | -2.15 |
| A_52_P181748 | 9630055N22Rik | -2.15 |
| A_51_P156955 | Cfd | -2.15 |
| A_52_P819877 | AK086516 | -2.15 |
| A_52_P468851 | 5830436D01Rik | -2.15 |
| A_52_P195235 | 4930555G01Rik | -2.14 |
| A_52_P370274 | Pde4b | -2.14 |
| A_52_P511781 | Slc36a1 | -2.14 |
| A_52_P79196 | AK122525 | -2.14 |
| A_52_P1156254 | AK042218 | -2.14 |
| A_51_P351896 | 1110032E23Rik | -2.14 |
| A_52_P621617 | Dnajb4 | -2.14 |
| A_52_P355751 | LOC544988 | -2.14 |
| A_51_P364185 | E030017K20 | -2.14 |
| A_52_P262930 | 2310081J21Rik | -2.14 |
| A_52_P655278 | Mas1 | -2.14 |
| A_51_P485472 | 4933430H15Rik | -2.13 |
| A_52_P180210 | Rab18 | -2.13 |
| A_51_P415555 | Amy2 | -2.13 |
| A_51_P453446 | Ror1 | -2.13 |
| A_52_P674067 | D11Bwg0517e | -2.13 |
| A_51_P437349 | Mds1 | -2.13 |
| A_52_P248920 | Grin2b | -2.13 |
| A_52_P351638 | Tnfrsf10b | -2.13 |
| A_52_P657360 | Tnni1 | -2.13 |
| A_51_P247249 | Alox5 | -2.13 |
| A_52_P875299 | AK034190 | -2.13 |
| A_51_P223058 | Cgn | -2.13 |
| A_51_P152990 | Grem2 | -2.13 |
| A_52_P87513 | NAP101973-1 | -2.12 |
| A_51_P178319 | Arl5b | -2.12 |
| A_51_P272363 | Mcoln3 | -2.12 |
| A_52_P614453 | 5430410E06Rik | -2.12 |
| A_52_P1035759 | Eif4e | -2.12 |
| A_52_P98210 | ENSMUST00000042017 | -2.12 |
| A_52_P457853 | 2210409E12Rik | -2.12 |
| A_51_P288643 | BC006028 | -2.12 |
| A_52_P239381 | E330009E22Rik | -2.11 |
| A_52_P89125 | AK042446 | -2.11 |
| A_51_P476518 | 2310040C09Rik | -2.11 |
| A_51_P321250 | AK031195 | -2.11 |
| A_52_P47126 | Maml3 | -2.11 |
| A_52_P65408 | Ctnnal1 | -2.11 |
| A_52_P498718 | AK046053 | -2.10 |
| A_51_P240760 | B3gnt3 | -2.10 |
| A_51_P502614 | Dusp6 | -2.10 |
| A_51_P389864 | C76566 | -2.10 |
| A_52_P451355 | St6galnac3 | -2.10 |
| A_52_P899303 | B230214O09Rik | -2.10 |
| A_51_P503162 | Klf6 | -2.10 |
| A_52_P987292 | 6330509M23Rik | -2.10 |
| A_51_P181286 | Cd69 | -2.10 |
| A_52_P1156808 | Atp2a2 | -2.10 |
| A_52_P263644 | Eif2s2 | -2.09 |
| A_52_P472324 | Slpi | -2.09 |
| A_52_P594756 | Asb4 | -2.09 |
| A_51_P182728 | Stx1a | -2.09 |
| A_52_P154101 | Calca | -2.09 |
| A_51_P334979 | Apoc2 | -2.09 |
| A_51_P404385 | AK081913 | -2.09 |
| A_52_P30386 | AK084172 | -2.09 |
| A_51_P517075 | Serpinf1 | -2.09 |
| A_51_P274173 | Ldlr | -2.09 |
| A_52_P51743 | AK053800 | -2.08 |
| A_52_P231428 | A430107O13Rik | -2.08 |
| A_52_P386932 | AK039987 | -2.08 |
| A_52_P443334 | Cd8a | -2.08 |
| A_51_P182490 | C630028L02Rik | -2.08 |
| A_51_P435731 | E130112L23Rik | -2.08 |
| A_52_P647260 | Synpo2 | -2.08 |
| A_51_P401184 | Rarres1 | -2.08 |
| A_52_P498128 | Ccdc94 | -2.08 |
| A_51_P139765 | E130006N16Rik | -2.07 |
| A_52_P282058 | Col8a1 | -2.07 |
| A_52_P48681 | Cldn1 | -2.07 |
| A_52_P932002 | AK079562 | -2.07 |
| A_52_P835749 | AK051598 | -2.07 |
| A_52_P131367 | Zc3hav1 | -2.07 |
| A_52_P1171934 | A430106J12Rik | -2.07 |
| A_51_P257583 | Taf15 | -2.07 |
| A_51_P437707 | Cldn18 | -2.07 |
| A_52_P226476 | Kif7 | -2.07 |
| A_51_P153368 | Slc6a1 | -2.07 |
| A_52_P249544 | Rufy3 | -2.07 |
| A_52_P123772 | AK037161 | -2.07 |
| A_51_P320022 | Atp10a | -2.06 |
| A_52_P357745 | Ypel2 | -2.06 |
| A_52_P412796 | NAP043943-1 | -2.06 |
| A_51_P106538 | Htra3 | -2.06 |
| A_52_P590936 | Mthfr | -2.06 |
| A_52_P36065 | Tdrd5 | -2.06 |
| A_52_P651101 | LOC629678 | -2.06 |
| A_52_P86892 | G630049C14Rik | -2.06 |
| A_52_P448936 | Ppp2r5e | -2.06 |
| A_52_P628870 | Col27a1 | -2.06 |
| A_51_P155843 | Igsf10 | -2.06 |
| A_51_P468707 | AK078992 | -2.06 |
| A_52_P563285 | Col11a2 | -2.06 |
| A_51_P266248 | ENSMUST00000101001 | -2.05 |
| A_51_P142107 | Fzd7 | -2.05 |
| A_52_P213722 | D330010C22Rik | -2.05 |
| A_52_P560578 | X75105 | -2.05 |
| A_52_P88054 | Chrm4 | -2.05 |
| A_52_P184588 | Slc2a9 | -2.05 |
| A_51_P176396 | 4631427C17Rik | -2.05 |
| A_52_P771228 | AK054284 | -2.05 |
| A_51_P437327 | Ascl1 | -2.05 |
| A_52_P342880 | Itsn1 | -2.05 |
| A_52_P892091 | A030007N12Rik | -2.05 |
| A_52_P648824 | X12388 | -2.05 |
| A_52_P374157 | Kbtbd8 | -2.05 |
| A_52_P596635 | Dars | -2.05 |
| A_52_P979844 | AK034548 | -2.05 |
| A_52_P26265 | AB041803 | -2.05 |
| A_52_P166833 | Snx5 | -2.05 |
| A_52_P607060 | Nebl | -2.04 |
| A_51_P189777 | Ccrn4l | -2.04 |
| A_52_P348704 | Dnajb5 | -2.04 |
| A_51_P344461 | 2900092E17Rik | -2.04 |
| A_52_P375069 | Parp3 | -2.04 |
| A_52_P27803 | AK082254 | -2.04 |
| A_52_P268880 | 4631403P03Rik | -2.04 |
| A_52_P723233 | BB450286 | -2.03 |
| A_52_P158282 | St6galnac4 | -2.03 |
| A_52_P732433 | A_52_P732433 | -2.03 |
| A_52_P245577 | TC1503683 | -2.03 |
| A_51_P268094 | Serpine2 | -2.03 |
| A_51_P347673 | Kcnj9 | -2.03 |
| A_52_P169181 | Auts2 | -2.03 |
| A_51_P179828 | Ppm1f | -2.03 |
| A_51_P231036 | Col9a1 | -2.03 |
| A_52_P148571 | Trim24 | -2.03 |
| A_52_P18937 | 4930404N11Rik | -2.03 |
| A_51_P508156 | Cryga | -2.03 |
| A_52_P143671 | E130306D19Rik | -2.03 |
| A_51_P131408 | Tnfrsf12a | -2.03 |
| A_52_P319073 | Lrrc57 | -2.03 |
| A_52_P483339 | Aars | -2.02 |
| A_52_P573552 | Trib1 | -2.02 |
| A_52_P356093 | B3galt2 | -2.02 |
| A_52_P209973 | 0610038F07Rik | -2.02 |
| A_51_P349673 | Rab37 | -2.02 |
| A_51_P494812 | AK038312 | -2.02 |
| A_52_P268127 | Hace1 | -2.02 |
| A_52_P227267 | Atp1a2 | -2.02 |
| A_52_P188215 | 3110047P20Rik | -2.02 |
| A_52_P416499 | Enpp2 | -2.02 |
| A_52_P559975 | Il8rb | -2.02 |
| A_51_P224311 | Popdc2 | -2.02 |
| A_52_P384203 | Yme1l1 | -2.02 |
| A_52_P291428 | 1700001E04Rik | -2.02 |
| A_52_P246248 | AF240166 | -2.02 |
| A_51_P454943 | Gpr19 | -2.02 |
| A_52_P433029 | Fndc1 | -2.02 |
| A_52_P237241 | ENSMUST00000095675 | -2.01 |
| A_52_P553786 | ENSMUST00000038890 | -2.01 |
| A_51_P266733 | ENSMUST00000098498 | -2.01 |
| A_52_P223626 | Olig2 | -2.01 |
| A_52_P597277 | NAP102624-1 | -2.01 |
| A_51_P273433 | Prkg2 | -2.01 |
| A_51_P375526 | Pax2 | -2.01 |
| A_52_P668071 | Asb1 | -2.01 |
| A_52_P640686 | Cit | -2.01 |
| A_52_P33147 | A730017D01Rik | -2.01 |
| A_52_P31722 | ENSMUST00000048275 | -2.01 |
| A_51_P410900 | Fut10 | -2.01 |
| A_52_P496986 | AK041471 | -2.01 |
| A_52_P144954 | Skp2 | -2.00 |
| A_51_P293656 | Rrn3 | -2.00 |
| A_52_P496956 | Acsbg1 | -2.00 |
