# Supplementary material for: Aqp5 Is a New Transcriptional Target of Dot1a and a Regulator of Aqp2
Source: PLoS One. 2013 Jan 10;8(1):e53342. doi: 10.1371/journal.pone.0053342 (PMC3542343; doi:10.1371/journal.pone.0053342)
Supplement: Table S3 — Primers for real-time RT-qPCR and Dot1a-specific siRNA. Listed are sequences of the primers used for real-time RT-qPCR and Dot1a-specific siRNA. F: Forward. R: Reverse. (DOC) [file pone.0053342.s007.doc]

**Table S3. Primers for real-time RT-qPCR and Dot1a-specific siRNA.** Listed are sequences of the primers used for real-time RT-qPCR and Dot1a-specific siRNA. F: Forward. R: Reverse.

|  | **Sequence (5’→3’)** | **Gene** | **Length** |
| --- | --- | --- | --- |
| WZ352 | F: CAACAGCAGGAACTTGAGTGACATTGGC | Dot1a | 515 bp |
| WZ359 | R: ACGCATCCTGGGGTGAGGCTGAGGG |
| WZ1094 | F: ATGTGGGAACTCCGGTCCATA | Aqp2 | 137 bp |
| WZ1095 | R: ACGGCAATCTGGAGCACAG |
| WZ1096 | F: GCTTTTGGCTTCGCTGTCAC | Aqp3 | 128 bp |
| WZ1097 | R: TAGATGGGCAGCTTGATCCAG |
| WZ1106 | F: AGGTCGGTGTGAACGGATTTG | GAPDH | 123 bp |
| WZ1107 | R: TGTAGACCATGTAGTTGAGGTCA |
| WZ1211 | F: CTCAGCAACAACACAACACCAGGC | Aqp5 | 318 bp |
| WZ1212 | R: GGGGAAAAGCAAGTAGAAGTAGAGGATTG |
| WZ517 | F: GTGGGCCGCTCTAGGCACCAA | β-Actin | 539 bp |
| WZ518 | R: CTCTTTGATGTCACGCACGATTTC |
| WZ1265 | UUUCCAUACCAUUUCAUCCdTdT | siRNA#1 |  |
| WZ1266 | GAGUUCAGGAAGUGGAUGAdTdT | siRNA#2 |  |
