# Supplementary material for: Aqp5 Is a New Transcriptional Target of Dot1a and a Regulator of Aqp2
Source: PLoS One. 2013 Jan 10;8(1):e53342. doi: 10.1371/journal.pone.0053342 (PMC3542343; doi:10.1371/journal.pone.0053342)
Supplement: Table S4 — Primers for ChIP in Aqp5 5′ flanking region. Listed are sequences of the primers in Aqp5 5′ flanking region. These primers were used in chromatin immunoprecipitation coupled with real-time qPCR analyses. F: Forward. R: Reverse. (DOC) [file pone.0053342.s008.doc]

**Table S4. Primers for ChIP in Aqp5 5’ flanking region.** Listed are sequences of the primers in Aqp5 5’ flanking region. These primers were used in chromatin immunoprecipitation coupled with real-time qPCR analyses. F: Forward. R: Reverse.

|  | **Sequence (5’→3’)** | **Subregion** | **Length** |
| --- | --- | --- | --- |
| WZ1291 | F: TCCACTTCCTCTGCTACAGATGAGTCC | Subregion A | 623 bp |
| WZ1292 | R: GGAAGAGGGTGGAGCATAGGGCAG |
| WZ1293 | F: CTGCCCTATGCTCCACCCTCTTCC | Subregion B | 523 bp |
| WZ1294 | R: GAGAGTGTGGGGGTGACACAGGGC |
| WZ1295 | F: GCCCTGTGTCACCCCCACACTCTC | Subregion C | 482 bp |
| WZ1296 | R: TTAGCACAGATAAAAGTTGCTGGGACG |
| WZ1297 | F: CGTCCCAGCAACTTTTATCTGTGCTAA | Subregion D | 524 bp |
| WZ1298 | R: TGGGGTCTGGACTGGGCTGGAT |
| WZ1299 | F: GGCTGATCCAGCCCAGTCCAGAC | Subregion E | 491 bp |
| WZ1300 | R: AACCCCGATGTATTGACAAACGAG |
| WZ1301 | F: CTCGTTTGTCAATACATCGGGGTT | Subregion F | 622 bp |
| WZ1302 | R: GGCACTTTTGGGTAACAATGGGGC |
| WZ1303 | F: GCCCCATTGTTACCCAAAAGTGCC | Subregion G | 510 bp |
| WZ1304 | R: TAGAAGGGGGGACTCACTGGCTCAG |
| WZ1305 | F: GAAGCCTTAAGCAAATGTTTATTGCC | Subregion H | 524 bp |
| WZ1306 | R: CAAGGAGTCGTTTTGGTGTAACTAAGG |
| WZ1307 | F: CCTTAGTTACACCAAAACGACTCCTTG | Subregion I | 526 bp |
| WZ1308 | R: CTGTCCTTTTAGAGCGGGCTGGTC |
| WZ1309 | F: GACCAGCCCGCTCTAAAAGGACAG | Subregion J | 523 bp |
| WZ1310 | R: CTTTGCACCGTTGCACCGTACCAG |
| WZ1313 | F: CAAAGGGTGATAGACTGAGGAAAAGAGA | Subregion K | 525 bp |
| WZ1314 | R: CTCTTAGCTCCTTATCCGCCCGT |
| WZ1315 | F: GCCACGGGCGGATAAGGAGCTAAG | Subregion L | 520 bp |
| WZ1316 | R: CGGGCAGGGGCGCGCTATATA |
